# Severe consequences of habitat fragmentation on genetic diversity of an endangered Australian freshwater fish: A call for assisted gene flow

**DOI:** 10.1111/eva.12484

**Published:** 2017-05-11

**Authors:** Alexandra Pavlova, Luciano B. Beheregaray, Rhys Coleman, Dean Gilligan, Katherine A. Harrisson, Brett A. Ingram, Joanne Kearns, Annika M. Lamb, Mark Lintermans, Jarod Lyon, Thuy T. T. Nguyen, Minami Sasaki, Zeb Tonkin, Jian D. L. Yen, Paul Sunnucks

**Affiliations:** ^1^ School of Biological SciencesClayton Campus, Monash UniversityClaytonVICAustralia; ^2^ School of Biological SciencesFlinders UniversityAdelaideSAAustralia; ^3^ Applied ResearchMelbourne WaterDocklandsVICAustralia; ^4^ Freshwater Ecosystems ResearchNSW Department of Primary Industries – FisheriesBatemans BayNSWAustralia; ^5^ Department of Environment, Land Water and PlanningArthur Rylah Institute, Land, Fire and EnvironmentHeidelbergVICAustralia; ^6^ Department of Ecology Environment and EvolutionSchool of Life Sciences, La Trobe UniversityBundoora, Victoria3083Australia; ^7^ Department of Economic DevelopmentJobs, Transport and ResourcesFisheries VictoriaAlexandraVICAustralia; ^8^ Institute for Applied EcologyUniversity of CanberraCanberraACTAustralia; ^9^ Agriculture VictoriaAgriBio, Centre for AgriBioscienceBundooraVICAustralia; ^10^ School of Physics and AstronomyClayton Campus, Monash UniversityClaytonVICAustralia

**Keywords:** adaptive potential, effective population size, genetic rescue, genetic restoration, inbreeding depression, Macquarie perch *Macquaria australasica*, management, population persistence

## Abstract

Genetic diversity underpins the ability of populations to persist and adapt to environmental changes. Substantial empirical data show that genetic diversity rapidly deteriorates in small and isolated populations due to genetic drift, leading to reduction in adaptive potential and fitness and increase in inbreeding. Assisted gene flow (e.g. via translocations) can reverse these trends, but lack of data on fitness loss and fear of impairing population “uniqueness” often prevents managers from acting. Here, we use population genetic and riverscape genetic analyses and simulations to explore the consequences of extensive habitat loss and fragmentation on population genetic diversity and future population trajectories of an endangered Australian freshwater fish, Macquarie perch *Macquaria australasica*. Using guidelines to assess the risk of outbreeding depression under admixture, we develop recommendations for population management, identify populations requiring genetic rescue and/or genetic restoration and potential donor sources. We found that most remaining populations of Macquarie perch have low genetic diversity, and effective population sizes below the threshold required to retain adaptive potential. Our simulations showed that under management inaction, smaller populations of Macquarie perch will face inbreeding depression within a few decades, but regular small‐scale translocations will rapidly rescue populations from inbreeding depression and increase adaptive potential through genetic restoration. Despite the lack of data on fitness loss, based on our genetic data for Macquarie perch populations, simulations and empirical results from other systems, we recommend regular and frequent translocations among remnant populations within catchments. These translocations will emulate the effect of historical gene flow and improve population persistence through decrease in demographic and genetic stochasticity. Increasing population genetic connectivity within each catchment will help to maintain large effective population sizes and maximize species adaptive potential. The approach proposed here could be readily applicable to genetic management of other threatened species to improve their adaptive potential.

## 1 Introduction

A primary goal of conservation management is to improve the adaptive potential of populations, that is, the ability of populations to adapt and persist in the face of environmental changes (Sgrò, Lowe, & Hoffmann, 2011). Genetic diversity underpins adaptive potential, but is lost in small populations through genetic drift, which can lead to loss of fitness, accumulation of genetic load and ultimately population extinction (Harrisson, Pavlova, Telonis‐Scott, & Sunnucks, 2014; Reed & Frankham, 2003; Willi, Van Buskirk, & Hoffmann, 2006). Through reduction or cessation of gene flow, habitat fragmentation reduces species ranges to small populations at high extinction risk, contributed to by environmental, demographic and genetic factors (e.g. inbreeding depression, genetic load and inability to rapidly adapt to environmental changes) and their interactions (Benson et al., 2016; Fountain, Nieminen, Sirén, Wong, & Hanski, 2016; Frankham, 2005; Lopez, Rousset, Shaw, Shaw, & Ronce, 2009). Given that taxa of conservation concern have typically undergone habitat fragmentation and/or strong population contractions, genetic issues should be addressed in recovery or management plans, although this is often not done (Pierson et al., 2015, 2016).

Effective and efficient allocation of conservation resources is best assisted by information about the distribution of genetic variation across landscapes (Hoffmann et al., 2015). Spatial patterns of genetic diversity and genetic differentiation reflect stochastic and environmental effects on key demographic and evolutionary processes (e.g. population size, gene flow, adaptive potential) linked to viability. Thus, genetic data can inform about the conservation status of populations, improve predictions about population responses to environmental change and management interventions, and support the development of effective conservation strategies for enhancing population viability (Willi & Hoffmann, 2009).

For a given population or species, a direct demonstration of genetic problems, such as loss of fitness due to decline in genetic diversity or inbreeding, presents a strong case for genetic management. In contrast, unavailability of evidence for genetic problems in a specific case often prompts an argument against genetic intervention until such evidence is collected. This position ignores evidence acquired from numerous wild and captive systems that small and isolated populations will most likely be facing genetic problems with negative demographic consequences (Keller & Waller, 2002; Saccheri et al., 1998; Woodworth, Montgomery, Briscoe, & Frankham, 2002), reflecting cultural rather than evidence‐based decisions concerning genetic management (Love Stowell, Pinzone, & Martin, 2017). Obtaining data on population fitness is challenging and expensive and may take years. Postponing decision‐making until such data are available, or disregarding genetic problems completely, is likely to result in managing small populations to extinction (Frankham et al., in press; Love Stowell et al., 2017; Weeks, Stoklosa, & Hoffmann, 2016). Instead, the augmentation of gene flow is a powerful conservation management option for counteracting loss of genetic diversity resulting from drift in small populations, with the potential to promote positive demographic outcomes (Frankham, 2015; Hufbauer et al., 2015; Whiteley, Fitzpatrick, Funk, & Tallmon, 2015). Genetic augmentation to alleviate detrimental effects of inbreeding and/or genetic load in small, isolated populations (i.e. genetic rescue) and/or increase levels of genetic diversity and adaptive potential (i.e. genetic restoration) can be achieved through reconnection of habitat or human‐assisted translocations (Attard et al., 2016; Weeks et al., 2011; Whiteley et al., 2015). Strong precedents supporting the overwhelmingly positive and long‐lasting effects of genetic rescue on fitness exist across a broad range of taxa (Frankham, 2015, 2016). By bringing in new genetic diversity, genetic augmentation is expected to improve adaptive potential and probability of persistence of small and isolated populations, even if they do not yet suffer from inbreeding depression and genetic load (Willi et al., 2006).

Another common argument against genetic augmentation is that gene flow between divergent populations may “swamp” local adaptation and distinctiveness, or contribute to outbreeding depression (Frankham et al., 2011; Le Cam, Perrier, Besnard, Bernatchez, & Evanno, 2015; Love Stowell et al., 2017). Such concerns frequently lead to recommendations to maintain isolation of differentiated populations, but without appropriate assessment of the risks and benefits of gene flow (Farrington, Lintermans, & Ebner, 2014; Frankham et al., 2011; Roberts, Baker, & Perrin, 2011). Genetic differentiation per se is not a clear indicator of risk that populations are differently adapted and gene flow might be harmful: some differentiation is inevitable under restricted dispersal, particularly among small populations, and is often caused by recent human impacts (Cole et al., 2016; Coleman, Weeks, & Hoffmann, 2013; Faulks, Gilligan, & Beheregaray, 2011). Even where genetic differentiation is associated with adaptive divergence, locally adapted traits can still be maintained in a population in the presence of gene flow from a differently adapted population (Fitzpatrick, Gerberich, Kronenberger, Angeloni, & Funk, 2015), unless the level of gene flow is so large that it overwhelms natural selection (Lowe & Allendorf, 2010). The potential benefits of gene flow are large, and any risks of outbreeding depression must be weighed against the risk of extinction due to inaction, for example from inbreeding depression, genetic load or inability to adapt to a novel selective pressure (Becker, Tweedie, Gilligan, Asmus, & Whittington, 2013; Fisher, Garner, & Walker, 2009; Frankham et al., in press; Harrisson, Pavlova, et al., 2016; Lintermans, 2013).

In developing plans for genetic rescue and/or restoration, four key questions must be considered:

1. What level of genetic variation should trigger genetic rescue/restoration?
2. Which populations can be used as sources for translocations?
3. What is the risk of outbreeding depression? and
4. How much gene flow is required to rescue the populations at risk and restore their adaptive potential?

Recent reviews provide guidelines:

1. Effective population size (*N*_e_) ≥100 is required to prevent inbreeding depression in the short‐term and limit loss of fitness to ≤10% over 5 generations. A much larger global effective population size, *N*_e_ ≥ 1,000, is required to retain adaptive potential, by maintaining an equilibrium between the accumulation of genetic variation for a selectively neutral trait by mutation, and the loss variation by random genetic drift (Frankham, Bradshaw, & Brook, 2014). Based on a combination of theory and empirical evidence, the numbers from previously proposed 50/500 rule (Franklin, 1980) were found to be insufficient to prevent inbreeding depression/avoid loss of genetic variation for fitness (Frankham et al., 2014).
2. Any population only recently diverged from a genetically depleted one can be used as a source, but the magnitude of genetic rescue is greater if the source population is outbred and/or differentiated (Frankham, 2015).
3. The risk of outbreeding depression will be low if populations have the same karyotype, were isolated for <500 years and are adapted to similar environments (Frankham et al., 2011).
4. If the risk of outbreeding depression is low, then up to 20% gene flow from the source population will improve population adaptive potential; ongoing monitoring within an adaptive management framework (i.e. iterative process of decision‐making based on monitoring of outcomes) is recommended to avoid genetic swamping and outbreeding depression (Hedrick, 1995; Weeks et al., 2011; Whiteley et al., 2015).

**Figure 1:**
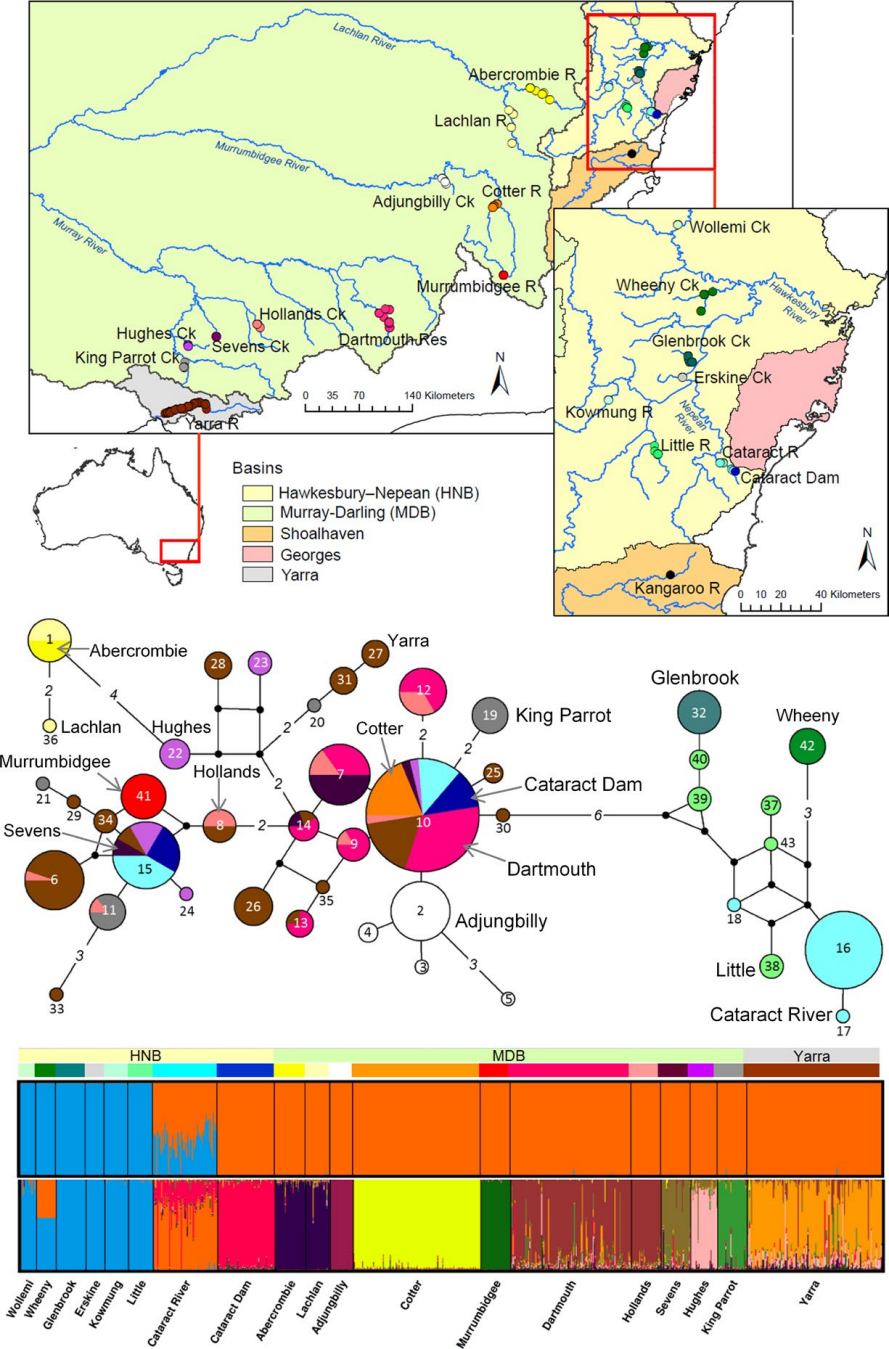
Geographic distribution of Macquarie perch samples analysed for this study (top), mitochondrial control region haplotype network showing distribution of haplotypes across populations (middle; Shoalhaven haplotype not included) and individual memberships in two or 12 genetic clusters inferred from microsatellite data (major structure inferred by *K* = 2 and *K* = 12 structure analysis of all HNB and MDB samples; see Appendix S9; Shoalhaven individual not included). Two colour stripes above structure plots are colour codes for basins (top) and populations (bottom), as on map. Yarra and Cataract Dam populations are translocated from the MDB; Cataract River comprises hybrids between endemic HNB lineage and fish dispersed from Cataract Dam (details in Appendix S9). On the haplotype network, populations are coloured as on the map (population labels are also given for each colour); each circle is a unique haplotype (number within‐haplotype ID, small black circle – missing haplotype); the size of each circle is proportional to the haplotype frequency; connections between circles are single substitutions unless marked with a number (of substitutions). In five locations, a single haplotype is fixed (1 in Abercrombie, 10 in Cotter, 41 in Murrumbidgee, 32 in Glenbrook, 42 in Wheeny)

The Macquarie perch *Macquaria australasica* Cuvier 1830 is an endangered Australian endemic freshwater fish species that has undergone substantial population decline and range contraction and fragmentation. Prior to European colonization in the late 18th century, the Macquarie perch was widespread, occurring in the inland Murray–Darling Basin (MDB), and across three coastal drainage basins, Hawkesbury–Nepean (HNB), Shoalhaven and Georges (Figure 1). Although distinct taxa within *Macquaria australasica* are not formally recognized, morphological, allozyme and DNA data all indicate that the inland and coastal forms of Macquarie perch diverged in the Pleistocene and probably represent different species (Dufty, 1986; Faulks, Gilligan, & Beheregaray, 2010a, 2015; Faulks et al., 2011; Knight & Bruce, 2010; Lintermans & Ebner, 2010; Pavlova et al., 2017). Over the last two centuries, Macquarie perch suffered strong declines, particularly in the lowland reaches of rivers, and as a result the species is now restricted to isolated locations in the headwaters of Lachlan, Murrumbidgee and Murray tributaries in the MDB, Hawkesbury and Nepean rivers in the HNB, and Georges River in the Georges Basin (Figure 1) (Gilligan, McGarry, & Carter, 2010; Knight & Bruce, 2010; Lintermans, 2007). A highly differentiated form in the Shoalhaven Basin is thought to have become extinct by 1998, which underscores the urgent need to understand and manage remaining genomic diversity within the species (Faulks et al., 2010a). Many anthropogenic factors have been implicated in the species’ decline across its range, including loss of 95% of habitat, fragmentation by barriers to movement, increased river sedimentation, alien fish species, overexploitation and altered flow regimes reducing previously connected habitats to small fragments (Cadwallader, 1978; Ingram, Douglas, & Lintermans, 2000; Ingram et al., 1990; Pollard, Ingram, Harris, & Reynolds, 1990). Loss of physical and genetic connectivity across the species’ range is expected to have contributed to its ongoing decline, although establishing that directly is challenging given the lack of historical genetic samples.

In attempts to reverse its decline, the Macquarie perch has been subject to various management actions including habitat restoration, threat amelioration, wild‐to‐wild translocations and captive breeding with subsequent stocking of captive‐bred fish to re‐establish extinct populations, augment threatened ones and support recreational fisheries (Ho & Ingram, 2012; Lintermans, 2012; Lintermans, Lyon, Hammer, Ellis, & Ebner, 2015). Despite substantial financial investment and effort, these management actions have so far failed to reverse the decline of the species. Indeed, water scarcity under recent droughts has made several of the remaining populations exceedingly small and vulnerable to environmental, demographic and genetic stochasticity. Historically, vulnerable or extinct populations would have been recolonized from neighbouring populations when flow returned, but this is no longer possible due to insufficient physical connectivity between remnant habitat patches. As others have stated (Love Stowell et al., 2017; Whiteley et al., 2015), we argue that although the main reasons for population decline (e.g. habitat availability and degradation) should be addressed by conservation management, boosting genetic diversity through assisted gene flow might be necessary to improve population fitness and/or adaptive potential and decrease extinction probability of small populations. Management decisions need to be made now based on the best available information. When direct information on genetic problems in a specific case is unavailable, drawing on accumulated evidence from empirical, theoretical and simulation studies of other systems is a more evidence‐based approach than making the unvalidated assumption that there are no genetic problems (Frankham et al., in press; Love Stowell et al., 2017; Weeks et al., 2016).

Here, we explore patterns of genetic diversity and genetic differentiation across the entire range of the Macquarie perch and expand on earlier genetic work that revealed deep divisions within the named species, hybridization among inland and coastal forms, strong population substructure and environmental factors affecting the distribution of population genetic diversity (details in Appendix S1; Faulks et al., 2010a, 2011; Pavlova et al., 2017). For the first time, we estimate effective population sizes across the species range, evaluate them against established thresholds (Frankham et al., 2014) and demonstrate by simulations that within a few decades, smaller Macquarie perch populations are likely to suffer erosion of genetic diversity and inbreeding unless genetic diversity is restored and maintained by translocations. In addition, we identify novel environmental variables associated with individual‐based heterozygosity and improve earlier estimates of population history, key divergence times, population genetic diversity and differentiation, and population structure at basin‐ and catchment scales by analysing additional samples and populations, additional microsatellite loci and longer mitochondrial sequences. Our results will contribute to developing separate management plans for appropriate evolutionary units, the selection of broodstock source populations for the current hatchery breeding programmes and the identification of critical locations for stocking and translocations. The processes of habitat loss, degradation and fragmentation that have affected the previously common and widespread Macquarie perch are similar to those affecting many species including, but not limited to, other threatened MDB species that have experienced recent population extinctions, for example trout cod *Maccullochella macquariensis,* southern pygmy perch *Nannoperca australis* and southern‐purple spotted gudgeon *Mogurnda adspersa* (Hammer et al., 2015; Lintermans, 2007; Trueman, 2011); hence, the workflow here could be readily applicable to genetic management of other threatened species to improve their adaptive potential.

## 2 Materials and methods

### 2.1 Population sampling, tissue sampling and DNA extraction

Macquarie perch samples were collected predominantly between 2002 and 2014 from 20 populations (Figure 1, Appendix S2), including ten MDB, seven HNB (note that the Cataract River population consists of endemic HNB as well as introduced MDB fish), two coastal populations that have originated from translocation of MDB individuals (Cataract Dam in HNB and the Yarra River in the Yarra River Basin) and a Shoalhaven Basin population (a single known genetic sample from Kangaroo River). Individuals were captured using various electrofishing, netting or angling techniques. Most fish were measured for length and weight and immediately released at the site of capture after collection of fin clips. Fin tissue was preserved in 100% ethanol and stored at −20°C. DNA was extracted using a qiagen DNeasy tissue extraction kit, unless available from other projects (Faulks et al., 2010a, 2011; Nguyen, Ingram, Lyon, Guthridge, & Kearns, 2012).

### 2.2 Mitochondrial control region sequencing

Sequences of an 844‐bp mitochondrial DNA fragment including the complete control region were analysed for 339 individuals from 17 populations (Appendix S2 Table S2A). For five samples, sequences were extracted from complete mitogenomes (GenBank accessions KR152235, KR152240, KR152241, KR152248 and KR152253) (Pavlova et al., 2017). For the remaining samples, mtDNA fragments were amplified and sequenced using primers A (Lee, Conroy, Howell, & Kocher, 1995) and Dmod (GCCCATCTTAACATCTTCAGTG) (Appendix S3). Chromatograms were edited and aligned in geneious Pro 6.1.3 (Drummond, Ashton, et al., 2012). Sequences were uploaded to GenBank (accession KT626048–KT626381).

### 2.3 Microsatellite genotyping

Individuals were genotyped for 19 microsatellite loci (Appendix S3). Twenty individuals with scores missing for more than three loci were excluded, as well as a single Dartmouth individual that returned a triploid genotype (confirmed by repeated genotyping of re‐extracted DNA). Final analyses included genotyping scores for 871 individuals from 20 populations (Appendix S2; Supporting Information); only 0.3% of scores were missing. Tests for deviation from Hardy–Weinberg and linkage equilibria were performed using genepop 4.2 (Rousset, 2008) (Appendix S3).

### 2.4 Estimating population history and key divergence times

To evaluate the level of mitochondrial haplotype‐sharing across populations, indicative of past gene flow, a median‐joining network (Bandelt, Forster, & Roehl, 1999) was constructed in network v4.6.1.0 (www.fluxus-engineering.com). Times of divergence among major mitochondrial lineages were previously estimated from complete mitogenomes (Pavlova et al., 2017; summarized in Appendix S1); however, some lineages were not represented. Using mitochondrial haplotypes, we estimated the times of lineage divergence with beast 1.8.2 (Drummond, Suchard, Xie, & Rambaut, 2012), assuming the HKY+G+I substitution model, coalescent tree prior and Bayesian skyline (with 10 groups and piecewise‐constant skyline model) as population size prior. A strict molecular clock was used, based on results of complete mitogenome analysis (Pavlova et al., 2017); the control region clock rate, estimated based on a wide clock rate prior in that analysis, was used here as a prior (i.e. lognormal clock rate with mean (in real space) = 0.05 and *SD* = 0.5; this corresponds to 95% of rates being sampled between 0.017 and 0.118 substitutions per site per million years). Three replicates were run for 100 million steps sampling every 100,000; runs were checked for convergence, combined and analysed in tracer 1.6.0 (Rambaut & Drummond, 2007) after discarding first 10% of samples from each run as burn‐in.

### 2.5 Genetic differentiation and tests for evolutionary timescale of divergence

arlequin 3.5 (Excoffier & Lischer, 2010) was used to calculate pairwise population *F*_ST_, *R*_ST_ (microsatellites) and Φ_ST_ (mtDNA sequences) between catchments. SPAGeDi ver 1.5 (Hardy & Vekemans, 2002) with 10,000 permutations of microsatellite allele sizes was used to test if populations have been evolving independently long enough to evolve new microsatellite alleles through mutation; locus AB009 was excluded, because it was monomorphic in all but one population.

### 2.6 Assessing population structure at basin‐ and catchment scales

structure (Pritchard, Stephens, & Donnelly, 2000) was used to identify genetic clusters from microsatellite genotypes (data on population of origin were not applied). We first tested for general population structure across the species range using all 870 MDB and HNB individuals. Twenty replicates of 10^6^ burn‐in iterations followed by 3 × 10^6^ iterations were run for each *K* from 1 to 16 (the maximum was set according to preliminary runs showing no meaningful structure above *K* = 12) using the admixture model with correlated allele frequencies. Runs were summarized using the web server clumpak (Kopelman, Mayzel, Jakobsson, Rosenberg, & Mayrose, 2015). The largest number of genetic clusters resulting in coherent geographic groups was considered to best represent population structure.

Three subsets of the data were further analysed using structure: (i) a data set of 671 individuals of MDB origin (10 MDB populations, Cataract Dam and Yarra) was run for *K*_max_ = 16 to detect major population subdivisions within MDB; (ii) a data set of 375 individuals from the Murray and Yarra catchments (Dartmouth, Hollands, Sevens, Hughes, King Parrot and Yarra populations) was run for *K*_max_ = 7 to explore the origin of two previously detected genetic clusters in Dartmouth and Yarra (Nguyen et al., 2012); and (iii) a data set of 134 individuals from six HNB populations (all except the introduced Cataract River and Cataract Dam samples) was run for *K*_max_ = 7 to test for presence of structure within the HNB.

### 2.7 Analyses of genetic diversity, proxy for historical effective population sizes

For mtDNA sequencing data, arlequin was used to calculate haplotype diversity (Hd), nucleotide diversity (π), the number of segregating sites (S) as well as Tajima's *D* (Tajima, 1996) and Fu's *Fs* (Fu, 1997) tests for selective neutrality or population size changes. For microsatellite data, arlequin was used to calculate expected heterozygosity (He); genalex (Peakall and Smouse 2006), the number of private alleles (alleles restricted to a single location); and fstat (Goudet, 2001), allelic richness (AR) standardized to the minimum sample size of 14 individuals. He and AR reflect coalescent effective population sizes (*N*_e_) under mutation–drift equilibrium and may not reliably estimate contemporary *N*_e_ associated with inbreeding and the probability of population persistence (Hare et al., 2011).

### 2.8 Analyses of contemporary effective population sizes

We estimated *N*_e_ using two single‐sample methods, both of which assume that population size is stable over a few generations. First, we used the approximate Bayesian computation method based on eight summary statistics (including linkage disequilibrium, LD) implemented in onesamp (Tallmon, Koyuk, Luikart, & Beaumont, 2008); 50,000 populations were simulated using a prior *N*_e_ range of 4–1,000 individuals. onesamp was also run with a prior *N*_e_ range of 4–500, to confirm estimate consistency. Second, an LD‐based method was used (LDNe; Waples & Do, 2008), implemented in neestimator V2.0 (Do et al., 2014); random mating model and *P*_Crit_ = 0.02 were applied. LDNe estimates were interpreted only for samples of ≥50 individuals, as smaller samples are not informative when true *N*_e_ > 100 (Tallmon et al., 2010). When estimated from a single cohort of an iteroparous species, an LD‐based estimate of *N*_e_ reflects the harmonic mean of the effective number of breeders in one reproductive cycle (*N*_b_) and effective size per generation (*N*_e_), but when estimated from mixed‐age sample, it approximates effective size per generation, albeit consistently downwardly biased (50–90% of true *N*_e_, least biased when the number of cohorts in a sample is close to the length of the generation time) (Waples, Antao, & Luikart, 2014). Whereas *N*_b_ reflects short‐term effective population size relevant to inbreeding, *N*_e_ is responsible for shaping long‐term evolutionary processes (Waples et al., 2014); thus, both would be required for evaluating two parts of the 100/1,000 *N*_e_ threshold (Frankham et al., 2014). Using simulations, Waples, Luikart, Faulkner, and Tallmon (2013) showed that the *N*_b_/*N*_e_ ratio can be approximated using two life‐history traits. According to their formulae, for Macquarie perch *N*_b_/*N*_e_ = 1.156 (assuming age at maturity of 3 years and adult life span of 23 years (Appleford, Anderson, & Gooley, 1998; Lintermans & Ebner, 2010)); thus, an LD‐based estimate of *N*_e_ should reasonably approximate both *N*_b_ and *N*_e_, provided that other biases can be accounted for. Macquarie perch samples including ≥7 cohorts would be least biased, assuming a generation time of 7 years (calculated in vortex (Lacy, Miller, & Traylor‐Holzer, 2015), below). According to the range of individual fish lengths in each population (Supporting Information) and the modelled relationship between length and age for Macquarie perch (Figure 3 of Todd & Lintermans, 2015), our Macquarie perch population samples included from ~3 to >7 cohorts, and we expect the least downward bias in LD‐based estimates of *N*_e_ for the seven populations with a range of sampled lengths >200 mm (Abercrombie, Adjungbilly, Dartmouth, Hughes, King Parrot, Lachlan, Sevens, Yarra). For species with *N*_b_/*N*_e_ ~1, LD‐based estimates of *N*_b_ (and *N*_e_) are expected to be unaffected by two‐loci Wahlund effect arising when parents from different cohorts are combined in a single sample (Waples et al., 2014).

### 2.9 Identifying environmental variables associated with individual‐based genetic diversity

We used an individual‐based Bayesian modelling approach to identify environmental variables that were correlated with spatial distribution of genetic variation (Harrisson, Yen, et al., 2016). The model was based on the 16 populations from the MDB and the HNB (translocated Yarra and Cataract Dam and admixed Cataract River samples were excluded).

The response variable was homozygosity‐by‐locus (HL), calculated for each individual using the Rhh package (Alho, Valimaki, & Merilä, 2010) in R 3.2.0 (R Development Core Team 2014). HL measures the proportion of homozygous loci per individual, attributing greater weight to homozygosity at more variable loci (Aparicio, Ortego, & Cordero, 2006). Low HL of an individual indicates low genetic similarity of its parents, which is expected in populations with high genetic diversity. Using an individual‐based measure of genetic diversity, rather than a population‐based one (such as AR), enabled us to consider environmental variation within populations (Appendix S4) rather than population averages, which should resolve environmental‐genetic associations at finer spatial scales.

Eleven environmental variables reflecting flow regime, connectivity, habitat and climate, previously shown to be indicators of Macquarie perch presence and population health (Appendix S4), were originally considered for inclusion in the model as predictors of individual genetic diversity (Table 1). All but one were sourced from the National Environmental Stream Attributes Database v1.1.5 (http://www.ga.gov.au/metadata-gateway/metadata/record/gcat_75066) (Geoscience Australia 2012; Stein, 2006) linked via stream segment number to the Geofabric stream network layer (Bureau of Meterology 2012); the data on mean November temperature were downloaded from http://www.bom.gov.au. Environmental data were extracted using ArcGIS 10.2 (Environmental Systems Research Institute 1999‐2014) for each sampling site (1–8 sites per population for 16 MDB and HNB populations; Supporting Information). Environmental predictor variables were standardized to zero mean and unit variance, and Pearson's correlations among variables were calculated (using a single record per sampling site). Only seven variables that were not highly correlated (Pearson's |*r*|<0.7) were included in the final model (Table 1; Appendices S4 and S5).

**Table 1:** Details of the environmental model predicting homozygosity‐by‐locus (HL, an inverse of genetic diversity): final set of seven variables (first column) and variables highly correlated to them (second column), predicted relationships with HL, probability of inclusion in the model (value >0.7 indicate a strong association with HL, shown in bold) and direction of the effect (positive means that a variable increases HL; negative means that a variable decreases HL). Environmental variables were sourced from National Environmental Stream Attributes Database. Environs are valley bottoms associated with the stream

Environmental predictors	Correlated variables	Predicted relationships with HL	Probability of inclusion	Direction of effect
Flow regime disturbance index calculated for period 1970–2000	Annual mean accumulated soil water surplus, Stream and environs average hottest month maximum temperature, Coefficient of variation of monthly totals of accumulated soil water surplus	Higher genetic diversity (lower HL) within larger and more permanent streams and/or more variance in genetic diversity in small rivers	0.28	Neutral
Barrier‐free flow‐path length		Higher genetic diversity (lower HL) in larger river fragments	**1**	Positive
Maximum barrier‐free flow‐path length upstream	Annual mean accumulated soil water surplus	Increase in genetic diversity (decrease in HL) with distance to upstream dam	**0.89**	Negative
Stream segment slope		Higher genetic diversity (lower HL) in streams with higher slope	0.49	Neutral
Stream and valley percentage extant woodland and forest cover		Higher genetic diversity (lower HL) in streams with more vegetated banks	0.19	Positive
Stream and environs average coldest month minimum temperature	Mean segment elevation	Lower genetic diversity (higher HL) in warmer streams	**0.73**	Positive
Mean November temperature		Higher genetic diversity (lower HL) in streams with cooler temperatures during start of the breeding season	0.47	Positive

We used a hierarchical Bayesian regression model to estimate the relationships between HL and environmental variables (Appendix S5). We used a reversible‐jump Markov chain Monte Carlo (MCMC) algorithm to perform model selection. Basin and site were included as clustering variables (given exchangeable priors, equivalent to random effects in a standard mixed model) to account for spatial clustering of the samples. The model was fitted using WinBUGS 1.4 (Lunn, Best, & Whittaker, 2009; Lunn, Thomas, Best, & Spiegelhalter, 2000; Lunn, Whittaker, & Best, 2006). Outputs were managed in R 3.1.2 (R Development Core Team 2014). All parameters were assigned vague prior distributions. The primary model outputs are estimates of the probability of inclusion for each environmental variable and the fitted associations between HL and each environmental variable. The prior probability of variable inclusion was 0.5, so that posterior probabilities of inclusion >0.5 provide evidence in favour of variable inclusion. Posterior probabilities of inclusion >0.75 provide strong evidence for variable inclusion (odds ratio >3). Full model details and model code are in Appendix S5.

Fivefold cross‐validation was used to estimate the predictive capacity of the fitted model and to test whether the model was likely to be identifying true relationships. To account for possible correlation of HL among individuals from the same genetic clusters due to shared history, cross‐validation test data sets comprised nine population clusters, with approximately 120 individuals in each test data set (1–5 population clusters; Appendix S5). We used microsatellite clusters, rather than phylogenetic groups, because recent history (e.g. drift in small populations) could have a strong effect on HL, which can differ across distinct genetic clusters. Model predictions for cross‐validation were based on environmental variables only; clustering variables (basin and site) were used in model‐fitting but not to make predictions.

### 2.10 Simulating genetic rescue/genetic restoration of isolated Macquarie perch populations

To demonstrate genetic rescue and/or genetic restoration effects of translocations to small populations of Macquarie perch, we simulated the outcomes of two management scenarios (do‐nothing vs. 50 years of translocation) for two pairs of populations using an age‐structured population model in vortex 10.1.6 (Lacy & Pollak, 2014). vortex simulates effects of deterministic forces, and those of demographic, environmental and genetic stochasticity on population parameters, such as probability of survival, time to extinction, population size, allelic diversity, heterozygosity and inbreeding (approximated as homozygosity at modelled loci). Age‐specific mortality was modelled based on Todd and Lintermans (2015) (details in Appendix S6). Inbreeding depression was modelled by assuming that each founder individual has unique recessive alleles (6.29 lethal equivalents per individual, the empirically derived vortex default value). Fifty percent of inbreeding depression was assumed to be due to recessive lethal alleles: inbred offspring with two copies of the same recessive alleles die before reproduction. The translocation scenario assumed that six individuals (3 of each sex) were moved from larger to smaller populations each year for 50 years to approximate the impacts of repeated, modest‐sized translocations; designing the most cost‐effective translocation programme is beyond the scope of this paper. All translocated adults were assumed to survive translocation. Genetic results were summarized for 1 mitochondrial and 19 microsatellite markers simulated based on observed allele frequencies. The first pair of populations had initial population sizes of 3,000 and 500 (modelled using allele frequencies of Dartmouth and King Parrot, respectively), and the second pair had initial population sizes of 300 and 100 (modelled using Cataract Dam and Murrumbidgee). Five hundred forward‐in‐time simulations were run for 100 years for each scenario using parameters outlined in Appendix S6. To compensate for the inability of vortex to model large female fecundities, calculations were restricted to adults (Lacy et al., 2015).

## 3 Results

### 3.1 Population history, divergence time estimates and genetic differentiation

The mitochondrial haplotype network was consistent with historical isolation between the MDB and the HNB, historical isolation of the Lachlan catchment from the rest of the MDB, isolation of Adjungbilly—the only known remnant Macquarie perch population in the lower Murrumbidgee catchment—from other populations, and isolation among HNB populations (Figure 1). Lack of haplotype‐sharing among any HNB populations (Appendix S7) suggests that strong genetic drift operates in this basin, consistent with earlier findings from a mitogenome study (Pavlova et al., 2017). In contrast, haplotype‐sharing across Murray River tributaries (Dartmouth, Hollands, Sevens, Hughes, King Parrot) indicated historical connectivity within the southern MDB, consistent with a continuous reconstructed historical distribution in the southern MDB (Trueman, 2011). Similarly, haplotype‐sharing between Lachlan and Abercrombie (northern MDB) supported previously inferred contemporary connectivity between these two populations (Faulks et al., 2011). The translocated fish from the MDB population into Cataract Dam apparently bore two haplotypes (that were the most common in southern MDB), both of which appear to have escaped into the Cataract River, where three endemic HNB haplotypes also occur. The translocated Yarra population shared haplotypes with most populations from the southern MDB and also had several haplotypes not sampled elsewhere.

Phylogenetic analysis in beast (Appendix S8) showed estimates of lineage divergence times consistent with those from complete mitogenomes (Pavlova et al., 2017): time to the most recent common ancestor (TMRCA) of Shoalhaven and HNB+MDB lineages was estimated at 1.061 (95% HPD 0.204–2.840) million years ago, of MDB and HNB lineages (which formed clades with posterior probabilities (PP) of 0.85 and 1, respectively) at 264 (59–674) KY (thousand years) ago, of all HNB haplotypes at 90 (16–240) KY and of all MDB haplotypes at 177 (45–464) KY. The estimates of TMRCA of Lachlan+Abercrombie clade (PP = 1) at 27 (2–88) KY bring forward the previous estimate of isolation of the Lachlan catchment from the rest of the MDB based on partial control region sequence (310 KY; Faulks et al., 2010a). Historical isolation of the lower Murrumbidgee catchment (Adjungbilly) from the upper Murrumbidgee for 36 (8–100) KY (TMRCA of Adjungbilly clade; PP = 0.83) is a novel result for a previously unstudied population.

Strong population differentiation among most HNB populations, all three MDB catchments and three populations of the Murrumbidgee catchment (Adjungbilly, Cotter and Murrumbidgee) was evident from generally high pairwise population Φ_ST_ (mtDNA) and *F*_ST_ (microsatellites) values (details in Appendices S9 and S10), consistent with Faulks, Gilligan, & Beheregaray (2010b), Faulks et al., (2010a, 2011). An evolutionary timescale of isolation within the HNB was supported by strong mitochondrial divergence among HNB populations and by SPAGeDi analysis, which showed that evolution of microsatellite allele sizes has contributed to divergence among northern HNB (Wollemi and Wheeny) versus southern HNB populations (rest) and within northern HNB (as well as between the HNB and MDB), but not within the MDB (Appendix S10). Large, significant (*p *<* *0.001) microsatellite *F*_ST_ values across almost all pairwise comparisons supported contemporary isolation of populations and strong drift effects, which is a novel result.

### 3.2 Assessing population genetic structure at basin‐ and catchment scales

For all 19 MDB and HNB populations (*N *=* *870), structure analysis assuming *K* = 2 (Figure 1) was consistent with the HNB‐MDB divergence and hybridization of translocated MDB fish with endemic HNB fish in Cataract River (Faulks et al., 2011). Our analysis of a large Cataract River sample showed that individual memberships (Q) in the endemic HNB cluster ranged from 0.68 to 0.001 and were not associated with mtDNA lineage, suggesting that gene flow from Cataract Dam might be ongoing and that hybridization in Cataract River is not limited to the first generation and is not sex‐biased (Appendix S11). All other structure analyses supported predominantly geographic structure and at least partial contemporary genetic isolation of populations within basins. Analysis of all individuals (*N *=* *870) assuming *K* = 12 yielded geographically meaningful genetic clusters (Figure 1), supported by separate analyses of 12 populations of MDB origin (*N *=* *671) and 6 populations from the southern MDB (*N *=* *375). Analysis of six HNB populations (all except Cataract River and Cataract Dam; *N *=* *134) assigned all HNB populations to different genetic clusters, except a single individual in the Little River had Q > 90% in the Kowmung cluster, suggesting a rare long‐distance dispersal event or undocumented translocation (details of all analyses are in Appendix S11). structure analyses did not support the previously reported two clusters within Dartmouth and Yarra (Nguyen et al., 2012).

### 3.3 Analyses of genetic diversity and effective population sizes

**Figure 2:**
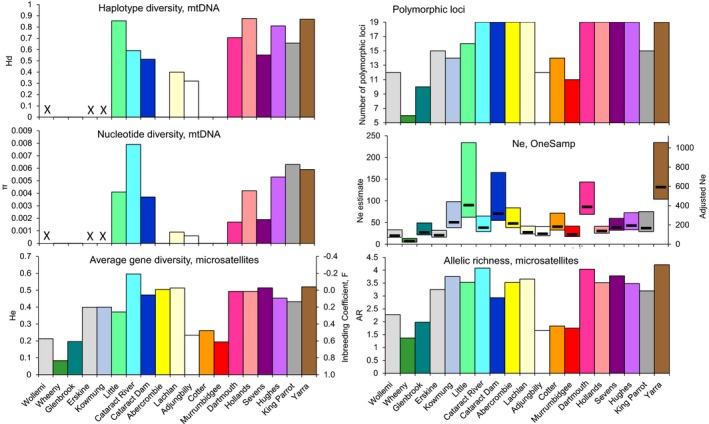
Estimates of mitochondrial and nuclear genetic diversity and effective population sizes (*N*_e_, onesamp: lower–upper 95% confidence limits, bars show mean *N*_e_) for 19 populations (coloured as on Figure 1). Crosses indicate populations for which mtDNA was not sequenced here (estimates from partial control region (Faulks et al., 2010a) were the following: Wollemi Hd = 0.111, π = 0.0006; Erskine Hd = 0, π = 0; Kowmung Hd = 0.708, π = 0.0076). Inbreeding coefficient *F*_e_ for each population is calculated as *F*_e_ = 1 − He/He_Dartmouth_; adjusted *N*_e_ is calculated as 4.5× onesamp *N*_e_ (see Section 4; lower and upper bounds are not adjusted)

Mitochondrial (Hd and π) and nuclear (AR and He) genetic diversity ranged widely across populations in both basins (Figure 2; Appendix S2). Generally, populations with low nuclear genetic diversity (Wollemi, Whenny, Glenbrook in the HNB and Adjungbilly, Cotter and Murrumbidgee in the MDB) also had low mtDNA diversity (Wollemi was not sequenced here, but had low Hd and π in the study of Faulks et al., 2010a), but mtDNA diversity was also low for some other populations (Abercrombie, Lachlan). Tajima's *D* was significantly negative for Glenbrook and Adjungbilly, suggesting purifying selection and/or past population growth. In comparison, both translocated populations (Cataract Dam and Yarra) had relatively high levels of genetic diversity (Figure 2). The Shoalhaven individual was homozygous at 17 of 19 loci.

Linkage disequilibrium‐based estimates of *N*_e_ (LDNe) for five populations with *N *>* *50 were smallest in Cotter (mean *N*_e_=63, confidence interval 35–134), slightly higher in Cataract River (164, 103–349) and Cataract Dam (128, 68–501) and moderate in Dartmouth (307, 201–593) and Yarra (344, 228–642; Appendix S2). onesamp *N*_e_ estimates for the same five populations were ~2.8× (range 1.5–4.3) lower than LDNe estimates. onesamp *N*_e_ estimates were low (mean *N*_e_ < 100) for all populations except Yarra (mean *N*_e_=132; Figure 2), consistently so for both sets of priors (Appendix S2).

### 3.4 Estimated relationships between individual genetic diversity and environmental variables

**Figure 3:**
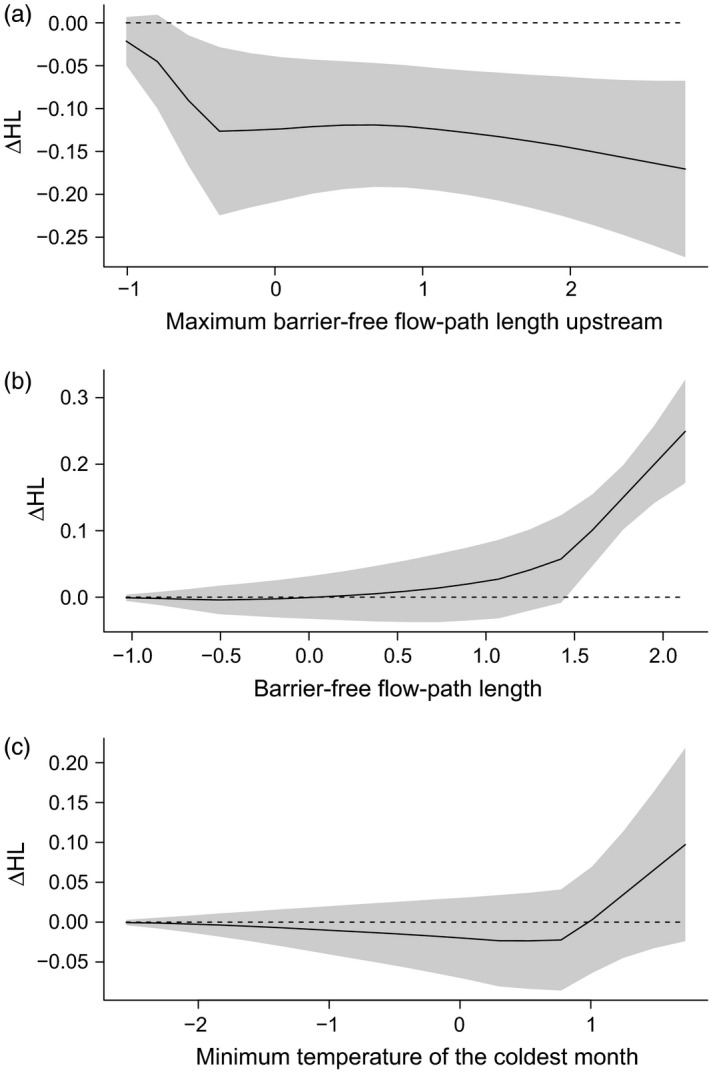
Fitted relationships between HL and environmental variables for variables with probability of inclusion greater than 0.7. The *x*‐axis shows standardized values for each variable (e.g. a value of 1 means 1 standard deviation above the mean value for that variable), and the *y*‐axis shows deviation from the mean HL value (in units of HL) for a given standardized value of the predictor variable. Grey shading is one standard deviation of the fitted effect. Models included basin and site as clustering variables, so these fitted effects account for differences among sites or between basins

Approximately 66% of the variation in HL could be explained by environmental and clustering variables (model *r* ^2^ = 0.66). Environmental variables could predict only c. 17% of the variation in HL (cross‐validated *r* ^2^ = 0.17), suggesting that stochastic processes (e.g. genetic drift in small populations) have a strong effect on genetic diversity of Macquarie perch and/or that one or more key variable impacting HL was not included in the model (e.g. EHN virus; Becker et al., 2013). Three of the seven environmental variables had probabilities of inclusion in the fitted model >0.7, indicating a strong association with HL (odds ratios > 2.3, Table 1, Appendix S5). Maximum barrier‐free flow‐path length upstream had a negative association with HL (i.e. distance from the upstream dam was associated with higher individual genetic diversity), whereas barrier‐free flow‐path length, and minimum temperature of the coldest month, had positive associations with HL (Table 1, Figure 3).

### 3.5 Simulating genetic rescue/genetic restoration of small isolated Macquarie perch populations

For do‐nothing scenarios run over 100 years, models for all four populations showed overall decrease in survival probability, decrease in genetic diversity and increase in inbreeding and inbreeding depression over time (Appendix S6). With decreasing initial population sizes (3,000, 500, 300 and 100 for Dartmouth, King Parrot, Cataract Dam and Murrumbidgee, respectively) extinction probability increased, time to the first extinction decreased, inbreeding depression increased (i.e. the number of lethal alleles per individual at year 100 decreased) and proportion of retained heterozygosity for extant populations decreased (He_year100_/He_year0_: 0.98, 0.89, 0.85 and 0.79; Table 2). Final *N*_e_–values estimated based on allele frequency changes resulted in a mean *N*_e_/*N* ratio of 0.17 (range 0.1–0.26; Table 2).

**Table 2:** Summary of Vortex simulations of two management scenarios. In 50‐years‐of‐translocation scenarios, King Parrot and Murrumbidgee populations are supplemented by individuals from Dartmouth and Cataract Dam, respectively

Scenario	Do‐nothing		50‐years‐of‐translocation		Do‐nothing		50‐years‐of‐translocation	
Populations	Dartmouth	King Parrot	Dartmouth	King Parrot	Cataract Dam	Murrumbidgee	Cataract Dam	Murrumbidgee
Initial population size, *N*	3,000	500	3,000	500	300	100	300	100
Probability of extinction	0.002	0.21	0.022	0.006	0.36	0.828	0.776	0.032
Time to first extinction, years	85	82	76	92	78	63	42	90
*N*_e_ at Year 100	340	61	257	−221	44	31	42	−18
*N* (extant) at Year 100	3,269	337	3,743	680	337	118	478	450
*N*_e_/*N*	0.104	0.181	Not estimated	Not estimated	0.131	0.26	Not estimated	Not estimated
He at year 0	0.491	0.426	0.491	0.426	0.472	0.19	0.472	0.19
He at year 100	0.481	0.379	0.478	0.442	0.401	0.151	0.398	0.331
Number of alleles at year 0	5.68	3.52	5.68	3.52	3.36	1.8	3.36	1.8
Number of alleles at year 100	5.03	2.92	4.95	4.26	2.6	1.5	2.6	2.86
Number of haplotypes at year 0	6	4	6	4	2	1	2	1
Number of haplotypes at year 100	5.53	2.53	5.36	6.11	1.73	1	1.75	2.51
Number of lethal alleles/individual at year 0	3.15	3.15	3.15	3.15	3.15	3.15	3.15	3.15
Number of lethal alleles/individual at year 100	3	2.53	2.94	2.8	2.28	1.83	2.44	2.72

**Figure 4:**
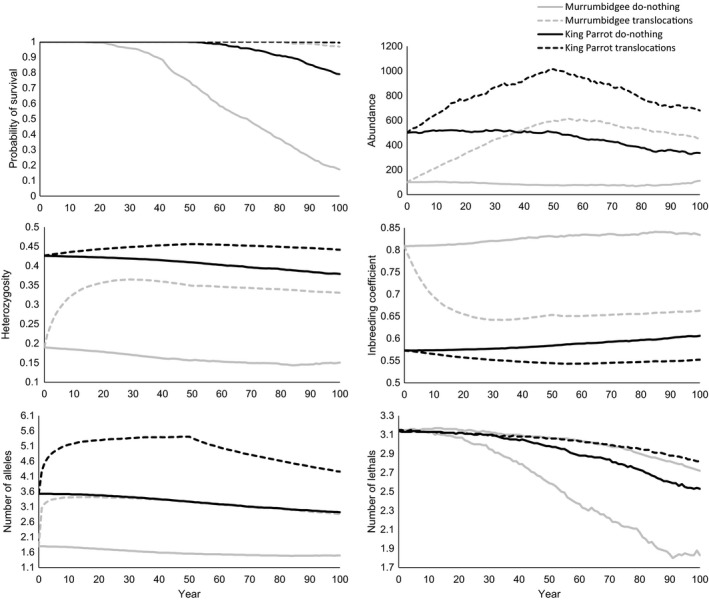
Results of Vortex simulations for King Parrot (initial population size *N *=* *500; black line) and Murrumbidgee (*N *=* *100; grey line) under do‐nothing (solid line) or 50‐years‐of‐translocation (dashed line) scenarios

Results of 50‐years‐of‐translocation scenarios showed that addition of individuals into King Parrot and Murrumbidgee, compared to do‐nothing scenarios, decreased population probability of extinction 35‐ and 26‐fold, respectively, increased time to first extinction and decreased inbreeding depression 1.1‐ and 1.5‐fold, and for populations extant at year 100, increased population sizes (2‐ and 3.8‐fold), heterozygosity (1.2‐ and 2.2‐fold) and number of alleles (2.4‐ and 2.5‐fold; Table 2, Figure 4). The genetic restoration effect was rapid: in the first 10 years of translocations, the number of alleles in King Parrot reached 90%, and in Murrumbidgee >100% of those of the source populations (Appendix S6). Genetic rescue from inbreeding depression (i.e. larger number of lethal alleles per individual under 50‐years‐of‐translocation compared to the do‐nothing scenario; Figure 2) was apparent for the small Murrumbidgee population at year 10 and for the larger King Parrot at year 40. Genetic rescue/restoration effects were long‐lasting: genetic diversity remained higher and inbreeding lower than in do‐nothing scenario even 50 years after cessation of translocations. Effect of harvest (for translocation) on population size, heterozygosity, number of alleles or inbreeding of both source populations was minimal, but harvest increased the probability of extinction and decreased mean time to extinction for the smaller Cataract Dam (and to a lesser extent, the larger population at Dartmouth; Table 2, Appendix S6).

## 4 Discussion

### 4.1 The need for specieswide genetic management to prevent further loss of genetic diversity and boost adaptive potential

Using novel genetic data, we investigated whether genetic augmentation is likely to be beneficial and necessary for long‐term survival of an endangered freshwater fish, the Macquarie perch. The species has experienced dramatic declines in range and numbers of most populations, intense habitat fragmentation, levels of inbreeding at which inbreeding depression is often observed in wild populations and continuing loss of populations despite intensive management. Assuming levels of inbreeding depression similar to those reported for other wild populations, our simulations support a decline in population viability unless there is active genetic management. We believe that these combined lines of evidence provide sufficient support for genetic augmentation to be implemented now, despite a lack of data on fitness decline, which may take too long to collect in order to be useful for preventing extinctions of many populations (Weeks et al., 2016).

Significant investment in habitat restoration and protection has failed to lead to detectable recovery of some Macquarie perch populations (Mark Turner, GBCMA pers. comm.). Although no data on fitness loss exist, it is reasonable to assume that inbreeding depression might have contributed to this failure. Our simulations suggest that inbreeding depression may contribute to population declines and impede population recovery in the near future. Our estimates of *N*_e_ suggest that most populations will have limited capacity to adapt to rapid environmental changes. Thus, genetic problems should be addressed in conjunction with other key threats to population persistence. Restoring the historical, within‐basin genetic connectivity of Macquarie perch via assisted gene flow will improve genetic diversity and adaptive potential across the species’ range. Our simulations indicate that small‐scale translocations (6 adults/year) will result in rapid and long‐lasting increase in genetic diversity (limited only by genetic diversity and divergence of the source(s)) and decrease in inbreeding and probability of population extinction. Although large source populations should not be impacted by appropriately limited harvest (e.g. for translocation or captive breeding), smaller sources may become more vulnerable to extinction, loss of genetic diversity and inbreeding. Therefore, where possible, management should consider reciprocal translocations and multiple sources.

Our vortex model has a number of limitations: (i) offspring that do not survive to reproduction were not modelled (due to software limitation), but, given extreme fecundity (up to 110,000 eggs/female; Cadwallader & Rogan, 1977), strong purifying selection acting on juveniles could purge deleterious mutations, reducing inbreeding depression; (ii) individuals at the start of simulations were assumed to be unrelated, which, if incorrect, artificially increases the time when inbreeding depression starts to impact viability; (iii) mutations were not modelled, and thus, novel genetic diversity was not accounted for; (iv) adaptive capacity was not addressed; (v) there are large uncertainties around environmental effects and population parameters, including an assumption that all individuals survive translocation. Nevertheless, our simulations are in agreement with previous simulation studies (Allendorf & Ryman, 2002; Rieman & Allendorf, 2001) that under management inaction, populations of 100–500 adults will lose genetic diversity over time (>10% of alleles and >10% of heterozygosity in 100 years), and the loss will be faster in smaller populations. Inference of low genetic diversity in Macquarie perch (mean observed heterozygosity across populations Ho = 0.371, estimated from 19 loci with 13.5 alleles/locus on average) was consistent with similar inferences for other threatened Australian freshwater fishes, Yarra pygmy perch *Nannoperca obscura* (Ho = 0.318; 14 loci, 11.9 alleles/locus; Brauer, Unmack, Hammer, Adams, & Beheregaray, 2013), southern pygmy perch *N. australis* (Ho = 0.47; 12 loci, 10.3 alleles/locus; Cole et al., 2016) and dwarf galaxias *Galaxiella pusilla* (Ho = 0.395; 11 loci, 16.3 alleles/locus; Coleman et al., 2010), although estimates of Ho themselves might not be strictly comparable when estimated from marker sets of different variability (Hedrick, 1999). Nevertheless, higher diversity reported for the golden perch *Macquaria ambigua* (Ho = 0.52; 8 loci, 15.3 alleles/locus; Faulks et al., 2010b), the more common and abundant Macquarie perch congener, is consistent with an earlier finding of a mean decrease of 35% in heterozygosity in 170 threatened taxa compared to their nonthreatened relatives (Spielman, Brook, & Frankham, 2004).

Although in simulations inbreeding depression did not appear to impact the largest population (initial *N *=* *3,000, vortex *N*_e_ = 257) for at least 50 years (~7 Macquarie perch generations), it started to impact three smaller populations (initial *N *≤* *500, vortex *N*_e_ < 100) within the first 10 years (i.e. <2 generations), supporting the short‐term inbreeding threshold *N*_e_ of 100 (Frankham et al., 2014). Inbreeding can be approximated for a small population by scaling its genetic diversity by the diversity of a known outbred population using the effective inbreeding coefficient *F*_e_: *F*_e_ = 1 − He_inbred_/He_outbred_, where He_inbred_ is heterozygosity (for neutral variation) of a population in question and He_outbred_ is heterozygosity of an outbred population (Frankham, 1998). If Dartmouth, one of the largest natural populations of Macquarie perch in the MDB, is used as an outbred reference, then *F*_e_ coefficients for Wollemi, Wheeny, Glenbrook, Adjungbilly, Cotter and upper Murrumbidgee are >0.45, and for Little is 0.25 (i.e. is as high as *F*_e_ for the offspring of full siblings; Figure 2). At *F*_e_ of 0.2, inbreeding depression is typically observed for populations of naturally outcrossing species (Frankham, 1995; Szulkin & Sheldon, 2007; Walling et al., 2011; Woodworth et al., 2002). Consistently, our simulations show that within a decade, inbreeding depression can be expected to act in Macquarie perch populations of ≤500 adults, unless translocations are performed. Although theoretically one effective migrant per generation may be sufficient to provide inbreeding connectivity (i.e. significantly reduce harmful effects of inbreeding; Mills & Allendorf, 1996), as well as adaptive connectivity (i.e. potential of highly advantageous alleles to spread in a recipient population; Rieseberg & Burke, 2001), it is not expected to drive *F*_e_ below 0.2 (Lowe & Allendorf, 2010). In any case, well before *F*_e_ climbs to 0.2, many populations will already be suffering inbreeding depression. A long‐term study of inbreeding depression in the wild using genomic estimates of inbreeding detected major negative impacts on lifetime reproductive success at *F* even below 0.1 (Huisman, Kruuk, Ellis, Clutton‐Brock, & Pemberton, 2016). Thus, more than one effective migrant per generation may be required to drop *F*_e_ values sufficiently (e.g. below 0.1) to rescue genetically depauperate populations from strong inbreeding depression (Frankham et al., in press; Weeks et al., 2011).

Contemporary *N*_e_ related to inbreeding is best estimated using two or more temporal genetic samples (inbreeding *N*_e_; Luikart, Ryman, Tallmon, Schwartz, & Allendorf, 2010), or using a sample from a single‐cohort and adjusting estimates using life‐history traits (Waples et al., 2014). *N*_e_ relevant to adaptive potential is best estimated from two or more genetic samples taken several generations apart and measuring the change in allele frequencies over time (variance *N*_e_; Luikart et al., 2010). Our data were not suitable for temporal analyses: although sampling was spread over years, temporal samples spanning generations at individual sites were lacking, but sampling was random, comprising multiple generations, and overlapped 15‐year drought, during which many populations ceased regular breeding. Because *N*_e_ and population size are key parameters for evaluating existing thresholds and predicting future population trajectories, here we used single‐sample *N*_e_ estimates corrected for biases, along with *N*_e_/*N* ratios from simulations based on the observed allele frequencies, to estimate bounds on population sizes of Macquarie perch. Although we believe that our estimates of *N*_e_ and *N* should be sufficiently accurate for the purposes stated above, they are associated with a number of untested or violated assumptions (see below) and hence should be interpreted with caution and re‐examined when genomic data become available (Hollenbeck, Portnoy, & Gold, 2016). If we assume that (i) onesamp *N*_e_ estimate is systematically downwardly biased and is ~2.8× (range 1.5–4.3) lower than LDNe *N*_e_ (based on five samples of >50 individuals with both estimates; Appendix S2), (ii) LDNe *N*_e_ estimate is ~1.6× (1.1–2) lower than the true *N*_e_ (Waples et al., 2014) and (iii) *N*_e_ is ~5.9× (3.8–9.6) lower than the number of adult Macquarie perch *N* (Table 2), then (i) the true *N*_e_ for each population could be ~4.5× (1.7–8.6) higher than onesamp *N*_e_ estimate (adjusted *N*_e_; Figure 2), and (ii) adult population size *N* could be ~44.4× (6.27–82.6) higher than onesamp *N*_e_ estimate (Appendix S2).

Given that inbreeding *N*_e_ is similar to variance *N*_e_ for this species (see Section 2), comparisons of the above approximations with 100/1,000 minimum *N*_e_ thresholds of Frankham et al. (2014) suggest that (i) the majority of populations have mean *N*_e_ < 600 (lower bound of *N*_e_ < 100, upper bound of *N*_e_ < 1,000), implying that genetic restoration is required to boost population adaptive potential and avoid inbreeding depression within a few decades; (ii) Wollemi, Wheeny and Erskine have mean *N*_e_ < 100, implying they might experience inbreeding depression within a few generations even if they do not already; (iii) the majority of populations have mean sizes <3,000 (lower bound <500, upper bound <10,000), which our simulations suggest may lead to genetic problems in the next decade; (iv) Little, Cataract Dam, Dartmouth and Yarra have the largest *N*_e_ and mean sizes >3,000 and thus are the best sources for translocations. We note that for four populations with sample sizes of 30 individuals (Upper Murrumbidgee, Hollands, Sevens and King Parrot) LDNe estimates of *N*_e_ had means and ranges <100 (e.g. outside the range of Tallmon et al. (2010)'s simulations) and were smaller than onesamp estimates, suggesting that for small populations these onesamp‐based approximations could be strongly upwardly biased (Appendix S2). For example, the number of adults monitored at the Upper Murrumbidgee site is estimated at ~20–50 (Lintermans, unpublished data), whereas our onesamp‐based approximation provides a much more optimistic value of 113–3,500 (the LDNe‐based approximation is closer, 13–307; Appendix S2).

For an ideal Wright–Fisher population of a stable size, different methods of estimating *N*_e_ (e.g. from LD, loss of genetic diversity over generation, drift among replicates over generations and/or inbreeding coefficients) should yield similar estimates. However, when conditions of the Wright–Fisher population model are violated (e.g. by the effects of unequal sex‐ratio, variance in family sizes and/or fluctuations in population size over generations), different methods yield estimates related to different time frames and spatial scales (Crow & Kimura, 1970; Wang, 2005). The calculations above do not correct for biases related to violations of the Wright–Fisher model. In declining populations, estimates of *N*_e_ are expected to be upwardly biased, reflecting a previously large *N*_e_ one to two generations earlier (for LDNe) or several generations in the case of onesamp, and declines are observed in some Macquarie perch populations (e.g. Yarra; Tonkin, unpublished data). Despite much uncertainty around our approximations of population sizes, many of our results point to the need to restore genetic diversity by translocations in most Macquarie perch populations. Reconnection of several populations by gene flow might be required to achieve a global *N*_e_ > 1,000 necessary to maintain population adaptive potential and evolvability in the face of environmental change (Weeks et al., 2011). Measuring *N*_e_ dynamics using multiple temporal genetic samples will allow managers to monitor the effects of assisted gene flow on *N*_e_. When genomic data including those on physical linkage or genomic position of genetic markers become available for Macquarie perch, contemporary *N*_e_ and its recent change could be estimated from contemporary samples (Hollenbeck et al., 2016).

### 4.2 Designing translocation strategies while considering risks of outbreeding depression and loss of local adaptation

Selecting appropriate sources of admixture requires simultaneous consideration of the timescale of population differentiation and the risks of inbreeding versus outbreeding depression and loss of local adaptation (Frankham et al., 2011; Love Stowell et al., 2017; Weeks et al., 2011, 2016). Long‐term divergence implies that reproductive isolation may have evolved through adaptive differentiation and/or drift; hence, admixture between regions showing histories of long‐term divergence and/or local adaptation should be undertaken with caution (Frankham et al., 2011). Long‐term divergence of the MDB and HNB Macquarie perch lineages (119–385 KY; Pavlova et al., 2017) in different environments (e.g. Appendix S4) might have resulted in adaptive differences between basins. However, analyses of complete mitogenomes suggest that genetic drift was the prominent force driving mitochondrial divergence; drift was stronger in the HNB either due to smaller historical effective population sizes (approximated by allelic richness or haplotype diversity) or weaker environmental pressures (Pavlova et al., 2017). In accordance with mitochondrial differentiation, the microsatellite genotype of the single individual from the Shoalhaven Basin population differed markedly from those of the HNB and MDB lineages (Appendices S8 and S9). The fact that the Shoalhaven individual was homozygous at 90% of loci suggests that loss of genetic variation and individual inbreeding accompanied extinction of the endemic Shoalhaven lineage. The widespread and not sex‐limited hybridization beyond first generation of HNB and MDB lineages in lower Cataract River provides no evidence for outbreeding depression, but evidence for absence of genomic incompatibilities and unimpeded fitness of hybrids is lacking (Verhoeven, Macel, Wolfe, & Biere, 2011). Separate management of Macquarie perch lineages from different basins, which is current practice, is warranted until an informed risk–benefit analysis suggests otherwise (Frankham et al., 2011). Likewise, long‐term (58–191 KY; Pavlova et al., 2017) divergence between the northern (here: Wollemi, Wheeny) and southern HNB populations (here: the remaining HNB populations) suggests that genetic rescue/restoration be conducted independently within these regions, unless an informed risk–benefit analysis indicates otherwise.

In some cases, nearby (presumably similarly adapted) source populations do not exist, so there may be no option but to source individuals for translocation from a more strongly divergent population (Love Stowell et al., 2017; Weeks et al., 2011), as suggested for threatened southern pygmy perch from the MDB based on genotype–environment association analyses (Brauer, Hammer, & Beheregaray, 2016). In the Lachlan catchment (MDB), two sampled Macquarie perch populations are recent derivatives from the same source (and are connected by unidirectional gene flow from the Abercrombie to Lachlan; Faulks et al., 2011); thus, little genetic rescue/restoration effect would be expected from their admixture (Frankham, 2015). However, the estimate of divergence of the Lachlan catchment from the other MDB catchments (2–88 KY ago) is not recent enough to dismiss the possibility of outbreeding depression under admixture with southern MDB populations under recent divergence criteria (Frankham et al., 2011). The range of likely adjusted *N*_e_ values (41–360) and approximate population sizes (150–3,500) for the two Lachlan catchment populations suggests that under a do‐nothing scenario these populations will face inbreeding and loss of genetic diversity within a few generations. Although knowing with certainty the fitness consequences of cross‐catchment admixtures would be desirable, the need for management intervention to prevent loss of genetic diversity and inbreeding in the near future may necessitate gene flow from larger populations of fish of MDB genetic origins (e.g. Dartmouth or Yarra) before the risk of outbreeding depression can be assessed through breeding experiments (Frankham, 2015; Frankham et al., in press). This suggestion is in line with calls for focussing on preservation of genetic diversity at the species level, rather than population, subspecies or evolutionary significant unit (Love Stowell et al., 2017; Weeks et al., 2016).

In cases when it is unclear whether mitochondrial divergence confers selective advantage on local mitogenomes (such as Adjungbilly, inferred to have diverged from the other populations of the Murrumbidgee catchment ~8–100 KY ago), genetic rescue could involve within‐catchment translocation of males. Assuming that on this timescale mitochondrial divergence did not drive mito‐nuclear co‐evolution (Wolff, Ladoukakis, Enriquez, & Dowling, 2014), this translocation approach would preserve any potential selective advantage of local mtDNA while providing nuclear gene flow for increasing *N*_e_ (Weeks & Corrigan, 2011).

In contrast to cases of long‐term divergence, admixture of populations that have diverged only recently (including due to recent anthropogenic barriers to movement) should be associated with very low risk of outbreeding depression or loss of local adaptation (Frankham, 2015), whereas managing these populations separately will decrease their adaptive potential and increase species extinction risk (Coleman et al., 2013; Weeks et al., 2016). In the HNB, extreme divergence between the two northern Macquarie perch populations (Wollemi and Wheeny) is likely due to recent isolation by anthropogenic barriers (Faulks et al., 2011) and strong effects of genetic drift in very small populations (mean adjusted *N*_e_ < 100). In the MDB, historical records indicate that prior to arrival of Europeans in Australia, Macquarie perch were common along the length of the Murrumbidgee River and in many tributaries of the Murray River (including lowland zone of the Central Murray River), suggesting that the Murrumbidgee and Murray catchments were previously connected by gene flow (Trueman, 2011). Historical cross‐catchment connectivity is supported by the most common MDB haplotype (10 on Figure 1) being shared between Murrumbidgee and Murray catchments. Current differentiation among populations within the Murrumbidgee and Murray catchments (likely resulting from very recent isolating effects of large dams and impoundments) should be reversed by within‐catchment admixtures. Occasional cross‐catchment translocations should also be considered to improve population adaptive potential. Designing cross‐catchment translocations (e.g. from the Murray catchment to the Lachlan or Murrumbidgee catchments) would benefit from further modelling and captive breeding experiments measuring relative fitness of intercatchment crosses. Similar recommendations were suggested for other threatened Australian fishes with anthropogenically impeded genetic connectivity (Brauer et al., 2013, 2016; Cole et al., 2016).

Historically translocated populations, that have been isolated for sufficient generations to accumulate allelic differentiation, represent viable sources for translocations back into their population of origin if they contain diversity lost from the recipient population (Frankham, 2015). Two translocated populations of Macquarie perch, Cataract Dam, established from the Murrumbidgee River in 1916 (Legislative Assembly of New South Wales 1916) and the Yarra, sourced between 1907 and 1943 from multiple southern MDB populations including King Parrot Creek, Broken River and the Goulburn River (where the species is now locally extinct; Cadwallader, 1981; Trueman, 2011; Ho & Ingram, 2012), could be used as source populations for low‐risk translocations to Murrumbidgee and Murray catchments, respectively. Given their high genetic diversity, Cataract Dam and Yarra populations may constitute particularly viable translocation options, provided their *N*_e_ and *N* can support it.

Finally, our simulations suggest that in cases when conditions of a remnant population are unlikely to improve enough to facilitate long‐term sustainability (as in creeks lacking connectivity with more stable river systems, and with insufficient water availability under climate change scenarios), translocating all remaining individuals into a more sustainable system might be more beneficial than attempting to maintain the original populations, for the overall chance of species survival and adaptive potential. Successful examples of fish rescue during extreme drought and bushfire runoff already exist (including Macquarie perch rescue; Kearns, 2009; Hammer et al., 2013, 2015). Management plans incorporating such actions could have prevented high mortality and loss of genetic diversity of the Broken River population of Macquarie perch during the Millennium Drought of 1995–2009; only three individuals have been detected in the reach since 2011, whereas prior to the drought this population was considered stable (Kearns, unpublished data).

### 4.3 Environmental correlates of genetic diversity

Environmental modelling suggested that Macquarie perch were more genetically diverse (i.e. had lower HL) if they were (i) further downstream from a barrier (dam wall, spillway or large dam) or had larger flow‐path lengths upstream, (ii) in a short‐to‐medium, but not long, stretch of a barrier‐free fragment of the stream, and (iii) in streams that are colder in winter. Lower genetic diversity in sites immediately downstream from dams could indicate that cold‐water pollution and/or other aspects of water supply releases (e.g. changes in habitat quality/availability) have a long‐lasting effect on Macquarie perch populations, as was previously inferred for Murray cod (Todd, Ryan, Nicol, & Bearlin, 2005). Indeed, the Macquarie perch population in the Mitta Mitta River downstream of Dartmouth Dam in Victoria was extirpated following the dam's construction, with cold‐water pollution the most likely cause (Koehn, Doeg, Harrington, & Milledge, 1995). Alternatively, the correlation between barrier‐free flow‐path length upstream and mean annual flow (*r* = 0.78, Appendix S5) suggests that larger streams (with higher mean annual flow) might be harbouring larger populations, for example by providing more habitat and/or by being more resilient to droughts or other disturbances. Unfortunately, few reaches with these attributes remain, with the overwhelming majority of mid‐ and upland reaches of large rivers where the species were once abundant (Trueman, 2011) now containing, or regulated heavily by, major weirs and dams. High levels of correlation among environmental variables make it challenging to identify underlying causal drivers.

Our second result, that individuals are less diverse in very long connected stream fragments (>1.5 standard deviations from the mean length; Figure 3), is counterintuitive, as one would expect longer barrier‐free paths to provide more habitat. The longest barrier‐free flow‐path lengths are restricted to three MDB populations: Adjungbilly, upper Murrumbidgee and Sevens (Appendix S4), and are largest (>2 standard deviations) in Adjungbilly and upper Murrumbidgee, the two populations with low genetic diversity. Thus, a significant relationship between barrier‐free flow‐path length with HL could be spurious and driven by population history or other correlated variables (e.g. limited access to suitable spawning sites or presence of natural barriers that subdivide population – that is, waterfalls, not considered for this metric). The final finding that individuals are less diverse in streams that are warmer in winter raises the possibility that some life stages of Macquarie perch could be sensitive to warmer winters and that climate warming may further exacerbate loss of genetic diversity. Although we found some potentially meaningful environmental correlates of individual genetic diversity, the data were limited in their ability to distinguish whether environmental factors or population history (genetic drift/gene flow/mutation) are the main determinants of genetic diversity, mainly because the species is limited to very few small and isolated upstream reaches, without much environmental variation across sites in each (Appendix S4).

### 4.4 Summary of management recommendations

We found that most Macquarie perch populations are associated with low levels of genetic diversity and effective population size. Given that Macquarie perch continue to experience ongoing declines in spite of habitat restoration and threat amelioration efforts, these populations are at risk of extinction, at least in part due to negative genetic effects (e.g. inbreeding depression, low adaptive potential). To elevate genetic diversity, *N*_e_ and population sizes, we recommend reconnecting populations *within* the following regions with assisted gene flow: (i) northern HNB; (ii) southern HNB; (iii) the Murray catchment, where Yarra is recommended as an additional source of translocations; and (iv) the Murrumbidgee catchment, where Cataract Dam is recommended as an additional source. Several lines of evidence suggest that the mixing recommended above poses a negligible risk of outbreeding depression and swamping of local adaptation: (i) many populations are derived from a larger ancestral population with genetic diversity primarily lost through drift in populations that are small through human actions; (ii) these populations have only recently been isolated; thus, genomic incompatibilities are unlikely; (iii) the admixed Yarra population appears to be healthy and self‐sustainable. Managing populations within these regions as a metapopulation is likely to be beneficial for long‐term population viability.

Genetic rescue/restoration is a powerful management tool for a number of reasons: (i) it can have a large and long‐lasting impact on genetic diversity and adaptive potential for relatively little additional work and expense (Frankham, 2015), (ii) it does not necessarily require ongoing intervention, although if populations remain small, assisted gene flow will need to be performed periodically (Hedrick, Peterson, Vucetich, Adams, & Vucetich, 2014), (iii) it could work at a range of scales from single species rescue (Hedrick & Fredrickson, 2010) to landscape reconnections (Smith, van der Ree, & Rosell, 2015). Population viability analysis can assist in developing objective and measurable criteria of metapopulation connectivity (Carroll, Fredrickson, & Lacy, 2014; Lacy, 2000), provided sufficient knowledge of species biology exists. Empirical results from other systems could be used to help provide priors for unknown parameters (e.g. inbreeding depression) in population viability models used to predict population persistence under management inaction and various scenarios of genetic intervention. Translocations of juveniles, in addition to adults, over several consecutive years have been advocated when there are limited adult fish available in a source populations (Todd & Lintermans, 2015). For these cases, we argue for translocating genetically diverse sets of individuals, achieved by collecting a small number of juveniles from each of many sampling sites. Numbers of translocated juveniles would need to account for high juvenile mortality (e.g. for Macquarie perch ~56 1‐year‐old juveniles would be required to produce 6 adults; Appendix S6; Todd and Lintermans (2015)).

Even if direct evidence of genetic problems (fitness loss) in specific cases is regarded as desirable, it should not be required to justify genetic intervention for small populations. Genetic augmentation alone often may not be sufficient to reverse population declines, but it must accompany other threat management actions for small populations (Frankham et al., in press; Love Stowell et al., 2017). Performed and monitored carefully within a risk‐assessment framework (e.g. after considering the risk of outbreeding depression), in most instances genetic augmentation will be beneficial, and not harmful, for long‐term population viability.

## Data Archiving Statement

Data for this study are available as Appendices and Supporting Information (collection details, lengths, microsatellite genotypes, mitochondrial control region sequences and HL values for 872 Macquarie perch samples from 20 populations and 11 raw and standardized environmental variables for 16 populations). Sequence data are also deposited to GenBank (accession KT626048–KT626381).
