# Supplementary material for: Severe consequences of habitat fragmentation on genetic diversity of an endangered Australian freshwater fish: A call for assisted gene flow
Source: Evol Appl. 2017 May 11;10(6):531–50. doi: 10.1111/eva.12484 (PMC5469170; doi:10.1111/eva.12484)
Supplement: Supplementary file 1 [file EVA-10-531-s001.docx]

Appendix 1. Summary of previous Macquarie perch population genetic research relevant to planning genetic management.

The Macquarie perch has been subject to broad-scale population genetic analyses that have revealed deep divisions within the named species, hybridization among inland and coastal forms, strong population substructure and environmental factors affecting the distribution of genetic variation. Because previous studies and management actions inform hypotheses, decisions about sampling protocols and molecular techniques, we provide a brief summary of the main points below.

*Incomplete knowledge of timing of population divergence*

Previous mitochondrial phylogeographic analyses consistently show Macquarie perch to be strongly subdivided at the basin-scale. From analysis of mitogenomes, the recently extinct Shoalhaven lineage was estimated to have diverged from the ancestor of the inland MDB and coastal HNB lineages 419-1,332 KY (thousand years) ago, which was followed by divergence between MDB and HNB lineages 119-385 KY ago, and later by divergence of northern HNB sublineage (represented here by Wollemi and Wheeny populations; Fig. 1 of the main text) and southern HNB sublineage (here: remaining HNB populations) 58-191 KY ago (Pavlova et al. accepted). Faulks et al. (2010) also estimated within-MDB divergence (Lachlan catchment from the rest) of ~310 KY ago, based on fragment of mitochondrial control region. Population genetic analyses based on nuclear microsatellites show further within-basin subdivision. Microsatellite analysis of 8 loci identified four major population groups: northern and southern in the HNB, and Murrumbidgee and Lachlan + Murray in the MDB (Faulks et al. 2011). Based on combined nuclear and mitochondrial evidence, two HNB (northern, southern) and three MDB population groups (Murrumbidgee, Lachlan and Murray) were recognized (Faulks et al. 2011). Because inferences about the distinctiveness of the Lachlan population were inconsistent between analyses based on nuclear and mitochondrial data, we analysed timing and process of population divergence using additional samples (including populations not previously analysed) and microsatellite loci (including loci cloned from Macquarie perch not available at the time of the earlier study), and complete control region sequences (incorporating substitution rate uncertainty).

*Hybridization between diverged mitochondrial lineages*

Controlled breeding experiments designed to reveal functional consequences of hybridization between lineages have not been conducted. However, an inadvertent experimental test of admixture between inland and coastal Macquarie perch presented itself when fish historically translocated from the MDB to Cataract Dam (HNB) have escaped downstream into Cataract River, where they hybridize with the endemic HNB population (Faulks et al. 2011). Divergence of mitochondrial genomes between the MDB and HNB (Pavlova et al. accepted) might have resulted in co-evolution of mito-nuclear gene complexes that could be disrupted by hybridization leading to outbreeding depression (Rand et al. 2004; Verhoeven et al. 2011). Here we assess the extent and direction of hybridization in Cataract River and test whether nuclear gene flow is impeded between mitochondrial lineages in the Cataract River.

*Understanding relationships between environmental variables and genetic diversity*

All population genetic studies of Macquarie perch have indicated population substructure. If substructure is a consequence of local adaptation and different evolutionary histories, conservation management should weigh up the costs and benefits of maintaining it, whereas if they are due to human impacts, management should try to reverse it with gene flow (Frankham et al. 2011). Riverscape genetics has provided useful insights by elucidating the landscape and environmental features that promote population genetic diversity and differentiation among populations (Faulks et al. 2011). In the HNB, genetic differentiation increased with riverine distance and the number of anthropogenic barriers between sites (Faulks et al. 2011). Genetic diversity positively correlated with river slope, a proxy for the abundance of riffles required for Macquarie perch breeding. However, slope might not be the optimal proxy for riffles, and not all riffles are suitable spawning habitat (Tonkin et al. 2015). Since the publication of Faulks et al. (2011), environmental data availability has improved dramatically, and hence we undertake a comprehensive species-wide analysis of environmental determinants of population health.

*Understanding the meaning of subtle within-population structure*

A study based on 8 microsatellite loci in the state of Victoria (southern MDB) found two nuclear genetic clusters present in Yarra and Dartmouth, but only a single cluster in the other populations (Hollands, Hughes and Buffalo River) (Nguyen et al. 2012). The same two clusters were not found in different samples from Yarra and Dartmouth applying a different set of 8 microsatellites (Faulks et al. 2011). Understanding the origin of the two clusters is important for the Victorian Macquarie perch breeding program run by Victorian fisheries since 2009, because the Dartmouth and Yarra populations are the largest in Victoria and currently the only sources of broodstock (Ho and Ingram 2012).

References:

Faulks, L. K., D. M. Gilligan, and L. B. Beheregaray. 2010. Evolution and maintenance of divergent lineages in an endangered freshwater fish, *Macquaria australasica*. Conservation Genetics 11:921-934.

Faulks, L. K., D. M. Gilligan, and L. B. Beheregaray. 2011. The role of anthropogenic vs. natural in-stream structures in determining connectivity and genetic diversity in an endangered freshwater fish, Macquarie perch (*Macquaria australasica*). Evol Appl 4:589–601.

Frankham, R., J. D. Ballou, M. D. B. Eldridge, R. C. Lacy, K. Ralls, M. R. Dudash, and C. B. Fenster. 2011. Predicting the probability of outbreeding depression. Conserv Biol 25:465-475.

Ho, H. K. and B. A. Ingram. 2012. Genetic risk assessment for stocking Macquarie perch into Victorian waterways 2011. Pp. 24. Fisheries Victoria Internal Report No. 45. Fisheries Victoria.

Nguyen, T. T. T., B. A. Ingram, J. Lyon, K. Guthridge, and J. Kearns. 2012. Genetic diversity of populations of Macquarie perch, *Macquaria australasica*, in Victoria. Pp. 14. Fisheries Victoria Internal Report No. 40. Fisheries Victoria.

Pavlova, A., H. M. Gan, Y. P. Lee, C. M. Austin, D. Gilligan, M. Lintermans, and P. Sunnucks. accepted. Purifying selection and genetic drift shaped Pleistocene evolution of the mitochondrial genome in an endangered Australian freshwater fish. Heredity HDY-16-OR0171R, accepted 3/11/16.

Rand, D. M., R. A. Haney, and A. J. Fry. 2004. Cytonuclear coevolution: the genomics of cooperation. Trends Ecol Evol 19:645-653.

Tonkin, Z., J. Kearns, J. Mahoney, and J. Mahony. 2015. Spatio-temporal spawning patterns of two riverine populations of the threatened Macquarie perch *Macquaria australasica*. Mar Freshw Res Published online: 23 November 2015; <http://dx.doi.org/10.1071/MF15319>.

Verhoeven, K. J., M. Macel, L. M. Wolfe, and A. Biere. 2011. Population admixture, biological invasions and the balance between local adaptation and inbreeding depression. Proceedings of the Royal Society of London B: Biological Sciences 278:2-8.

Appendix 2. Estimates of nuclear and mitochondrial genetic diversity (Table S2A) and effective population sizes (Table S2B, Fig. S2A).

Table S2A. Estimates of nuclear genetic diversity (mean values across 19 microsatellites) for 20 populations (N=871) and of mitochondrial genetic diversity (844bp of control region) for 17 populations (N=339). S/s- sample sizes, Poly loci- number of polymorphic loci, N alleles- mean number of different alleles across loci, N eff all- mean number of effective alleles across loci (the inverse of the homozygosity), N private all- mean number of private alleles across loci; Ho-observed heterozygosity, He- expected heterozygosity (average gene diversity over loci), AR- allelic richness standardized to N=14, S- number of polymorphic sites, N haps- number of control region haplotypes, Hd- haplotype diversity, π- nucleotide diversity, Taj D- Tajima’s D, asterisk indicate significant values (P<0.05 for Tajima’s D, P<0.02 for Fu’s Fs), ns- not significant, na- not applicable. Estimates of mtDNA diversity for three populations not sequences here, obtained from partial control region sequence of Faulks et al. (2010), were: Wollemi: s/s=18, S=2, N haps=2, Hd=0.111, π=0.0006; Erskine: s/s=7, S=0, N haps=1, Hd=0, π=0; Kowmung: s/s=23, S=9, N haps=6, Hd=0.708, π=0.0076; Taj D and Fu’s Fs n/s for all three.

| Drainage Basin | Population | s/s _msat_ | Poly loci | N alleles | N eff all | N private all | Ho | He | AR | s/s _mtDNA_ | S | N haps | Hd | π | Taj D,  Fu’s Fs |
| --- | --- | --- | --- | --- | --- | --- | --- | --- | --- | --- | --- | --- | --- | --- | --- |
| Northern | Wollemi Creek | 16 | 12 | 2.32 | 1.48 | 0.21 | 0.217 | 0.213 | 2.276 |  |  |  |  |  |  |
| HNB | Wheeny Creek | 20 | 6 | 1.37 | 1.18 | 0.05 | 0.088 | 0.083 | 1.368 | 7 | 0 | 1 | 0 | 0 | ns, na |
| Southern HNB | Glenbrook Creek | 30 | 10 | 2.16 | 1.48 | 0.11 | 0.215 | 0.196 | 1.981 | 11 | 0 | 1 | 0 | 0 | *, ns |
|  | Erskine Creek | 19 | 15 | 3.42 | 2.22 | 0.26 | 0.401 | 0.399 | 3.251 |  |  |  |  |  |  |
|  | Kowmung River | 24 | 14 | 4.32 | 2.34 | 0.47 | 0.392 | 0.400 | 3.758 |  |  |  |  |  |  |
|  | Little River | 25 | 16 | 4.05 | 2.13 | 0.26 | 0.360 | 0.371 | 3.529 | 11 | 8 | 5 | 0.855 | 0.0041 | ns, ns |
| admixed | Cataract River | 65 | 19 | 5.21 | 2.94 | 0.37 | 0.585 | 0.596 | 4.081 | 54 | 17 | 5 | 0.591 | 0.0079 | ns, ns |
| HNB, MDB introduction | Cataract Dam | 58 | 19 | 3.32 | 2.19 | 0 | 0.476 | 0.472 | 2.933 | 15 | 6 | 2 | 0.514 | 0.0037 | ns, ns |
| MDB | Abercrombie River | 31 | 19 | 3.84 | 2.62 | 0.05 | 0.470 | 0.505 | 3.525 | 6 | 0 | 1 | 0 | 0 | ns, na |
|  | Lachlan River | 25 | 19 | 3.95 | 2.60 | 0.05 | 0.520 | 0.513 | 3.659 | 5 | 2 | 2 | 0.400 | 0.0009 | ns, ns |
|  | Adjungbilly Creek | 23 | 12 | 1.68 | 1.43 | 0 | 0.261 | 0.234 | 1.662 | 23 | 5 | 4 | 0.320 | 0.0006 | *, ns |
|  | Cotter River | 129 | 14 | 2.00 | 1.49 | 0 | 0.277 | 0.261 | 1.834 | 15 | 0 | 1 | 0 | 0 | ns, na |
|  | Murrumbidgee River | 30 | 11 | 1.79 | 1.36 | 0.05 | 0.186 | 0.194 | 1.752 | 11 | 0 | 1 | 0 | 0 | ns, na |
|  | Dartmouth Reservoir | 122 | 19 | 5.58 | 2.72 | 0.16 | 0.502 | 0.493 | 4.040 | 52 | 6 | 6 | 0.706 | 0.0017 | ns, ns |
|  | Hollands Creek | 30 | 19 | 4.00 | 2.40 | 0 | 0.512 | 0.493 | 3.517 | 15 | 10 | 7 | 0.876 | 0.0042 | ns, ns |
|  | Sevens Creek | 30 | 19 | 4.32 | 2.39 | 0.16 | 0.502 | 0.514 | 3.782 | 15 | 6 | 4 | 0.552 | 0.0019 | ns, ns |
|  | Hughes Creek | 27 | 19 | 3.89 | 2.18 | 0.16 | 0.439 | 0.453 | 3.484 | 15 | 12 | 5 | 0.810 | 0.0053 | ns, ns |
|  | King Parrot Creek | 30 | 15 | 3.53 | 2.43 | 0 | 0.408 | 0.432 | 3.199 | 15 | 12 | 4 | 0.657 | 0.0063 | ns, ns |
| Yarra Basin, MDB introduction | Yarra River | 136 | 19 | 5.68 | 2.87 | 0.21 | 0.512 | 0.520 | 4.213 | 68 | 23 | 16 | 0.869 | 0.0059 | ns, ns |
| Shoalhaven Basin, presumed extinct | Kangaroo River | 1 |  |  |  |  |  |  |  | 1 |  |  |  |  |  |

Table S2B. LDNe and OneSamp estimates of contemporary effective population sizes for HNB and MDB populations based on microsatellites, and approximate population sizes. LDNe estimates from sample sizes of 50 or more (black font) are assumed to reliably estimate effective population sizes (Tallmon et al. 2008), small Ne (<100, blue font) could be estimated with smaller sample sizes (Waples and Do, 2008) (see Fig. S2A). OneSamp results are shown for two sets of Ne priors (4-1000 and 4-500) used for OneSamp estimates; low-high CI- parametric confidence interval of linkage disequilibrium-based Ne estimates (values for s/s<50 are in grey and not interpreted); N loci- number of variable loci used for OneSamp analysis. Mean values <100 are in red, <50 are in bold. Approximate population sizes are calculated from OneSamp estimates with 4-1000 prior, as described in Discussion of the main manuscript: mean=44.4*mean *N*_e_; lower bound=6.27* low 95%CL *N*_e_; upper bound=82.6*upper 95%CL *N*_e_ (Fig. S2A).

|  |  |  | LDNe Ne | |  | OneSamp Ne, 4-1000 prior on Ne | | | OneSamp Ne, 4-500 prior on Ne | | | Approximate population sizes | | |
| --- | --- | --- | --- | --- | --- | --- | --- | --- | --- | --- | --- | --- | --- | --- |
|  | Population | N inds | mean | low-high CI | N loci | mean | lower 95% CL | upper 95% CL | mean | lower 95% CL | upper 95% CL | mean | lower bound | upper bound |
| Northern | Wollemi Creek | 16 | -1051 | 18- inf | 11 | **20** | 16 | 33 | **24** | 19 | 37 | 888 | 100 | 2726 |
| HNB | Wheeny Creek | 20 | 1 | 0.4-3 | 5 | **7** | 5 | 13 | **8** | 6 | 13 | 311 | 31 | 1074 |
| Southern HNB | Glenbrook Creek | 29 | 3776 | 28- inf | 10 | **27** | 22 | 49 | **28** | 23 | 38 | 1199 | 138 | 4047 |
|  | Erskine Creek | 19 | 675 | 44- inf | 14 | **21** | 16 | 32 | **21** | 18 | 31 | 932 | 100 | 2643 |
|  | Kowmung River | 24 | -395 | 123- inf | 13 | 51 | 38 | 98 | **43** | 34 | 66 | 2264 | 238 | 8095 |
|  | Little River | 25 | 62 | 34-228 | 15 | 90 | 62 | 234 | **42** | 34 | 75 | 3996 | 389 | 19328 |
| Admixed | Cataract River | 65 | 164 | 103-349 | 18 | **38** | 29 | 65 | **35** | 28 | 49 | 1687 | 182 | 5369 |
| MDB introduction to HNB | Cataract Dam | 56 | 128 | 68-501 | 18 | 71 | 55 | 165 | 50 | 41 | 77 | 3152 | 345 | 13629 |
| MDB | Abercrombie River | 31 | 104 | 50-1187 | 18 | **48** | 39 | 84 | **48** | 40 | 73 | 2131 | 245 | 6938 |
|  | Lachlan River | 25 | 41 | 25-84 | 18 | **28** | 24 | 42 | **26** | 23 | 32 | 1243 | 150 | 3469 |
|  | Adjungbilly Creek | 23 | 25 | 6- inf | 11 | **24** | 20 | 41 | **22** | 18 | 30 | 1066 | 125 | 3387 |
|  | Cotter River | 129 | 63 | 35-134 | 14 | **41** | 33 | 72 | **28** | 23 | 38 | 1820 | 207 | 5947 |
|  | Murrumbidgee River | 30 | 7 | 3-16 | 11 | **23** | 18 | 42 | **23** | 18 | 33 | 1021 | 113 | 3469 |
|  | Dartmouth Reservoir | 122 | 307 | 201-593 | 18 | 86 | 69 | 144 | 56 | 48 | 77 | 3818 | 433 | 11894 |
|  | Hollands Creek | 29 | 22 | 16-32 | 17 | **30** | 26 | 41 | **23** | 20 | 30 | 1332 | 163 | 3387 |
|  | Sevens Creek | 30 | 33 | 23-52 | 18 | **39** | 32 | 60 | **33** | 28 | 43 | 1732 | 201 | 4956 |
|  | Hughes Creek | 27 | 214 | 60- inf | 18 | **43** | 34 | 73 | **22** | 18 | 30 | 1909 | 213 | 6030 |
|  | King Parrot Creek | 30 | 20 | 14-29 | 15 | **37** | 29 | 75 | **34** | 28 | 55 | 1643 | 182 | 6195 |
| MDB introduction to Yarra Basin | Yarra River | 136 | 344 | 228-642 | 18 | 132 | 104 | 234 | 84 | 71 | 122 | 5861 | 652 | 19328 |

References:

Tallmon, D. A., A. Koyuk, G. Luikart, and M. A. Beaumont. 2008. ONeSAMP: a program to estimate effective population size using approximate Bayesian computation. Molecular Ecology Resources 8:299-301

Waples, R. S. and C. Do. 2008. LDNE: a program for estimating effective population size from data on linkage disequilibrium. Molecular Ecology Resources 8:753-756.

Figure S2A. Estimates of effective population sizes (left panels, *N*_e_ from Table S2B) and approximate population sizes (right panel) from LDNe (top panels) and OneSamp (bottom panels); coloured bars- ranges, black bars- means, colours correspond to 19 populations, as on Figs 1 and 2 of the main manuscript. LDNe *N*_e_ and N estimates are shown for five populations with sample size >50 individuals and six additional populations with small *N*_e_ estimates (upper 95% CI <100). Adjusted Ne scales are 1.6*mean LDNe *N*_e_ for LDNe and 4.5*mean OneSamp *N*_e_ for OneSamp (see Discussion; lower and upper bounds are not adjusted). Approximate population sizes are calculated as described in Discussion of the main manuscript: from LDNe: mean= 9.4*mean *N*_e_; lower bound= 4.18*low CI *N*_e_; upper bound= 19.2*high CI *N*_e_; from OneSamp: mean=44.4*mean *N*_e_; lower bound=6.27* low 95%CL *N*_e_; upper bound=82.6*upper 95%CL *N*_e_ (Table S2B).


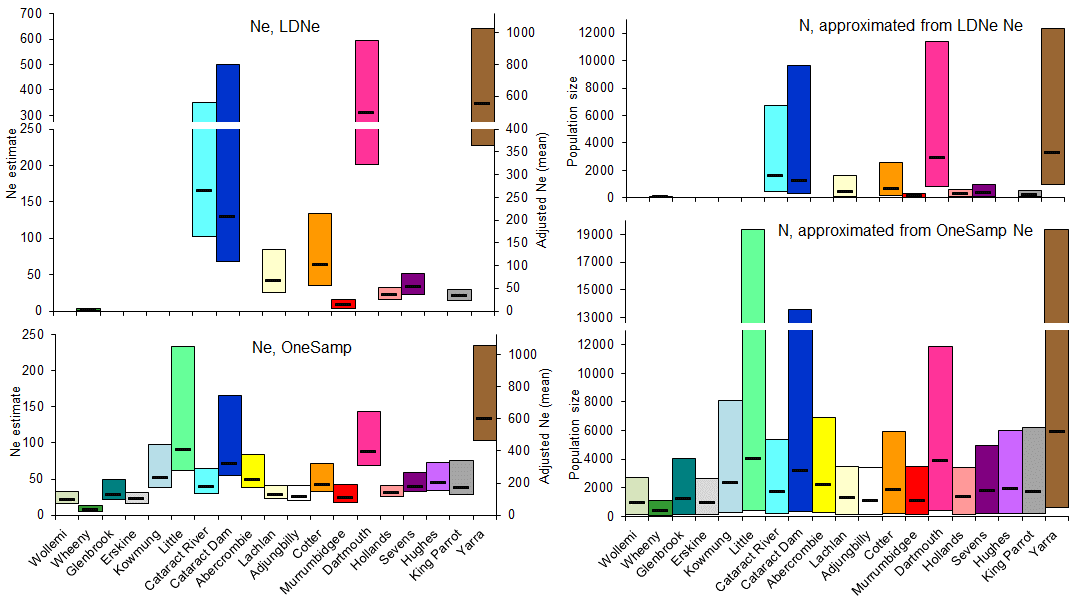


Appendix 3. Details of laboratory analyses and results of Hardy-Weinberg and linkage disequilibrium tests.

Majority of samples were not previously analyzed for mitochondrial or microsatellite data. Of 339 sequenced individuals, three from Abercrombie and one from the Shoalhaven Basin (Kangaroo River) were used in Faulks, Gilligan, and Beheregaray (2010), 17 were used in a mitogenome study (Pavlova et al., accepted). Of 871 genotyped individuals 177 were genotyped for 8 loci by Faulks et al. (2011) and 142 were genotyped for 8 loci by Nguyen et al. (2012).

PCRs for mitochondrial control region were performed in 25 µL volumes and included 1x HotStar Taq Master Mix (QIAGEN), 0.4 µM primers and ~20 ng of genomic DNA. PCR conditions included initial denaturation at 95°C for 15 min, 35 cycles of 94°C for 30 sec, 50°C for 30 sec, and 72°C for 1 min, and final extension at 72°C for 10 min. PCR products were cleaned using ExoSAP-IT® (Affymetrix) and sequenced commercially (Macrogen, Korea) in both directions using amplification primers.

Twenty two microsatellite loci were initially trialled: AB009 developed for Australian bass (Schwartz et al., 2005), LFMP003, LFMP011, LFMP042, LFMP052, LFMP054, LFMP072, LFMP100, LFMP106, LFMP107 developed for Macquarie perch (Farrington, 2011), and Mpe1.A07, Mpe1.B10, Mpe1.F01, Mpe1.H04, Mpe2.B02, Mpe2.D11, Mpe2.E01, Mpe2.F01, Mpe2.F07, Mpe3.B11, Mpe3.G04, and Mpe3.G12 developed for Murray cod (Rourke et al., 2007). Multiplex Manager 1.2 (Holleley & Geerts, 2009) was used to optimize PCRs into three multiplex reactions, which were run in total reaction volume of 5 µL, and contained 1x Mango Taq Reaction Buffer (Bioline), 0.2 mM dNTPs, primers and MgCl2 (Bioline) in variable concentrations (Table S3A), 0.25U of Q solution (QIAGEN), 0.25U of Mango Taq DNA Polymerase (Bioline) and 10 ng of genomic DNA. PCR conditions included initial denaturation at 94°C for 15 min, 30 cycles of 94°C for 30s, annealing temperature (53°C for multiplex A, 57°C for B and C) for 90s, and 72°C for 60s, and final extension at 72°C for 5min. PCR products were genotyped on AB 3130xl Genetic Analyser and fragments analysed using GeneMapper v 4.1 (Applied Biosystems). After preliminary analyses, three loci were excluded due to suspected null alleles (Mpe2.F01, Mpe2.E01 and LFMP107). Absence of null alleles and large allele dropout in other loci were confirmed using Micro-Checker v 2.2.3 (Van Oosterhout et al. 2004).

For 19 populations genotyped for 19 microsatellite loci, only five of 288 Hardy–Weinberg (HW) tests were significant after sequential Bonferroni correction (P<0.0002). Of these, four tests had *F*_IS_>0 indicating significant homozygote excess: locus Mpe3.G12 in Kowmung and Mpe3.B11 in Cataract River, King Parrot and Little. At P<0.05, 28 HW tests were significant, including two more for locus Mpe3.B11 (Hughes and Dartmouth; *F*_IS_<0 for both) and two for Mpe3.G12 (Wheeny and Hughes; *F*_IS_>0). Because evidence of HW disequilibrium was weak and localized (and, at least in the admixed Cataract River population likely reflected population substructure [Walhund effect]), we retained all 19 loci in the analyses.

There was no strong evidence of linkage disequilibrium. Only four of 2297 pairwise locus combinations were significant after Bonferroni correction (P< 0.00002), all of these involved Lachlan population, potentially suggesting presence of substructure within Lachlan sample. At P<0.05, no more than four populations showed linkage disequilibrium (LD) for each pairwise locus combination, and combinations of populations did not indicate a consistent pattern.

Table S3A. Loci and primer concentrations for three multiplex PCR reactions used for microsatellite genotyping of Macquarie perch.

| **Multiplex** | **Locus** | **Fluorescent dye** | **Primer concentration (µM)** | **MgCl2 concentration (mM)** |
| --- | --- | --- | --- | --- |
| A | AB009 | FAM | 0.1 | 3 |
|  | LFMP100 | FAM | 0.1 |  |
|  | Mpe2.F01 | FAM | 0.2 |  |
|  | Mpe3.G12 | NED | 0.1 |  |
|  | LFMP072 | VIC | 0.1 |  |
|  | LFMP052 | PET | 0.1 |  |
| B | Mpe1.F01 | FAM | 0.1 | 1.5 |
|  | LFMP003 | FAM | 0.1 |  |
|  | LFMP107 | FAM | 0.1 |  |
|  | Mpe1.A07 | NED | 0.1 |  |
|  | Mpe1.H04 | VIC | 0.1 |  |
|  | Mpe2.D11 | VIC | 0.15 |  |
|  | Mpe1.B10 | PET | 0.1 |  |
|  | Mpe2.E01 | PET | 0.2 |  |
| C | Mpe2.F07 | FAM | 0.2 | 1.5 |
|  | Mpe3.B11 | NED | 0.1 |  |
|  | LFMP054 | NED | 0.1 |  |
|  | Mpe2.B02 | VIC | 0.1 |  |
|  | LFMP011 | VIC | 0.1 |  |
|  | Mpe3.G04 | PET | 0.1 |  |
|  | LFMP042 | PET | 0.1 |  |
|  | LFMP106 | PET | 0.1 |  |

References:

Farrington, L.W. 2011. Microsatellite markers for the threatened Australian freshwater fish, Macquarie Perch (*Macquaria australasica*). Conservation Genetics Resources 4 (2):235-237.

Faulks, L.K., D.M. Gilligan, and L.B. Beheregaray. 2011. The role of anthropogenic vs. natural in-stream structures in determining connectivity and genetic diversity in an endangered freshwater fish, Macquarie perch (Macquaria australasica). Evolutionary Applications 4 (4):589–601.

Faulks, L.K., D.M. Gilligan, and L.B. Beheregaray. 2010. Evolution and maintenance of divergent lineages in an endangered freshwater fish, *Macquaria australasica*. Conservation Genetics 11 (3):921-934.Holleley, Clare E and Paul G Geerts. 2009. Multiplex Manager 1.0: a cross-platform computer program that plans and optimizes multiplex PCR. BioTechniques 46 (7):511-517.

Nguyen, T.T.T., B.A. Ingram, J. Lyon, K. Guthridge, and J. Kearns. 2012. Genetic diversity of populations of Macquarie perch, *Macquaria australasica*, in Victoria. In Fisheries Victoria Internal Report No. 40: Fisheries Victoria.

Pavlova, A., H. M. Gan, Y. P. Lee, C. M. Austin, D. Gilligan, M. Lintermans, and P. Sunnucks. accepted. Purifying selection and genetic drift shaped Pleistocene evolution of the mitochondrial genome in an endangered Australian freshwater fish. Heredity HDY-16-OR0171R, accepted 3/11/16.

Rourke, M., J. Nheu, H. Mountford, J. Lade, B. Ingram, and H. Mcpartlan. 2007. Isolation and characterization of 102 new microsatellite loci in Murray cod, *Maccullochella peelii peelii* (Percichthyidae), and assessment of cross-amplification in 13 Australian native and six introduced freshwater species. Molecular Ecology Notes 7 (6):1258-1264.

Schwartz, T.S., F. Jenkins, and L.B. Beheregaray. 2005. Microsatellite DNA markers developed for the Australian bass (*Macquaria novemaculeata*) and their cross-amplification in estuary perch (Macquaria colonorum). Molecular Ecology Notes 5 (3):519-520.

Van Oosterhout, C., W.F. Hutchinson, D.P.M. Wills, and P. Shipley. 2004. MICRO-CHECKER: software for identifying and correcting genotyping errors in microsatellite data. Molecular Ecology Notes 4 (3):535-538.

Appendix 4. Environmental variables used for modelling genetic diversity, pairwise correlations between them (Table S4A) and variation at seven variables used for final analyses and heterozygosity-by-locus (genetic response) across 19 Macquarie perch populations (Fig. S4A).

The following eleven variables used in the model; the first ten were derived from variables of National Environmental Stream Attributes Database v1.1.5, and the last (tmean_11) was sourced from Bureau of Meteorology http://www.bom.gov.au):

RUNANNMEAN (ml): Annual mean accumulated soil water surplus,

RUNMTHCOFV: Coefficient of variation of monthly totals of accumulated soil water surplus;

FRDI (score between zero and one): Flow regime disturbance index calculated for period 1970-2008,

CONLEN (%)*TOTLEN(km)/100%: Barrier free flow path length percentage* Total catchment length/100%; a barrier free flow path length in kilometres (dubbed CONLENKM (km)), where barriers considered were reservoirs, damwalls, spillways or large dams,

DISTUPDAMW (km): Maximum barrier free flow path length upstream (damwalls, spillways or large dams),

STRELEMEAN (m): Mean segment elevation,

VALLEYSLOPE (%): Stream segment slope,

STRWOODLANDS-EXT(%)+ STRFORESTS-EXT(%): Stream and valley percentage extant woodland cover + Stream and valley percentage extant forests cover; stream and valley percentage extant woodland and forest cover (areal proportion of grid cells comprising the stream segment and associated valley bottom with extant woodland and forests cover, dubbed STRFORWOOD_EXT (%)),

STRCOLDMTHMIN (°C): Stream and environs average coldest month minimum temperature (i.e. minimum temperature of all grid cells comprising the stream segment and associated valley bottoms),

STRHOTMTHMAX (°C): Stream and environs average hottest month maximum temperature,

tmean_11 (°C): Mean November temperature

The choice of variables was justified as follows. Variables describing river flow and its variability (mean total annual precipitation and mean coefficient of variation of mean annual precipitation) predicted presence of Macquarie perch in Victoria (Chee and Elith, 2012). Flow regime disturbance index was shown to be a useful predictor of presence (Bond et al., 2011) and genetic diversity (Harrisson et al., 2016) for some fish. River regulation is often reported as having detrimental effects on range and abundance of native riverine fishes (Koehn et al., 2014; Mims and Olden, 2013; Poff et al., 2007), but disturbance to the natural flow regime can be positively associated with genetic diversity (Faulks et al., 2010; Harrisson et al., 2016). Anthropogenic barriers (e.g. dams and weirs) reduce populations to small remnants by restricting fish movement and positively correlate with population genetic differentiation in Macquarie perch (Faulks et al., 2011). For co-distributed Murray cod *Maccullochella peelii*, spring/summer releases of cold water from large irrigation supply dams result in high mortality of eggs and larvae up to 30-50 km downstream (Todd et al., 2005) and could similarly impact Macquarie perch. Abundance of Macquarie perch within the MDB is correlated with elevation: the species was formerly present in the lower reaches of the Murray River ~ 30 m above sea level, common in reaches from ~100 m, abundant above ~ 200 m, and occurred up to a maximum altitude of 1100 m in the Murrumbidgee River (Gilligan et al., 2010). Shallow riffles or gravel beds are required for spawning and deep pools with in-stream snags or boulders are the preferred habitat of Macquarie perch (Lintermans, 2007; Lintermans and Ebner, 2010). River slope was a significant predictor of presence (Chee and Elith, 2012) and genetic diversity of Macquarie perch (Faulks et al., 2011). Mean temperature of warmest quarter was associated with presence of Macquarie perch in Victoria (Chee and Elith, 2012). Average spring temperature was shown to be one of the most important environmental factors of health of native freshwater fish populations in Victoria (Koehn and O'Connor, 1990). November is the core spawning period for Macquarie perch, whose spawning is temperature cued (Appleford et al., 1998; Cadwallader and Rogan, 1977; Koster et al., 2013; Tonkin et al., 2010).

References:

Appleford, P., Anderson, T., Gooley, G., 1998. Reproductive cycle and gonadal development of Macquarie perch, *Macquaria australasica* Cuvier (Percichthyidae), in Lake Dartmouth and tributaries of the Murray–Darling Basin, Victoria, Australia. Mar Freshw Res 49, 163-169.

Bond, N., Thomson, J., Reich, P., Stein, J., 2011. Using species distribution models to infer potential climate change-induced range shifts of freshwater fish in south-eastern Australia. Mar Freshw Res 62, 1043–1061.

Cadwallader, P., Rogan, P., 1977. The Macquarie perch, *Macquria australasica* (Pisces: Percichthyidae), of Lake Eildon, Victoria. Aust J Ecol 2, 409-418.

Chee, Y.E., Elith, J., 2012. Spatial data for modelling and management of freshwater ecosystems. International Journal of Geographical Information Science 26, 2123-2140.

Faulks, L.K., Gilligan, D.M., Beheregaray, L.B., 2010. Islands of water in a sea of dry land: hydrological regime predicts genetic diversity and dispersal in a widespread fish from Australia's arid zone, the golden perch (*Macquaria ambigua*). Mol Ecol 19, 4723-4737.

Faulks, L.K., Gilligan, D.M., Beheregaray, L.B., 2011. The role of anthropogenic vs. natural in-stream structures in determining connectivity and genetic diversity in an endangered freshwater fish, Macquarie perch (*Macquaria australasica*). Evol Appl 4, 589–601.

Gilligan, D., McGarry, T., Carter, S., 2010. A scientific approach to developing habitat rehabilitation strategies in aquatic environments: A case study on the endangered Macquarie perch (*Macquaria australasica*) in the Lachlan catchment. A report to the Lachlan Catchment Management Authority. Department of Industry and Investment (Industry & Investment NSW).

Harrisson, K.A., Yen, J.D.L., Pavlova, A., Rourke, M.L., Gilligan, D.M., Ingram, B., Lyon, J., Tonkin, Z., Sunnucks, P., 2016. Identifying environmental correlates of intra-specific genetic variation. Heredity 117: 155-164.

Koehn, J., O'Connor, W., 1990. Biological information for management of native freshwater fish in Victoria. Department of Conservation and Environment, Freshwater Fish Management Branch, Arthur Rylah Institute for Environmental Research.

Koehn, J.D., King, A.J., Beesley, L., Copeland, C., Zampatti, B.P., Mallen-Cooper, M., 2014. Flows for native fish in the Murray-Darling Basin: lessons and considerations for future management. Ecological Management & Restoration 15, 40-50.

Koster, W., Dawson, D., Morrongiello, J., Crook, D., 2013. Spawning season movements of Macquarie perch (*Macquaria australasica*) in the Yarra River, Victoria. Aust J Zool 61, 386-394.

Lintermans, M., 2007. Fishes of the Murray-Darling Basin: an introductory guide. Murray-Darling Basin Commission Canberra.

Lintermans, M., Ebner, B., 2010. Threatened Fish Profile:‘Western’ Macquarie perch *Macquaria australasica* Cuvier 1830. Australian Society for Fish Biology Newsletter 40(2): 76-78.

Mims, M.C., Olden, J.D., 2013. Fish assemblages respond to altered flow regimes via ecological filtering of life history strategies. Freshw Biol 58, 50-62.

Poff, N.L., Olden, J.D., Merritt, D.M., Pepin, D.M., 2007. Homogenization of regional river dynamics by dams and global biodiversity implications. Proceedings of the National Academy of Sciences 104, 5732-5737.

Todd, C.R., Ryan, T., Nicol, S.J., Bearlin, A.R., 2005. The impact of cold water releases on the critical period of post-spawning survival and its implications for Murray cod (*Maccullochella peelii peelii*): a case study of the Mitta Mitta River, southeastern Australia. River Research and Applications 21, 1035-1052.

Tonkin, Z., Lyon, J., Pickworth, A., 2010. Spawning behaviour of the endangered Macquarie perch *Macquaria australasica* in an upland Australian river. Ecological Management & Restoration 11, 223-226

Table S4A: Pearson correlations among variables, removed variables are shaded.

|  | tmean_11_AU | DISTUPDAMW | CONLENKM | STRCOLDMTHMIN | STRHOTMTHMAX | FRDI | RUNANNMEAN | RUNMTHCOFV | STRELEMEAN | VALLEYSLOPE |
| --- | --- | --- | --- | --- | --- | --- | --- | --- | --- | --- |
| DISTUPDAMW | -0.36 |  |  |  |  |  |  |  |  |  |
| CONLENKM | -0.26 | 0.61 |  |  |  |  |  |  |  |  |
| STRCOLDMTHMIN | 0.12 | 0.37 | 0.07 |  |  |  |  |  |  |  |
| STRHOTMTHMAX | 0.30 | -0.31 | 0.07 | -0.67 |  |  |  |  |  |  |
| FRDI | -0.45 | 0.38 | 0.01 | 0.60 | **-0.88** |  |  |  |  |  |
| RUNANNMEAN | -0.44 | **0.78** | 0.37 | 0.61 | -0.68 | **0.77** |  |  |  |  |
| RUNMTHCOFV | 0.68 | -0.51 | -0.36 | -0.43 | 0.50 | **-0.70** | **-0.72** |  |  |  |
| STRELEMEAN | -0.41 | -0.29 | -0.02 | **-0.91** | 0.44 | -0.34 | -0.46 | 0.17 |  |  |
| VALLEYSLOPE | 0.18 | -0.58 | -0.36 | -0.39 | 0.20 | -0.27 | -0.51 | 0.29 | 0.37 |  |
| STRFORWOOD_EXT | 0.66 | -0.37 | -0.45 | 0.11 | -0.04 | -0.14 | -0.27 | 0.53 | -0.30 | 0.11 |

Figure S4A. Variation at seven environmental predictor variables and heterozygosity-by-locus (genetic response) across 19 Macquarie perch populations. Blue font indicates populations located on a coastal side of the Great Dividing Range (HNB and Yarra), red- inland (MDB).


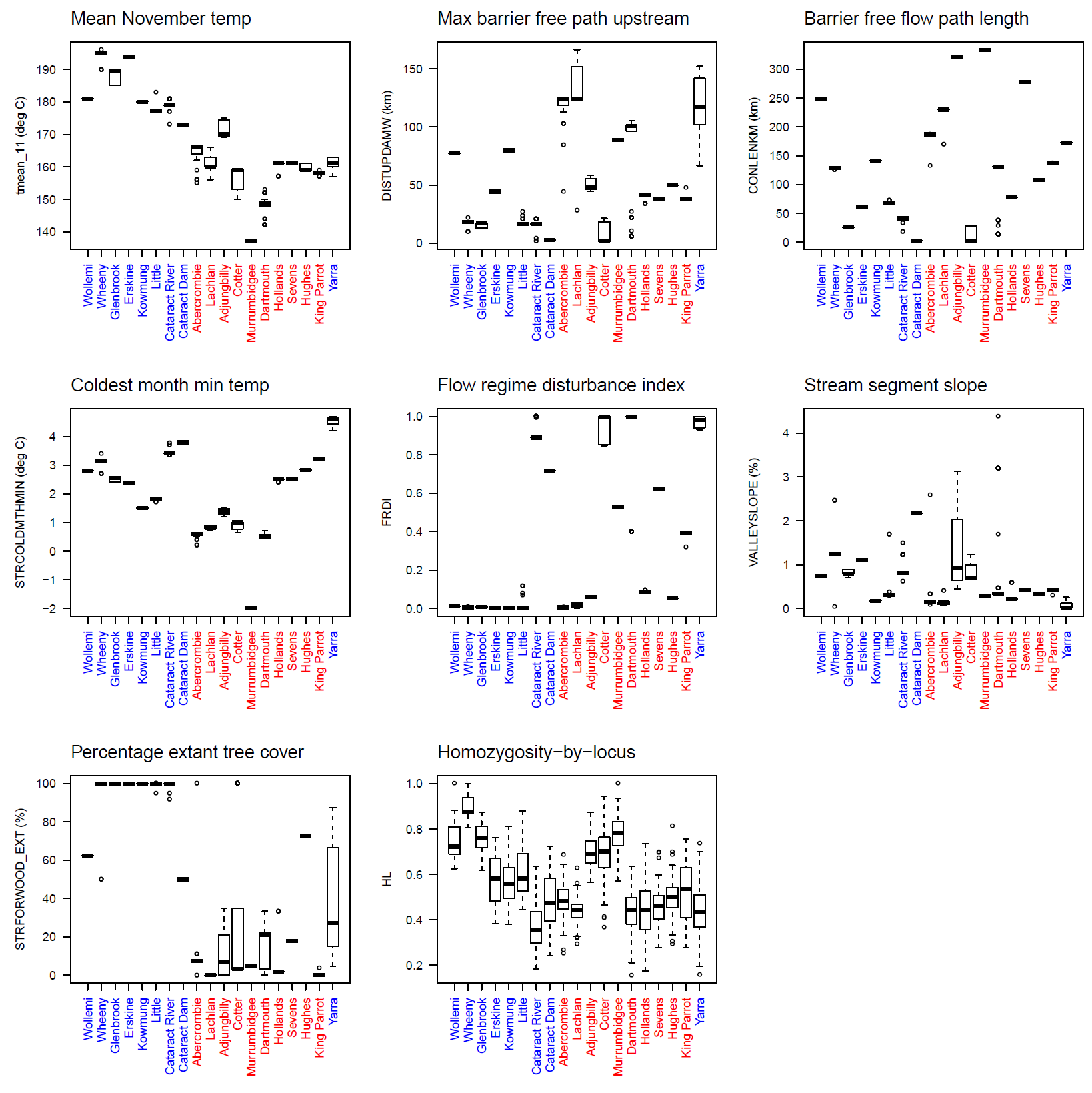


Appendix 5. Environmental model details, WinBUGS code and results (Fig. S5A).

*Estimating relationships between individual genetic diversity and environmental variables*

We used a hierarchical Bayesian regression model to estimate the relationships between HL and environmental variables. We used reversible-jump Markov chain Monte Carlo (MCMC) to perform model selection (i.e., determining which variables are important). Reversible-jump MCMC moves between different possible models and, in so doing, estimates the posterior probability that a given variable was included in the fitted model (Lunn et al., 2009; Lunn et al., 2006). Our model allowed linear, quadratic and cubic relationships between HL and the included environmental variables. Reversible-jump MCMC covers a large suite of possible models and Bayesian model averaging was used to combine these possible models into a final fitted model, with the contribution of each possible model proportional to its posterior probability (Raftery et al., 1997). Basin and site were included as random effects in the model to account for spatial clustering. We assumed that HL was normally distributed and standardised all predictor variables to zero mean and unit variance.

The general model was

*y_i,j_ = β_0_ + β_1_(X_s(j),1_) + … + β_k_(X_s(j),k_) + γ_1_*_,_*_b(j)_ + γ_2_*_,_*_j_ + ε_i,j_;*

where *y_i,j_* is the response variable (HL) for sample *i* in site *j*, *X_s(j),k_* is the value of predictor variable *k* in stream segment *s(j)*, *β_k_(X_s(j),k_)* is a function representing the relationship between predictor variable *k* and HL, *γ_1_*_,_*_b(j)_* and *γ_2_*_,_*_j_* are random effects terms that account for differences among basins [*b(j)*] and sites (*j*), respectively, and *ε_i,j_* is the residual, which was assumed to be normally distributed with zero mean and unknown variance σ*^2^*.

The relationship between predictor variable *k* and HL, *β_k_(X_s(j),k_)*, was estimated using a linear spline, which approximates a higher-order polynomial function using a set of linear segments. The form of this spline was

*β_k_(X_s(j),k_)* = Σ*_p_* δ*_k_*_,_*_p_* (*X_s(j),k_* – θ*_k_*_,_*_p_*)_+_,

where θ*_k_*_,_*_p_* is the location of the *p^th^* knot for predictor variable *k*, δ*_k_*_,_*_p_* is the slope of the *p^th^* segment for predictor variable k, and (*x*)_+_ equals *x* if *x* is greater than zero and equals zero otherwise. The variable *p* was allowed to equal 0, 1, 2, or 3, which approximates no effect, a linear effect, a quadratic effect, or a cubic effect, respectively. The prior for *p* was a discrete distribution, with Pr(*p =* 0) = 0.5, Pr(*p =* 1) = 0.3, Pr(*p =* 2) = 0.1 and Pr(*p =* 3) = 0.1. This prior distribution meant that all combinations of variables were equally likely a priori. The positions of the knots, θ*_k_*_,_*_p_*, were assigned discrete uniform priors with ten possible positions spaced evenly over the range of the relevant predictor variable.

The parameters of the models were estimated using reversible-jump Markov chain Monte Carlo (MCMC), implemented in the jump add-in for WinBUGS 1.4 (Lunn et al., 2009; Lunn et al., 2000; Lunn et al., 2006). Reversible-jump MCMC is a method for sampling from distributions that do not have fixed dimension (Lunn et al., 2009; Lunn et al., 2006). In our model, the value of *p* determines the dimension of the coefficient δ, which can range from zero to three times the number of available predictor variables. Reversible-jump MCMC handles the ‘transdimensional’ nature of this model in a straightforward manner and, additionally, estimates the posterior probability of each possible model (each possible combination of variables), which can be used to calculate Bayesian model averaged parameter estimates.

The overall variance, σ*^2^*, was assigned an inverse-Gamma prior distribution with both parameters equal to 0.001. The regression parameters, δ*_k_*_,_*_p_*, were assigned a half-Cauchy prior, scaled such that the majority (95%) of the prior probability mass for each δ*_k_*_,_*_p_* value was in the interval [–2, 2]. This interval was thought to represent a plausible range of parameter values for the regression coefficients. Changes to the scaling of the half-Cauchy prior distribution did not affect model outputs. Each exchangeable (random effect) term was assigned a Gaussian prior, with zero mean and common variance. This variance was assigned an inverse-Gamma prior, with both parameters equal to 0.001. A sum-to-zero constraint was used for each exchangeable term (i.e., Σ*_j_ γ_2_*_,_*_j_* = 0).

Inferences were based on the Bayesian model averaged parameter estimates. Bayesian model averaging takes the average parameter estimate from each possible model (i.e., each possible combination of variables), where the contribution of each possible model is weighted by its posterior probability (Raftery et al., 1997). Model posterior probabilities emerge naturally from a reversible-jump MCMC sampling scheme because the sampler visits each possible model in proportion to its posterior probability; the Bayesian model averaged parameter estimate is simply the average of the parameter over all MCMC iterations. The relative importance of each predictor variable was given by the posterior probability of inclusion for each variable [Pr(*p* > 0)]. The prior probability that *p* was greater than zero was 0.5, so posterior probabilities of variable inclusion greater than 0.5 indicate evidence in favour of variable inclusion, with values greater than 0.75 providing strong evidence for variable inclusion (odds ratio greater than 3).

Five-fold cross-validation was used to estimate the predictive capacity of the fitted model and to test whether the model is likely to be identifying true relationships. Cross-validation involves splitting the data into ‘training’ and ‘test’ datasets, with a model fitted to the training data used to predict the test data, which were not included in model fitting. This process was repeated five times, with approximately 20% of the data used as a test dataset and the remaining 80% of the data used in model fitting. A different test dataset was used each time, so that all individuals were included in one test dataset.

Individual genetic diversity of individuals within the same historically distinct populations can be correlated due to shared population history. To account for possible correlations between population history and HL, cross-validation test datasets comprised complete population clusters, with approximately 120 individuals in each test dataset (1–5 population clusters). There were a total of nine population clusters (Wollemi+ Wheeny, Glenbrook+ Erskine+ Kowmung+ Little, Lachlan+ Abercrombie, Adjungbilly+ Murrumbidgee, Cotter, Dartmouth+ Hollands, Sevens, Hughes and King Parrot, based on most likely clusters from K=12 STRUCTURE analysis, Appendix 11). We used microsatellite clusters, rather than phylogenetic groups, because recent history (i.e. drift in small populations) could have a strong effect on HL, which can differ in distinct genetic clusters. Model predictions for cross-validation are based on environmental variables only; random effects (basin and site) are used in model fitting but are not used to make predictions.

WinBUGS model code

Model{

for (i in 1:N) {

# response is normally distributed with mean mu and precision tau

y[i] ~ dnorm(mu[i], tau)

# the mean mu is a linear or nonlinear function of covariates and

# random intercepts for basin and site

mu[i] <- alpha1 + mu.cov[i] + rand.site[site[i]] + rand.basin[basin[i]]

# covariate effects are calculated below but summed here for

# inclusion in calculation of mu

mu.cov[i] <- sum(cov.in[i, ])

# apply a prior to predictor variables to account for missing data (NAs)

for (c in 1:Q) {

X[i, c] ~ dnorm(0, 1)

Xc[i, c] <- cut(X[i, c])

# calculate covariate effects

cov.in[i, c] <- sum(cov.in.seg[, i, c])

for (j in 1:10) {

cov.in.seg[j, i, c] <- step(Xc[i, c] - xdum[(j + 1), c]) * beta[j, c] * (Xc[i, c] - xdum[(j + 1), c])

}

}

# monitor nodes needed to estimate mean linear slope of the (possibly non-linear) splines

for (c in 1:Q) {

Xc.res[i, c] <- Xc[i, c] - mean(Xc[, c])

Xc.res.sq[i, c] <- pow(Xc.res[i, c], 2)

slope.num[i, c] <- (cov.in[i, c] - mean(cov.in[, c])) * Xc.res[i, c]

}

}

# priors for intercept and precision

alpha1 ~ dnorm(0, 0.001)

tau ~ dgamma(0.001, 0.001)

# exchangeable priors for random effects – these sum to zero

for (i in 1:(n.site - 1)) {

rand.site[i] ~ dnorm(0, tau.site)

}

rand.site[n.site] <- -sum(rand.site[1:(n.site - 1)])

tau.site ~ dgamma(0.001, 0.001)

for (i in 1:(n.basin - 1)) {

rand.basin[i] ~ dnorm(0, tau.basin)

}

rand.basin[n.basin] <- -sum(rand.basin[1:(n.basin - 1)])

tau.basin ~ dgamma(0.001, 0.001)

#create vectors from supplied matrix of dummy variables - jump spline functions require separate vector for each variable

for (i in 1:11) {

x1[i] <- xdum[i, 1]

x2[i] <- xdum[i, 2]

x3[i] <- xdum[i, 3]

x4[i] <- xdum[i, 4]

x5[i] <- xdum[i, 5]

x6[i] <- xdum[i, 6]

x7[i] <- xdum[i, 7]

}

# code to identify break points (via reversible jump MCMC) in linear splines for the covariates

#remove or add lines for more or fewer variables

for (i in 1:10) {

beta[i, 1] <- beta1[i+1] - beta1[i]

beta[i, 2] <- beta2[i+1] - beta2[i]

beta[i, 3] <- beta3[i+1] - beta3[i]

beta[i, 4] <- beta4[i+1] - beta4[i]

beta[i, 5] <- beta5[i+1] - beta5[i]

beta[i, 6] <- beta6[i+1] - beta6[i]

beta[i, 7] <- beta7[i+1] - beta7[i]

}

beta1[1:11] <- jump.pw.poly.df.gen(x1[1:11], k[1], beta.prec, 0, 0)

beta2[1:11] <- jump.pw.poly.df.gen(x2[1:11], k[2], beta.prec, 0, 0)

beta3[1:11] <- jump.pw.poly.df.gen(x3[1:11], k[3], beta.prec, 0, 0)

beta4[1:11] <- jump.pw.poly.df.gen(x4[1:11], k[4], beta.prec, 0, 0)

beta5[1:11] <- jump.pw.poly.df.gen(x5[1:11], k[5], beta.prec, 0, 0)

beta6[1:11] <- jump.pw.poly.df.gen(x6[1:11], k[6], beta.prec, 0, 0)

beta7[1:11] <- jump.pw.poly.df.gen(x7[1:11], k[7], beta.prec, 0, 0)

# priors for k[i], no of line segments in each linear spline

for (i in 1:Q) {

k[i] ~ dshifted.cat(pc[], 0) # pc[] supplied as data (must sum to 1)

}

# monitor mean linear slope (effect) and probabilitiy of variable inclusion (inc)

for (c in 1:Q) {

effect[c] <- sum(slope.num[, c]) / sum(Xc.res.sq[, c])

inc[c] <- 1 - equals(k[c], 0)

}

#Half-Cauchy (hyper)prior for s.d. of linear coefficients

beta.prec <- pow(beta.sd, -2)

beta.sd <- abs(hyper.Z) / sqrt(tauZ.eta)

hyper.Z ~ dnorm(0, scalef)

tauZ.eta ~ dgamma(1, 1)

# to plot fitted effects

for (i in 1:n.plot) {

for (c in 1:Q) {

for (j in 1:10) {

cov.plot[j, i, c] <- step(x.plot[i, c] - xdum[(j + 1), c]) * beta[j, c] * (x.plot[i, c] - xdum[(j + 1), c])

}

cov.plot2[i, c] <- sum(cov.plot[, i, c])

}

}

# to predict y in new locations

for (i in 1:n.pred) {

y.pred[i] ~ dnorm(mu.pred[i], tau)

mu.pred[i] <- alpha1 + mu.cov.pred[i]

mu.cov.pred[i] <- sum(cov.pred[i, ])

for (c in 1:Q) {

X.pred[i, c] ~ dnorm(0, 1)

Xc.pred[i, c] <- cut(X.pred[i, c])

# spline code

cov.pred[i, c] <- sum(cov.pred.seg[, i, c])

for (j in 1:10) {

cov.pred.seg[j, i, c] <- step(Xc.pred[i, c] - xdum[(j + 1), c]) * beta[j, c] * (Xc.pred[i, c] - xdum[(j + 1), c])

}

}

}

}

References:

Lunn, D.J., Best, N., Whittaker, J.C., 2009. Generic reversible jump MCMC using graphical models. Statistics and Computing 19, 395-408.

Lunn, D.J., Thomas, A., Best, N., Spiegelhalter, D., 2000. WinBUGS- a Bayesian modelling framework: concepts, structure, and extensibility. Statistics and Computing 10, 325-337.

Lunn, D.J., Whittaker, J.C., Best, N., 2006. A Bayesian toolkit for genetic association studies. Genet Epidemiol 30, 231-247.

Raftery, A.E., Madigan, D., Hoeting, J.A., 1997. Bayesian model averaging for linear regression models. Journal of the American Statistical Association 92, 179-191.

Figure S5A. Fitted relationships between HL and each of the seven environmental variables included in the model. P(inc) is the probability of variable inclusion in the fitted model. The x-axis shows standardized value for each variable (e.g. a value of 1 means 1 standard deviation above the mean value for that variable), the y-axis shows deviation from the mean HL value (in units of HL) for a given standardized value of the predictor variable. Grey shading is one standard deviation of the fitted effect.


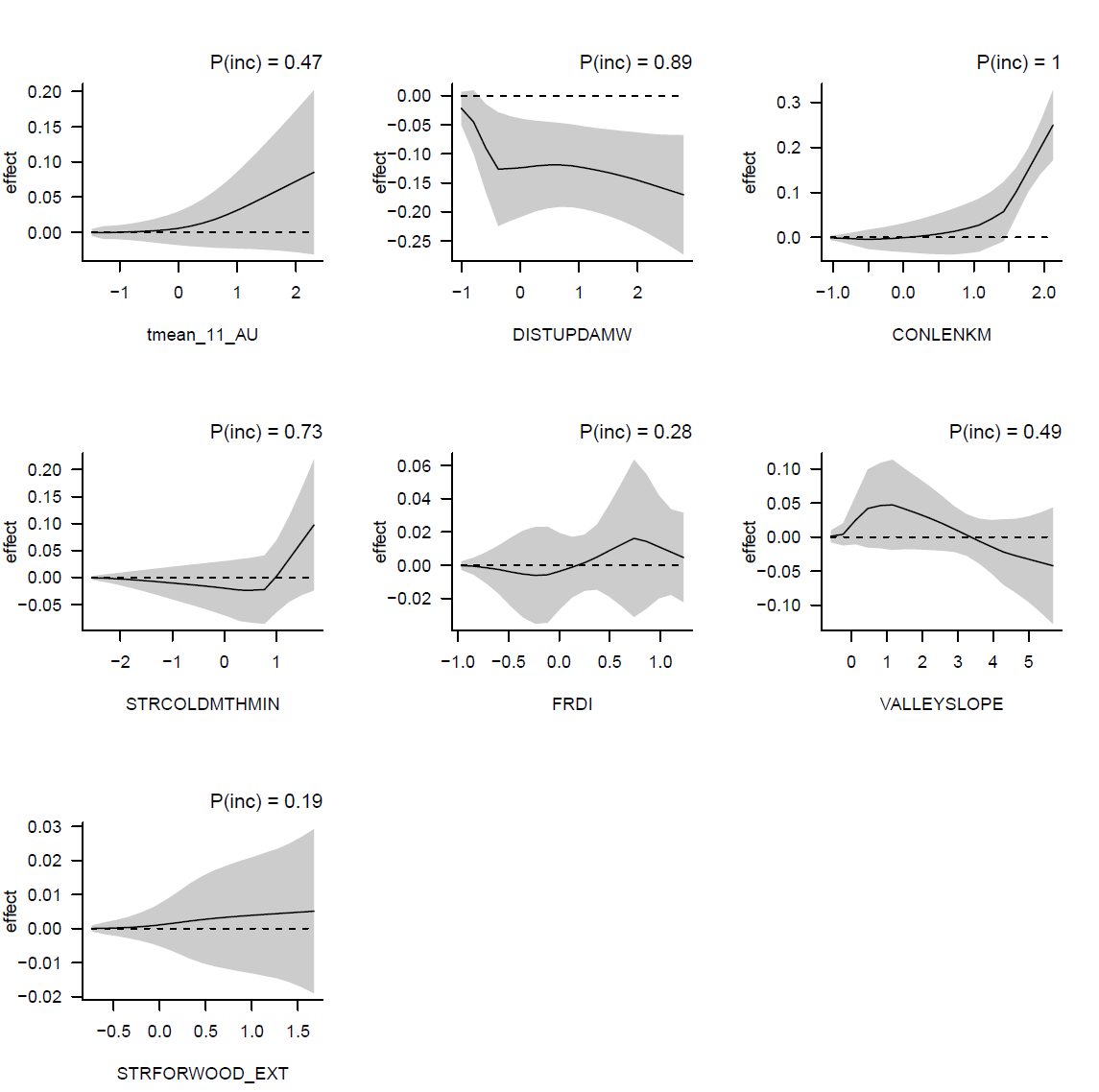


Appendix 6. Vortex simulations of population viability under two management scenarios (do nothing and 50 years of translocations) accounting for genetic factors.

*Simulation parameters.* We assumed that extinction occurs when only one sex remains in a population and that migration and mutation do not occur. Percent adult female breeders was set to 23% to ensure positive population growth in a deterministic model but sensitivity to demographic, environmental and genetic stochasticity dependent on population sizes. The real value is unknown, but preliminary analyses showed that using 10% (thought to be a realistic number for Dartmouth; Zeb Tonkin; unpublished data on acoustic telemetry and mark-recapture) or 20% results in a negative population growth rate in a deterministic model, and 25% (tested by Todd and Lintermans (2015)) results in infinite population growth under most conditions, which contradicts the observed population dynamics.

Initial population size (number of adult individuals of real age 3-26) is also unknown for wild Macquarie perch populations, but sizes tested in our simulations (3000, 500, 300 and 100, modelled based on allele frequencies for Dartmouth, King Parrot, Cataract Dam and Murrumbidgee, respectively) should encompass a range of realistic values. For example, mark-recapture data of the primary spawning site in Dartmouth show that ~1000 (800-1500) adult fish (>350mm and/or 4 years of age (Appleford et al. 1998)) are spawning each year, and these could represent 10-50% of a total adult population (Zeb Tonkin; unpublished acoustic telemetry and mark-recapture data). The number of adult Macquarie perch in King Parrot Creek (>250 mm; smaller size due to lower growth rates compared to Dartmouth) is ~500-3400 individuals based on 10-50% detectability (using 2015-2016 data) and assuming that sample sites are representative of the entire reach; fewer fish >250 mm were collected in 2016 and 2015 compared to 2014 (Joanne Kearns, Renae Ayres; unpublished data). For Upper Murrumbidgee the actual number of adults could be as low as <20-50 (Mark Lintermans; unpublished data). No guestimate for Cataract Dam could be made, but for Cotter Dam 600-1000 adults might be a realistic value.

Genetic results were summarized for 19 microsatellite and 1 mitochondrial markers simulated based on observed allele frequencies (option “additional loci only”). Other simulation parameters are presented in Table S6A. Graphical representation of results for all simulations is in Fig. S6A

Table S6A. Additional parameters for Vortex simulations. Due to limited range of fecundities which could be assigned to adult females in Vortex, all simulations were restricted to adult fish (e.g. simulations assumed that the fish of age zero (=real age 3) produces offspring of adult fish in 3 years), thus Vortex age =real age-3.

| Input variable | Value | Description | Comments | Supporting reference |
| --- | --- | --- | --- | --- |
| *Species description* |  |  |  |  |
| Inbreeding depression | Checked box |  |  |  |
| Lethal equivalents | 6.29 (Default) | Severity of inbreeding depression | Based on combined effect of inbreeding on fecundity and first year survival | O'Grady et al. (2006) |
| % of inbreeding depression that is due to recessive lethals | 50 (Default) | % of inbreeding depression that is due to lethal alleles vs. other genetic mechanisms | Based on studies on Drosophila | Simmons and Crow (1977) |
| Environmental correlation between reproduction and survival | 0.5 (Default) | Extent to which environmental variation in reproduction and survival are synchronized | Spawning condition and spawning success are associated with environmental conditions | Gray et al. (2000), Ingram and Gooley (1996)  Tonkin et al. (2014), Tonkin et al. (2015a), Tonkin et al. (2015b) |
| Environmental correlation among populations | 0.5 (Default) | Extent to which populations are subjected to synchronous environmental variation in reproduction and survival rates | Spawning aggregations could be environmentally cued, based on multi-population assessment of recruitment dynamics across Victoria | Cadwallader and Rogan (1977), Tonkin et al. (2015b) |
| *Reproductive system* |  |  |  |  |
| System | Polygamy | In Vortex- polygyny, with new selection of mates each year | Form annual spawning aggregations | Cadwallader and Rogan (1977), Tonkin et al. (2014) |
| Age of first (mature) offspring -females | 3 | As the first 3 years of life (egg to 3-year-old) are ignored by simulations, Vortex age 3 represents 6-years-old fish: the first age at which adult offspring is produced | Majority of breeding females are 3+ years old | Appleford et al. (1998), Koehn and O'Connor (1990) |
| Age of first (mature) offspring -males | 3 | As above | Majority of breeding males are 3+ years old | Appleford et al. (1998), Koehn and O'Connor (1990) |
| Max lifespan | 23 |  | Oldest fish aged 26 | Lintermans and Ebner (2010) |
| Max age female reproduction | 23 |  | Oldest female fish collected from spawning aggregation aged at 26 | Tonkin et al. (2014), DEPI unpublished data (ARI/Snobs Creek Hatchery) |
| Max age male reproduction | 23 |  | Oldest male fish collected from spawning aggregation aged at 26 | Tonkin et al. (2014), DEPI unpublished data (ARI/ Snobs Creek Hatchery) |
| Max number broods per year | 1 |  | Annual spawning fish. Females are presumed to spawn all mature eggs at one time. | Appleford et al. (1998), Cadwallader and Rogan (1977) |
| Max (adult) progeny per brood | 10 | Offspring that survived to adulthood (3-year-old) | Max fecundity ~110,000 eggs/female. Probability of survival from egg to a mature 3-year-old fish is estimated at ~9.3x10^-5^ = 0.5 (egg hatching success) x 0.013 (larval survival) x 0.13 (survival post-larvae to 1-year-old) x 0.25 (survival from 1 to 2-year-old) x 0.44 (survival from 2 to 3 year old) | Cadwallader and Rogan (1977), Table 1 and Fig. 5 of Todd and Lintermans (2015) |
| Sex ratio at birth (in % males) | 50 |  | Presumed to be close to 50, but may be influenced by environmental conditions during embryonic development | Ospina-Alvarez and Piferrer (2008), Penman and Piferrer (2008) |
| *Reproductive rates* |  |  |  |  |
| % adult females breeding | 23 |  | Limited data | Zeb Tonkin (unpublished data), Todd and Lintermans (2015) |
| SD in % breeding due to environmental variation | 5 |  | As above |  |
| % adult males in the pool of breeders | 100 |  |  |  |
| Number of broods per year (as %) | 1 brood – 100% | Distribution is for number of females breeding | One brood per year | Appleford et al. (1998), Cadwallader and Rogan (1977) |
| Number of adult offspring per female per brood | Normal distribution, mean=7, SD=2 | Average number of adult (3-year-old) offspring produced each year by each female | Fecundity is age-dependant (increases almost linearly from ~20,000 to ~85,000 at age 13, then stabilizes at ~90,000 at age16). Here, fecundity is approximated as normal distribution with mean number of eggs per brood per female=76,000 and sd=22,000 assuming survival from egg to a mature fish of ~9.3x10^-5^ (above) | Fig. 5 of Todd and Lintermans (2015) |
| *Mortality rates* |  |  |  |  |
| Mortality age 0-1 | 44% | Mortality for 3-year-old to 4-year-old | Mean probability of survival from 3-year old to 4-year old is 0.56 (SD=0.11) | Table 1 of Todd and Lintermans (2015) |
| SD in 0-1 mortality due to environmental variation | 11% |  | As above | Table 1 of Todd and Lintermans (2015) |
| Mortality age 1-2 | 37% | Mortality for 4-year-old to 5-year-old | Mean probability of survival from 4-year old to 5-year old is 0.63 (SD=0.10) | Table 1 of Todd and Lintermans (2015) |
| SD in 1-2 mortality due to environmental variation | 10% |  | As above | Table 1 of Todd and Lintermans (2015) |
| Mortality age 2-3 | 31% | Mortality after 5-year-old to 6-year-old | Average mean probability of survival from 5-year old to 6-year old is 0.69 (SD=0.10) | Table 1 of Todd and Lintermans (2015) |
| SD in 2-3 mortality due to environmental variation | 10% |  | As above | Table 1 of Todd and Lintermans (2015) |
| Mortality age >3 | 19% | Mortality after 6-year-old | Average mean probability of survival from 5-year old to 26-year old is 0.81 | Table 1 of Todd and Lintermans (2015) |
| SD in >3 mortality due to environmental variation | 8% |  | Average standard deviation of probability of survival from 5-year old to 26-year old is 0.08 | Table 1 of Todd and Lintermans (2015) |
| *Initial population size* |  |  |  |  |
| Dartmouth N  King Parrot N | 3000  500 | A total number of adult fish (Vortex age 3 to 23) |  |  |
| Cataract Dam N  Murrumbidgee N | 300  100 |  |  |  |
| Age distribution | Use stable age distribution | Automatically calculated based on birth and death rates |  |  |
| *Carrying capacity (K)* |  |  |  |  |
| *K* (SD in K) for Dartmouth | 30,000 (3,000) | Carrying capacity (i.e. max number of adults allowed) and SD due to environmental variation |  |  |
| *K* (SD in K) for King Parrot | 3,000 (300) |  |  |  |
| *K* (SD in K) for Cataract Dam | 30,000 (3,000) |  |  |  |
| *K* (SD in K) for Murrumbidgee | 3,000 (300) |  |  |  |
| *Harvest- implement as translocation* |  |  |  |  |
| Percent survival during translocation | 100 | All translocated individuals survive |  |  |
| First year of harvest | 1 | Start translocations in year 1 |  |  |
| Last year of harvest | 50 | End translocations in year 50 |  |  |
| Interval between harvests | 1 | Do translocations every year from year 1 to year 50 |  |  |
| Number of adult females to be harvested | 3 (one each of Vortex age 1, 2 and 3) | Translocate 3 mature females from larger to smaller population in a pair (Dartmouth to King Parrot or Cataract Dam to Murrumbidgee) |  |  |
| Number of adult males to be harvested | 3 (one each of Vortex age 1, 2 and 3) | Translocate 3 mature males from larger to smaller population in a pair |  |  |

Figure S6A. Results of Vortex simulations for two pairs of (large and small) populations. Left panel: Dartmouth (initial population size N=3000) and King Parrot (N=500). Right panel: Cataract Dam (N=300) and Murrumbidgee (N=100). Blue and red lines show results for do-nothing scenario. Green and purple lines show results of 50 years of translocations scenario, where small population (purple line) is supplemented with 6 adults (3 of each sex) from a large population (green) to a small (purple) population every year for 50 years. Top three panels show results for (A) mean probability of survival, (B) mean N for all extant populations and (C) mean genetic diversity (heterozygosity at modelled loci).


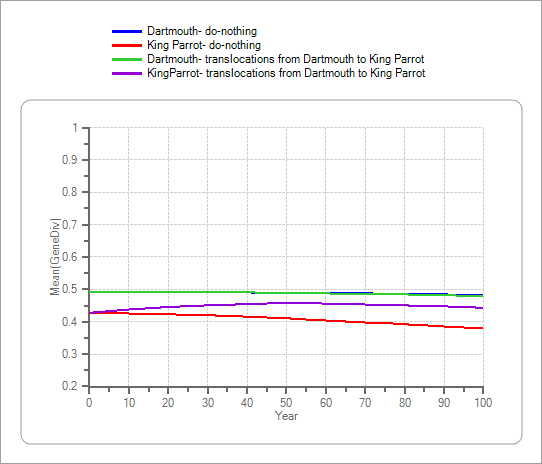

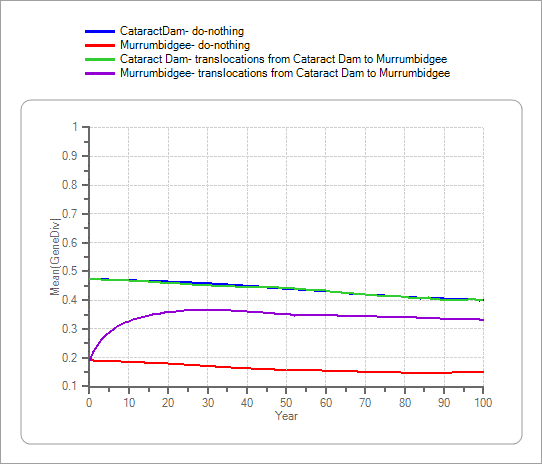

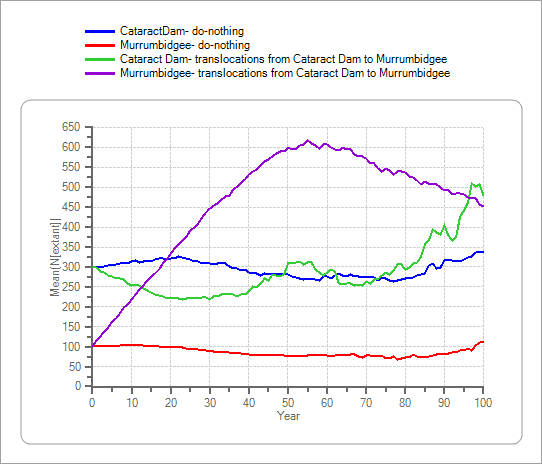

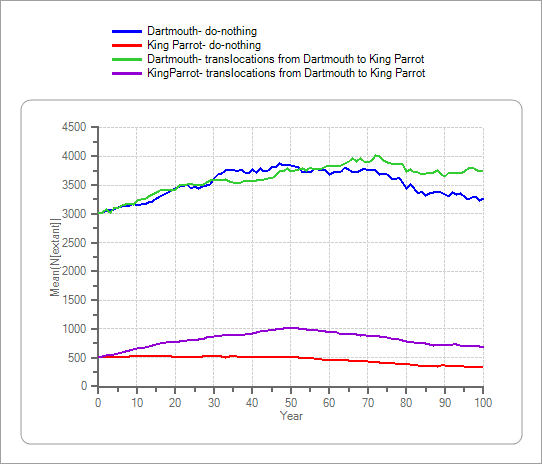

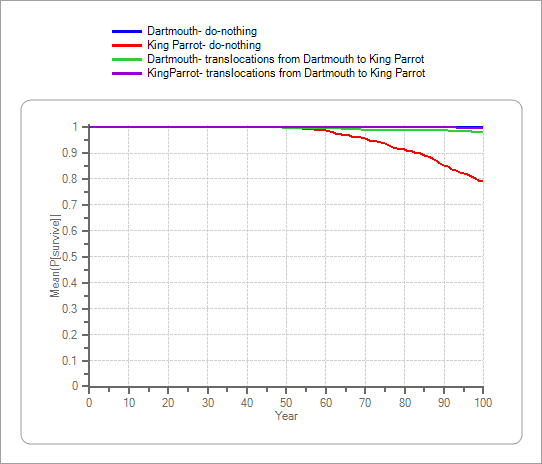

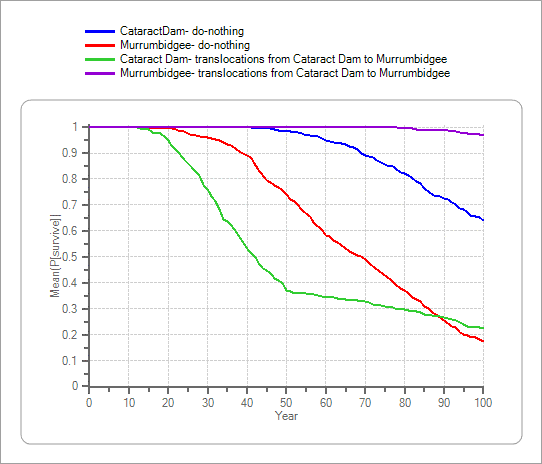


A

B

C

Figure S6A (continued). Left panel: Dartmouth (initial population size N=3000) and King Parrot (N=500). Right panel: Cataract Dam (N=300) and Murrumbidgee (N=100). Blue and red lines show results for do-nothing scenario. Green and purple lines show results of 50 years of translocations scenario, where small population (purple line) is supplemented with 6 adults (3 of each sex) from a large population (green) to a small (purple) population every year for 50 years. Bottom three panels show results for (D) mean inbreeding (homozygosity at modelled loci), (E) mean number of alleles and (F) mean number of lethal alleles per individual.


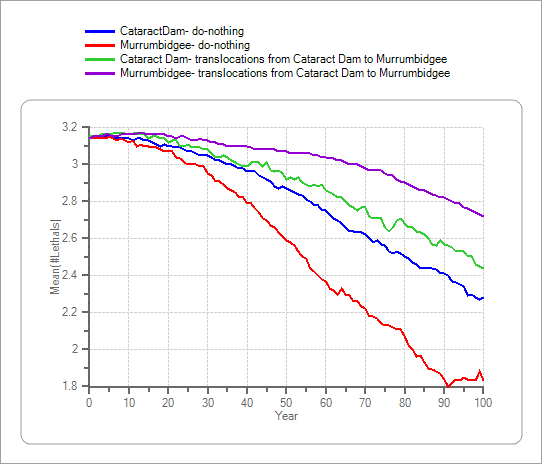

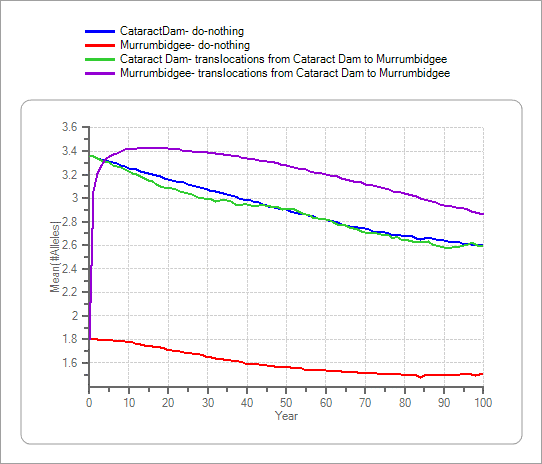

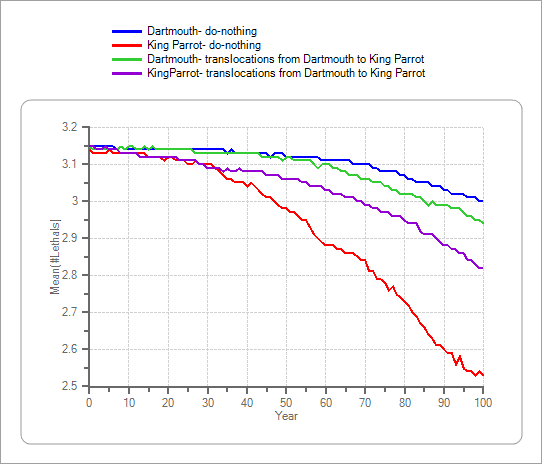

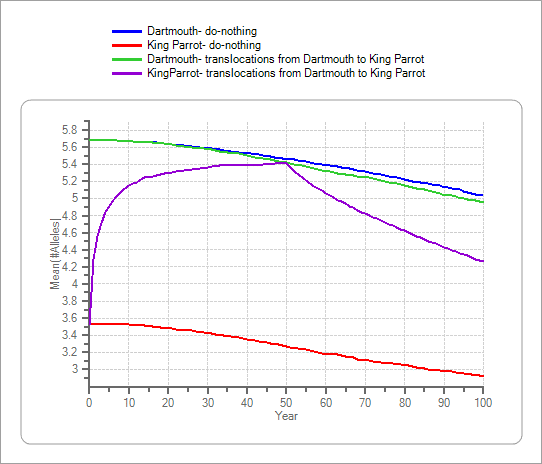

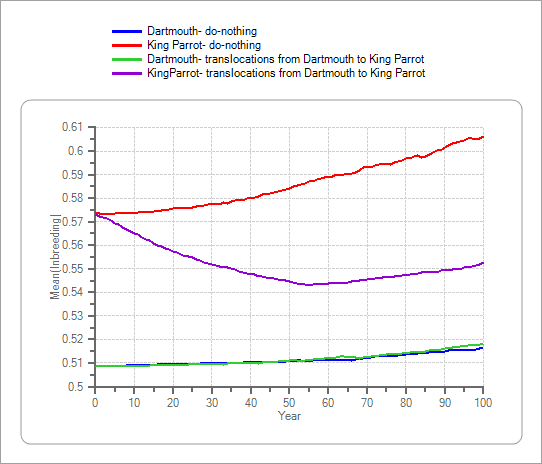

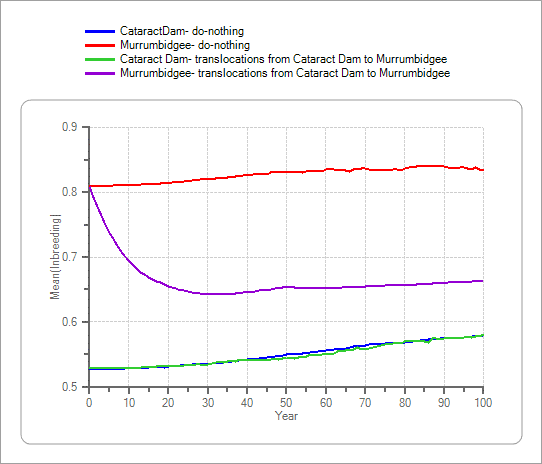


D

E

F

References:

Appleford, P., T. Anderson, and G. Gooley. 1998. Reproductive cycle and gonadal development of Macquarie perch, *Macquaria australasica* Cuvier (Percichthyidae), in Lake Dartmouth and tributaries of the Murray–Darling Basin, Victoria, Australia. Mar Freshw Res 49:163-169.

Cadwallader, P. and P. Rogan. 1977. The Macquarie perch, *Macquria australasica* (Pisces: Percichthyidae), of Lake Eildon, Victoria. Aust J Ecol 2:409-418.

Gray, S. C., S. S. De Silva, B. A. Ingram, and G. J. Gooley. 2000. Effects of river impoundment on body condition and reproductive performance of the Australian native fish, Macquarie perch (Macquaria australasica). Lakes & Reservoirs: Research & Management 5:281-291.

Ingram, B. and G. Gooley. 1996. Hormone induced spawning of the threatened Macquarie perch (*Macquaria australasica*): an Australian native freshwater fish. Pp. 97 *in* D. A. Hancock, and J. P. Beumer, eds. Developing and Sustaining World Fisheries Resources: The State of Science and Management. Proceedings Volume 1.

Koehn, J. and W. O'Connor. 1990. Biological information for management of native freshwater fish in Victoria. Department of Conservation and Environment, Freshwater Fish Management Branch, Arthur Rylah Institute for Environmental Research.

Lintermans, M. and B. Ebner. 2010. Threatened Fish Profile: ‘Western’ Macquarie perch Macquaria australasica Cuvier 1830. Australian Society for Fish Biology Newsletter 40(2): 76-78.

O'Grady, J. J., B. W. Brook, D. H. Reed, J. D. Ballou, D. W. Tonkyn, and R. Frankham. 2006. Realistic levels of inbreeding depression strongly affect extinction risk in wild populations. Biol Conserv 133:42-51.

Ospina-Alvarez, N. and F. Piferrer. 2008. Temperature-dependent sex determination in fish revisited: prevalence, a single sex ratio response pattern, and possible effects of climate change. PLoS One 3:e2837.

Penman, D. J. and F. Piferrer. 2008. Fish gonadogenesis. Part I: genetic and environmental mechanisms of sex determination. Rev Fish Sci 16:16-34.

Simmons, M. J. and J. F. Crow. 1977. Mutations affecting fitness in Drosophila populations. Annu Rev Genet 11:49-78.

Todd, C. R. and M. Lintermans. 2015. Who do you move? A stochastic population model to guide translocation strategies for an endangered freshwater fish in south-eastern Australia. Ecol Model 311:63-72.

Tonkin, Z., J. Kearns, J. Mahoney, and J. Mahony. 2015a. Spatio-temporal spawning patterns of two riverine populations of the threatened Macquarie perch *Macquaria australasica*. Mar Freshw Res Published online: 23 November 2015; http://dx.doi.org/10.1071/MF15319.

Tonkin, Z., J. Kearns, J. O’Mahony, J. Mahoney, A. Kitchingman, and R. Ayres. 2015b. Sustaining Macquarie perch in the Yarra River – a multipopulation investigation of recruitment dynamics. Arthur Rylah Institute for Environmental Research, unpublished client report for Melbourne Water. Department of Environment, Land, Water and Planning, Heidelberg, Victoria.

Tonkin, Z., J. Lyon, D. S. Ramsey, N. R. Bond, G. Hackett, K. Krusic-Golub, B. A. Ingram, and S. R. Balcombe. 2014. Reservoir refilling enhances growth and recruitment of an endangered remnant riverine fish. Can J Fish Aquat Sci 71:1888–1899.

Appendix 7. Distribution of the mitochondrial control region haplotypes across 338 Macquarie perch individuals from 19 populations. Hawkesbury-Nepean Basin (HNB) haplotypes are shaded blue, Murray-Darling Basin (MDB) are not shaded. Haplotype numbers as on the network on Fig. 1 of the main text, haplotypes fixed for a population are in bold. A single unique Shoalhaven Basin haplotype (Kangaroo River, KR1) is not included in this table.

| **Haplotype number** | **Sample size** | **Wheeny** | **Glenbrook** | **Little** | **Cataract_River** | **Cataract_Dam** | **Abercrombie** | **Lachlan** | **Adjungbilly** | **Cotter** | **Murrumbidgee** | **Dartmouth** | **Hollands** | **Sevens** | **Hughes** | **King Parrot** | **Yarra** | **List of samples** |
| --- | --- | --- | --- | --- | --- | --- | --- | --- | --- | --- | --- | --- | --- | --- | --- | --- | --- | --- |
| Hap_1 | 10 |  |  |  |  |  | **6** | 4 |  |  |  |  |  |  |  |  |  | AB12 AB15 AB17 AB40 AB41 LN22 LN23 LN24 LN25 TU14 |
| Hap_2 | 19 |  |  |  |  |  |  |  | 19 |  |  |  |  |  |  |  |  | AC1 AC10 AC11 AC12 AC14 AC15 AC16 AC17 AC18 AC19 AC2 AC20 AC22 AC3 AC4 AC5 AC6 AC7 AC8 |
| Hap_3 | 1 |  |  |  |  |  |  |  | 1 |  |  |  |  |  |  |  |  | AC13 |
| Hap_4 | 2 |  |  |  |  |  |  |  | 2 |  |  |  |  |  |  |  |  | AC21 AC23 |
| Hap_5 | 1 |  |  |  |  |  |  |  | 1 |  |  |  |  |  |  |  |  | AC9 |
| Hap_6 | 19 |  |  |  |  |  |  |  |  |  |  |  | 1 |  |  |  | 18 | B1 G039 G041 G043 G158 G174 G18 G186 G191 G233 G235 GBR01 GBR02 GBR06 GBR08 GBR09 GBR19 GBR22 GBR27 |
| Hap_7 | 20 |  |  |  |  |  |  |  |  |  |  | 7 | 3 | 10 |  |  |  | B2 B25 B7 C117 C29 C54 CBR04 CBR07 CBR14 CBR25 F31 F34 F36 F37 F38 F40 F41 F42 F44 F45 |
| Hap_8 | 6 |  |  |  |  |  |  |  |  |  |  |  | 3 |  |  |  | 3 | B23 B26 B29 G040 G184 G188 |
| Hap_9 | 6 |  |  |  |  |  |  |  |  |  |  | 5 | 1 |  |  |  |  | B24 C01 C07 C21 CBR15 CBR23 |
| Hap_10 | 80 |  |  |  | 10 | 9 |  |  |  | **15** |  | 26 | 2 | 2 | 2 |  | 14 | B27 B8 C02 C03 C103 C118 C131 C13 C161 C163 C164 C177 C178 C181 C187 C25 C35 CBR02 CBR08 CBR09 CBR10 CBR11 CBR16 CBR17 CBR18 CBR20 CBR22 CBR24 CDM01 CDM03 CDM05 CDM07 CDM08 CDM09 CDM10 CDM13 CDM14 CR22 CR27 CR28 CR31 CR40 CR41 CR42 CR60 CR64 CR71 E1 E2 F35 F39 G011 G147 G164 G19 G190 G192 G232 G237 GBR03 GBR04 GBR10 GBR18 GBR20 GBR26 L31 L32 L33 L61 L62 L63 L88 N01 N02 N03 N04 N05 N21 N22 N23 |
| Hap_11 | 7 |  |  |  |  |  |  |  |  |  |  |  | 1 |  |  | 6 |  | B28 D10 D12 D15 D16 D2 D3 |
| Hap_12 | 12 |  |  |  |  |  |  |  |  |  |  | 8 | 4 |  |  |  |  | B3 B4 B5 B6 C04 C180 C185 C37 CBR01 CBR03 CBR13 CBR19 |
| Hap_13 | 4 |  |  |  |  |  |  |  |  |  |  | 3 |  |  |  |  | 1 | C05 C110 C183 G146 |
| Hap_14 | 5 |  |  |  |  |  |  |  |  |  |  | 3 |  | 1 |  |  | 1 | CBR06 CBR12 CBR21 F32 G234 |
| Hap_15 | 24 |  |  |  | 10 | 6 |  |  |  |  |  |  |  | 2 | 4 |  | 2 | CDM02 CDM04 CDM06 CDM11 CDM12 CDM15 CR32 CR37 CR39 CR47 CR49 CR51 CR52 CR68 CR69 CR74 E10 E14 E7 E9 F33 F43 G163 GBR13 |
| Hap_16 | 32 |  |  |  | 32 |  |  |  |  |  |  |  |  |  |  |  |  | CR21 CR23 CR24 CR25 CR26 CR29 CR30 CR33 CR34 CR35 CR36 CR38 CR43 CR44 CR45 CR46 CR48 CR50 CR53 CR54 CR55 CR56 CR57 CR58 CR59 CR61 CR62 CR63 CR66 CR67 CR72 CR73 |
| Hap_17 | 1 |  |  |  | 1 |  |  |  |  |  |  |  |  |  |  |  |  | CR65 |
| Hap_18 | 1 |  |  |  | 1 |  |  |  |  |  |  |  |  |  |  |  |  | CR70 |
| Hap_19 | 7 |  |  |  |  |  |  |  |  |  |  |  |  |  |  | 7 |  | D11 D13 D14 D4 D6 D8 D9 |
| Hap_20 | 1 |  |  |  |  |  |  |  |  |  |  |  |  |  |  | 1 |  | D5 |
| Hap_21 | 1 |  |  |  |  |  |  |  |  |  |  |  |  |  |  | 1 |  | D7 |
| Hap_22 | 5 |  |  |  |  |  |  |  |  |  |  |  |  |  | 5 |  |  | E11 E15 E4 E6 E8 |
| Hap_23 | 3 |  |  |  |  |  |  |  |  |  |  |  |  |  | 3 |  |  | E12 E13 E3 |
| Hap_24 | 1 |  |  |  |  |  |  |  |  |  |  |  |  |  | 1 |  |  | E5 |
| Hap_25 | 2 |  |  |  |  |  |  |  |  |  |  |  |  |  |  |  | 2 | G007 GBR12 |
| Hap_26 | 8 |  |  |  |  |  |  |  |  |  |  |  |  |  |  |  | 8 | G009 G152 G159 G160 G179 G236 GBR05 GBR14 |
| Hap_27 | 4 |  |  |  |  |  |  |  |  |  |  |  |  |  |  |  | 4 | G010 G042 G182 G183 |
| Hap_28 | 4 |  |  |  |  |  |  |  |  |  |  |  |  |  |  |  | 4 | G038 G17 G185 G231 |
| Hap_29 | 1 |  |  |  |  |  |  |  |  |  |  |  |  |  |  |  | 1 | G153 |
| Hap_30 | 1 |  |  |  |  |  |  |  |  |  |  |  |  |  |  |  | 1 | G175 |
| Hap_31 | 4 |  |  |  |  |  |  |  |  |  |  |  |  |  |  |  | 4 | G32 GBR23 GBR24 GBR25 |
| Hap_32 | 11 |  | **11** |  |  |  |  |  |  |  |  |  |  |  |  |  |  | GB16 GB17 GB18 GB19 GB23 GB26 GB27 GB28 GB33 GB34 GB35 |
| Hap_33 | 1 |  |  |  |  |  |  |  |  |  |  |  |  |  |  |  | 1 | GBR07 |
| Hap_34 | 3 |  |  |  |  |  |  |  |  |  |  |  |  |  |  |  | 3 | GBR11 GBR15 GBR16 |
| Hap_35 | 1 |  |  |  |  |  |  |  |  |  |  |  |  |  |  |  | 1 | GBR17 |
| Hap_36 | 1 |  |  |  |  |  |  | 1 |  |  |  |  |  |  |  |  |  | LN21 |
| Hap_37 | 2 |  |  | 2 |  |  |  |  |  |  |  |  |  |  |  |  |  | LT20 LT31 |
| Hap_38 | 3 |  |  | 3 |  |  |  |  |  |  |  |  |  |  |  |  |  | LT21 LT23 LT25 |
| Hap_39 | 3 |  |  | 3 |  |  |  |  |  |  |  |  |  |  |  |  |  | LT22 LT24 LT32 |
| Hap_40 | 2 |  |  | 2 |  |  |  |  |  |  |  |  |  |  |  |  |  | LT29 LT30 |
| Hap_41 | 11 |  |  |  |  |  |  |  |  |  | **11** |  |  |  |  |  |  | M11 M12 M13 M14 M15 M16 M17 M18 M19 M20 M26 |
| Hap_42 | 7 | **7** |  |  |  |  |  |  |  |  |  |  |  |  |  |  |  | WC1 WC12 WC13 WC14 WC15 WC16 WC2 |
| Hap_43 | 1 |  |  | 1 |  |  |  |  |  |  |  |  |  |  |  |  |  | LT9 |
| Total | 338 | 7 | 11 | 11 | 54 | 15 | 6 | 5 | 23 | 15 | 11 | 52 | 15 | 15 | 15 | 15 | 68 |  |
| Population code | | WC | GB | LT | CR | CD, CDM | AB, TU | LN, BC | AC | L, N | M | C, CBR | B | F | E | D | G, GBR |  |

Appendix 8. Maximum clade credibility tree from BEAST analysis of mitochondrial control region sequences, built and summarized in TREEANNOTATOR (Drummond and Rambaut 2007) from tree files combined in LOGCOMBINER, and visualized in FIGTREE; 43 haplotypes from HNB and MDB are labelled as on Fig. 1 and Appendix 7; KR- the Shoalhaven Basin haplotype (Kangaroo River). Size of circles at nodes indicate node support, the numbers by the nodes indicate Bayesian posterior probability and geometric mean [95% HPD] for time of origin (in thousands of years, KY). The time scale below the tree is in million years. Two geographically localized clades (Adjungbilly and Abercrombie+Lachlan) are marked with boxes.

**HNB**

**MDB**

1; 1061 [204-2840] KY

1; 264 [59-674] KY

1; 90 [16-240] KY

0.85; 177 [45-464] KY

Abercrombie/

Lachlan

1; 27 [2-88] KY

Adjungbilly

0.83; 36 [8-100] KY


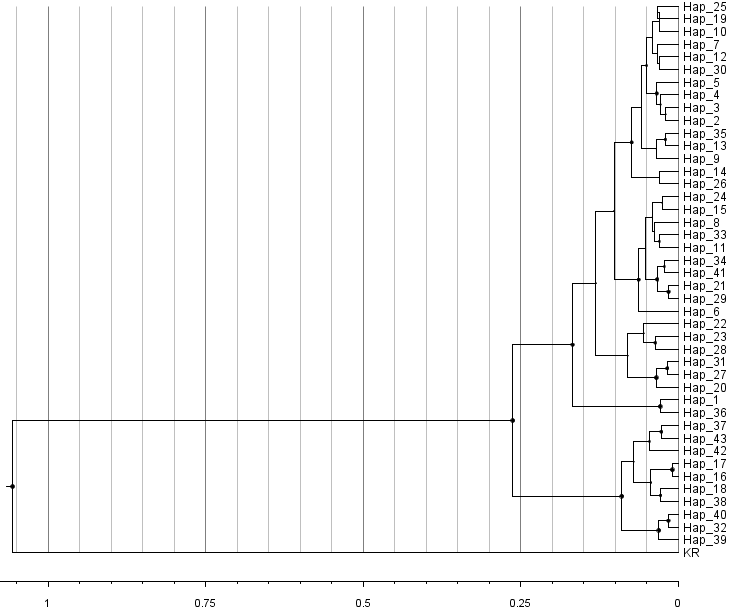


Appendix 9. Analysis of the geographic distribution of microsatellite allele frequencies (Table S9A).

Analysis of the geographic distribution of frequencies of 257 microsatellite alleles (Table S9A) showed that 35% (91) of alleles were restricted to the HNB, 47% (120) to the MDB, 1.6% (4) to Shoalhaven Basin, and 16% (42) were shared among two or more basins. Of 12 MDB populations (including MDB translocations) eight displayed polymorphism at all 19 loci and four (Adjungbilly, Cotter, Murrumbidgee and King Parrot) were polymorphic only for 11-15 microsatellite loci (Fig. 2, Appendix 2). In contrast, all six endemic HNB populations were polymorphic for only 6-16 loci (admixed Cataract River had 19). The Shoalhaven (Kangaroo River, from the divergent lineage presumed extinct) individual was homozygous at 17 of 19 loci; it shared alleles at six loci with both the HNB and MDB, alleles at five loci with the HNB only, and alleles at four loci with the MDB only (four loci had private alleles; Table S9A). Seven HNB populations, six MDB populations and Yarra had 52 private alleles at 17 loci, of which 63% (33 alleles) were in HNB populations, 23% (12) in MDB, and 13% (7) in Yarra. Despite smaller sample sizes in the HNB, mean number of private alleles per locus was higher in the HNB than in the MDB (Appendix 2).

Table S9A. Geographic distribution of frequencies of 257 alleles of 19 microsatellite loci screened for 871 Macquarie perch individuals from 20 populations. Private alleles are underlined, basins are separated by a vertical line (Cataract River has a mixture of HNB and MDB genotypes; Cataract Dam is in HNB but is a translocated population from MDB; Yarra River is in Yarra River Basin but is a translocated population from MDB). Alleles restricted to HNB are shaded blue (N=91), those restricted to MDB (N=120) are shaded orange, private to Shoalhaven Basin (N=4) are shaded yellow, those shared among basins are left unshaded. Of all microsatellite alleles, 35% were restricted to the HNB, 47% to the MDB, 1.6% to Shoalhaven Basin, and the rest were shared among two or three basins. Despite smaller sample sizes in the HNB, mean number of private alleles per population was higher in the HNB than in the MDB (Appendix 2).

| **Locus** | **Allele/ sample size** | **Wollemi Creek** | **Wheeny Creek** | **Glenbrook Creek** | **Erskine Creek** | **Kowmung River** | **Little River** | **Cataract River** | **Cataract Dam** | **Abercrombie River** | **Lachlan River** | **Adjungbilly Creek** | **Cotter River** | **Murrumbidgee River** | **Dartmouth Lake** | **Hollands Creek** | **Sevens Creek** | **Hughes Creek** | **King Parrot Creek** | **Yarra River** | **Kangaroo River** |
| --- | --- | --- | --- | --- | --- | --- | --- | --- | --- | --- | --- | --- | --- | --- | --- | --- | --- | --- | --- | --- | --- |
| **AB009** | **N** | 16 | 20 | 30 | 18 | 24 | 25 | 64 | 57 | 31 | 25 | 23 | 127 | 30 | 121 | 27 | 30 | 26 | 29 | 136 | 1 |
|  | **326** |  |  |  |  |  |  | 0.023 | 0.105 | 0.452 | 0.34 | 0.391 |  |  | 0.017 | 0.019 | 0.167 | 0.135 |  | 0.272 |  |
|  | **328** | 0.063 |  |  |  |  |  |  |  |  |  |  |  |  |  |  |  |  |  |  |  |
|  | **329** | 0.844 |  | 1 | 0.806 | 0.479 | 0.94 |  |  |  |  |  |  |  |  |  |  |  |  |  |  |
|  | **330** | 0.094 | 1 |  | 0.194 | 0.521 | 0.06 | 0.977 | 0.895 | 0.548 | 0.66 | 0.609 | 1 | 1 | 0.983 | 0.981 | 0.833 | 0.865 | 1 | 0.728 | 1 |
| **LFMP003** | **N** | 16 | 20 | 30 | 19 | 24 | 25 | 65 | 58 | 31 | 25 | 23 | 129 | 30 | 122 | 30 | 30 | 27 | 30 | 136 | 1 |
|  | **209** |  |  |  | 0.026 |  | 0.04 |  |  |  |  |  |  |  |  |  |  |  |  |  |  |
|  | **213** |  |  | 1 | 0.526 | 0.542 | 0.26 |  |  |  |  |  |  |  |  |  |  |  |  |  |  |
|  | **215** |  |  |  |  | 0.021 |  |  |  |  |  |  |  |  |  |  |  |  |  |  |  |
|  | **217** |  |  |  |  |  | 0.1 | 0.015 |  |  |  |  |  |  |  |  |  |  |  |  |  |
|  | **219** | 1 | 1 |  | 0.447 | 0.438 | 0.6 | 0.015 |  |  | 0.02 |  |  |  |  |  |  |  |  |  |  |
|  | **223** |  |  |  |  |  |  |  |  |  |  |  |  |  | 0.033 | 0.017 |  |  |  | 0.055 | 1 |
|  | **225** |  |  |  |  |  |  | 0.385 |  |  |  |  |  |  |  |  |  |  |  |  |  |
|  | **231** |  |  |  |  |  |  | 0.208 | 0.267 | 0.806 | 0.78 | 0.543 | 0.845 | 0.933 | 0.533 | 0.5 | 0.433 | 0.778 | 0.6 | 0.456 |  |
|  | **233** |  |  |  |  |  |  | 0.354 | 0.69 | 0.194 | 0.2 | 0.457 | 0.155 | 0.067 | 0.094 | 0.15 | 0.417 | 0.019 | 0.283 | 0.375 |  |
|  | **235** |  |  |  |  |  |  | 0.008 | 0.026 |  |  |  |  |  | 0.25 | 0.317 | 0.15 | 0.111 | 0.05 | 0.022 |  |
|  | **237** |  |  |  |  |  |  | 0.015 | 0.017 |  |  |  |  |  |  |  |  | 0.093 |  |  |  |
|  | **241** |  |  |  |  |  |  |  |  |  |  |  |  |  |  |  |  |  | 0.067 | 0.037 |  |
|  | **243** |  |  |  |  |  |  |  |  |  |  |  |  |  | 0.09 | 0.017 |  |  |  | 0.055 |  |
| **LFMP011** | **N** | 16 | 20 | 29 | 18 | 24 | 23 | 65 | 58 | 31 | 25 | 23 | 129 | 30 | 122 | 29 | 30 | 27 | 29 | 136 | 1 |
|  | **212** |  |  |  |  |  | 0.391 | 0.369 |  |  |  |  | 0.368 | 0.05 | 0.115 | 0.034 | 0.15 | 0.444 | 0.224 | 0.099 |  |
|  | **214** | 1 | 1 | 1 | 1 | 1 | 0.609 |  |  |  |  |  |  |  |  |  |  |  |  |  |  |
|  | **216** |  |  |  |  |  |  | 0.354 | 0.603 | 0.452 | 0.44 | 0.457 | 0.632 | 0.95 | 0.787 | 0.966 | 0.733 | 0.426 | 0.466 | 0.717 | 0.5 |
|  | **218** |  |  |  |  |  |  | 0.277 | 0.397 | 0.548 | 0.56 | 0.543 |  |  | 0.094 |  | 0.083 | 0.037 | 0.31 | 0.132 | 0.5 |
|  | **220** |  |  |  |  |  |  |  |  |  |  |  |  |  | 0.004 |  | 0.033 | 0.093 |  | 0.051 |  |
| **LFMP042** | **N** | 16 | 20 | 30 | 19 | 24 | 25 | 65 | 58 | 30 | 25 | 23 | 129 | 30 | 122 | 30 | 30 | 27 | 30 | 136 | 1 |
|  | **183** | 1 | 1 | 1 | 0.974 | 1 | 1 | 0.446 |  |  |  |  |  |  |  |  |  |  |  |  | 1 |
|  | **185** |  |  |  | 0.026 |  |  |  |  |  |  |  |  |  |  |  |  |  |  |  |  |
|  | **195** |  |  |  |  |  |  |  |  |  |  |  |  |  |  |  |  | 0.13 |  | 0.018 |  |
|  | **197** |  |  |  |  |  |  |  |  | 0.017 | 0.02 |  |  | 0.3 |  |  |  |  |  |  |  |
|  | **199** |  |  |  |  |  |  |  |  |  |  |  |  |  | 0.016 | 0.017 |  | 0.037 |  |  |  |
|  | **201** |  |  |  |  |  |  |  |  |  |  |  |  |  |  |  |  | 0.019 |  | 0.015 |  |
|  | **203** |  |  |  |  |  |  | 0.077 | 0.06 | 0.333 | 0.36 |  |  |  | 0.045 | 0.017 |  | 0.037 |  | 0.026 |  |
|  | **205** |  |  |  |  |  |  | 0.062 | 0.129 |  |  |  |  | 0.633 | 0.152 | 0.283 | 0.233 |  | 0.267 | 0.063 |  |
|  | **207** |  |  |  |  |  |  |  |  | 0.083 | 0.2 |  |  |  | 0.066 | 0.25 |  | 0.167 | 0.233 | 0.169 |  |
|  | **209** |  |  |  |  |  |  | 0.131 | 0.353 | 0.033 |  | 0.717 | 0.547 |  | 0.082 | 0.117 | 0.1 |  | 0.067 | 0.055 |  |
|  | **211** |  |  |  |  |  |  |  |  |  |  |  | 0.008 |  | 0.041 |  |  | 0.019 |  |  |  |
|  | **213** |  |  |  |  |  |  | 0.069 | 0.052 |  |  |  |  |  | 0.008 |  | 0.033 |  |  |  |  |
|  | **215** |  |  |  |  |  |  | 0.062 | 0.043 |  |  |  | 0.24 | 0.067 | 0.074 |  | 0.05 |  | 0.033 | 0.018 |  |
|  | **217** |  |  |  |  |  |  |  |  |  |  |  | 0.008 |  | 0.045 |  |  |  |  | 0.018 |  |
|  | **219** |  |  |  |  |  |  |  |  |  |  |  | 0.054 |  |  |  |  | 0.148 |  | 0.066 |  |
|  | **221** |  |  |  |  |  |  | 0.131 | 0.19 | 0.183 | 0.16 | 0.283 | 0.143 |  | 0.172 | 0.2 | 0.35 | 0.426 | 0.017 | 0.283 |  |
|  | **223** |  |  |  |  |  |  | 0.023 | 0.155 | 0.033 | 0.06 |  |  |  | 0.139 |  | 0.1 |  | 0.383 | 0.173 |  |
|  | **225** |  |  |  |  |  |  |  | 0.017 | 0.217 | 0.16 |  |  |  | 0.094 | 0.05 |  |  |  | 0.059 |  |
|  | **227** |  |  |  |  |  |  |  |  | 0.083 |  |  |  |  | 0.066 | 0.067 | 0.1 | 0.019 |  | 0.037 |  |
|  | **229** |  |  |  |  |  |  |  |  | 0.017 |  |  |  |  |  |  |  |  |  |  |  |
|  | **231** |  |  |  |  |  |  |  |  |  | 0.04 |  |  |  |  |  | 0.033 |  |  |  |  |
| **LFMP052** | **N** | 16 | 20 | 29 | 19 | 24 | 25 | 65 | 58 | 31 | 24 | 23 | 129 | 30 | 122 | 30 | 30 | 27 | 30 | 136 | 1 |
|  | **134** | 0.969 | 0.15 | 0.086 | 0.053 | 0.646 | 0.04 |  |  |  |  |  |  |  |  |  |  |  |  |  |  |
|  | **136** |  | 0.85 |  | 0.316 | 0.021 | 0.1 | 0.292 |  |  |  |  |  |  |  |  |  |  |  |  |  |
|  | **138** | 0.031 |  |  |  |  |  |  |  |  |  |  |  |  |  |  |  |  |  |  |  |
|  | **144** |  |  |  |  |  |  |  |  |  |  |  |  |  |  |  |  |  |  |  | 1 |
|  | **148** |  |  |  |  |  | 0.04 | 0.046 |  |  |  |  |  |  |  |  |  |  |  |  |  |
|  | **152** |  |  | 0.069 | 0.053 | 0.083 | 0.02 |  |  |  |  |  |  |  |  |  |  |  |  |  |  |
|  | **154** |  |  |  | 0.026 | 0.125 | 0.02 |  |  |  |  |  |  |  |  |  |  |  |  |  |  |
|  | **156** |  |  | 0.845 | 0.5 | 0.104 | 0.66 |  |  |  |  |  |  |  |  |  |  |  |  |  |  |
|  | **158** |  |  |  |  |  | 0.04 |  |  |  |  |  |  |  |  |  |  |  |  |  |  |
|  | **160** |  |  |  | 0.053 | 0.021 | 0.06 |  |  |  |  |  |  |  |  |  |  |  |  |  |  |
|  | **162** |  |  |  |  |  | 0.02 |  |  |  |  |  |  |  |  |  |  |  |  |  |  |
|  | **166** |  |  |  |  |  |  |  |  |  |  |  |  |  |  |  |  |  |  | 0.033 |  |
|  | **180** |  |  |  |  |  |  |  |  |  |  |  |  |  | 0.045 | 0.05 |  |  |  |  |  |
|  | **184** |  |  |  |  |  |  |  |  | 0.048 | 0.042 |  |  |  |  |  | 0.2 |  |  | 0.015 |  |
|  | **188** |  |  |  |  |  |  |  |  |  |  |  |  |  |  |  | 0.033 |  |  |  |  |
|  | **190** |  |  |  |  |  |  | 0.115 | 0.078 | 0.048 | 0.042 |  |  |  |  |  | 0.05 |  |  |  |  |
|  | **192** |  |  |  |  |  |  |  | 0.009 | 0.065 | 0.146 |  |  | 0.133 | 0.029 |  | 0.083 | 0.13 |  | 0.059 |  |
|  | **194** |  |  |  |  |  |  |  |  |  |  |  |  |  | 0.131 | 0.183 |  |  |  | 0.011 |  |
|  | **196** |  |  |  |  |  |  | 0.138 | 0.198 | 0.145 | 0.125 |  |  | 0.3 |  |  |  |  | 0.117 | 0.077 |  |
|  | **198** |  |  |  |  |  |  | 0.185 | 0.397 | 0.113 | 0.125 | 1 | 1 | 0.567 | 0.463 | 0.433 | 0.05 | 0.593 | 0.117 | 0.217 |  |
|  | **200** |  |  |  |  |  |  |  |  |  |  |  |  |  | 0.02 |  | 0.017 |  |  |  |  |
|  | **202** |  |  |  |  |  |  |  |  |  |  |  |  |  | 0.008 | 0.017 |  |  |  | 0.07 |  |
|  | **204** |  |  |  |  |  |  | 0.069 | 0.207 | 0.161 | 0.188 |  |  |  | 0.164 | 0.183 | 0.083 | 0.037 | 0.167 | 0.202 |  |
|  | **206** |  |  |  |  |  |  |  |  | 0.145 | 0.104 |  |  |  | 0.061 | 0.133 | 0.367 | 0.185 | 0.183 | 0.099 |  |
|  | **208** |  |  |  |  |  |  |  |  | 0.194 | 0.083 |  |  |  |  |  | 0.017 |  |  |  |  |
|  | **210** |  |  |  |  |  |  | 0.008 | 0.009 |  |  |  |  |  |  |  |  |  | 0.117 | 0.037 |  |
|  | **212** |  |  |  |  |  |  |  |  | 0.016 | 0.042 |  |  |  | 0.02 |  | 0.017 |  | 0.1 | 0.029 |  |
|  | **214** |  |  |  |  |  |  |  |  |  | 0.021 |  |  |  |  |  |  |  |  | 0.015 |  |
|  | **216** |  |  |  |  |  |  |  |  |  |  |  |  |  | 0.004 |  | 0.033 |  |  | 0.066 |  |
|  | **218** |  |  |  |  |  |  |  |  | 0.065 |  |  |  |  | 0.008 |  | 0.017 | 0.056 |  | 0.011 |  |
|  | **220** |  |  |  |  |  |  |  |  |  |  |  |  |  | 0.016 |  | 0.017 |  | 0.167 | 0.004 |  |
|  | **222** |  |  |  |  |  |  | 0.108 |  |  | 0.021 |  |  |  |  |  |  |  |  | 0.026 |  |
|  | **224** |  |  |  |  |  |  | 0.023 | 0.103 |  |  |  |  |  |  |  |  |  |  |  |  |
|  | **226** |  |  |  |  |  |  | 0.008 |  |  | 0.063 |  |  |  |  |  |  |  | 0.017 |  |  |
|  | **228** |  |  |  |  |  |  | 0.008 |  |  |  |  |  |  |  |  |  |  |  |  |  |
|  | **232** |  |  |  |  |  |  |  |  |  |  |  |  |  |  |  | 0.017 |  | 0.017 |  |  |
|  | **234** |  |  |  |  |  |  |  |  |  |  |  |  |  | 0.029 |  |  |  |  |  |  |
|  | **240** |  |  |  |  |  |  |  |  |  |  |  |  |  |  |  |  |  |  | 0.029 |  |
| **LFMP054** | **N** | 16 | 20 | 30 | 19 | 24 | 25 | 65 | 58 | 31 | 25 | 23 | 129 | 30 | 122 | 29 | 30 | 26 | 30 | 136 | 1 |
|  | **125** |  |  |  |  |  |  |  |  |  |  |  |  |  | 0.02 | 0.241 | 0.017 |  |  | 0.007 |  |
|  | **127** |  |  |  |  |  |  |  |  |  |  |  |  |  |  |  |  |  |  | 0.037 |  |
|  | **131** | 1 | 1 | 1 | 1 | 1 | 0.94 | 0.369 |  |  |  |  |  |  |  |  |  |  |  |  |  |
|  | **133** |  |  |  |  |  | 0.06 | 0.054 | 0.095 |  |  |  |  |  |  |  | 0.017 | 0.096 |  | 0.037 | 1 |
|  | **139** |  |  |  |  |  |  |  |  |  |  |  |  |  | 0.025 | 0.086 |  |  |  |  |  |
|  | **141** |  |  |  |  |  |  | 0.5 | 0.828 | 0.742 | 0.76 | 1 | 0.174 | 1 | 0.934 | 0.672 | 0.95 | 0.904 | 1 | 0.901 |  |
|  | **143** |  |  |  |  |  |  | 0.054 | 0.078 |  |  |  | 0.826 |  | 0.008 |  |  |  |  |  |  |
|  | **145** |  |  |  |  |  |  | 0.023 |  | 0.258 | 0.24 |  |  |  |  |  | 0.017 |  |  | 0.011 |  |
|  | **147** |  |  |  |  |  |  |  |  |  |  |  |  |  |  |  |  |  |  | 0.007 |  |
|  | **149** |  |  |  |  |  |  |  |  |  |  |  |  |  | 0.012 |  |  |  |  |  |  |
| **LFMP072** | **N** | 16 | 20 | 29 | 19 | 24 | 25 | 65 | 58 | 31 | 25 | 23 | 129 | 30 | 122 | 30 | 30 | 27 | 30 | 136 | 1 |
|  | **239** |  |  |  |  |  |  |  |  |  |  |  |  |  | 0.082 | 0.133 |  |  |  |  |  |
|  | **241** |  |  |  |  |  |  | 0.138 | 0.129 | 0.323 | 0.22 |  | 0.698 |  | 0.172 | 0.083 | 0.017 | 0.019 | 0.133 | 0.11 |  |
|  | **245** |  |  |  |  |  |  | 0.231 | 0.431 | 0.371 | 0.44 | 0.913 | 0.302 | 1 | 0.348 | 0.333 | 0.617 | 0.667 | 0.75 | 0.478 |  |
|  | **247** |  |  |  |  |  |  | 0.185 | 0.319 | 0.21 | 0.3 | 0.087 |  |  | 0.299 | 0.35 | 0.05 | 0.296 | 0.017 | 0.331 |  |
|  | **249** |  |  |  |  |  |  | 0.038 | 0.121 | 0.097 | 0.04 |  |  |  | 0.098 | 0.1 | 0.217 |  | 0.1 | 0.081 | 1 |
|  | **253** |  |  |  |  | 0.063 | 0.02 |  |  |  |  |  |  |  |  |  |  |  |  |  |  |
|  | **255** |  |  |  |  |  | 0.02 | 0.015 |  |  |  |  |  |  |  |  | 0.1 | 0.019 |  |  |  |
|  | **261** |  |  |  | 0.184 |  |  |  |  |  |  |  |  |  |  |  |  |  |  |  |  |
|  | **263** |  |  | 0.086 | 0.026 | 0.021 |  |  |  |  |  |  |  |  |  |  |  |  |  |  |  |
|  | **265** | 0.094 |  |  |  |  | 0.16 |  |  |  |  |  |  |  |  |  |  |  |  |  |  |
|  | **267** | 0.719 | 1 |  | 0.053 | 0.042 | 0.04 |  |  |  |  |  |  |  |  |  |  |  |  |  |  |
|  | **269** | 0.188 |  | 0.362 | 0.526 | 0.542 | 0.6 | 0.377 |  |  |  |  |  |  |  |  |  |  |  |  |  |
|  | **271** |  |  | 0.552 | 0.158 | 0.083 |  | 0.008 |  |  |  |  |  |  |  |  |  |  |  |  |  |
|  | **273** |  |  |  |  | 0.083 | 0.1 |  |  |  |  |  |  |  |  |  |  |  |  |  |  |
|  | **275** |  |  |  | 0.053 | 0.021 |  |  |  |  |  |  |  |  |  |  |  |  |  |  |  |
|  | **277** |  |  |  |  | 0.042 |  |  |  |  |  |  |  |  |  |  |  |  |  |  |  |
|  | **279** |  |  |  |  | 0.083 | 0.04 |  |  |  |  |  |  |  |  |  |  |  |  |  |  |
|  | **283** |  |  |  |  | 0.021 | 0.02 | 0.008 |  |  |  |  |  |  |  |  |  |  |  |  |  |
| **LFMP100** | **N** | 16 | 20 | 29 | 19 | 24 | 25 | 65 | 58 | 31 | 25 | 23 | 129 | 30 | 122 | 30 | 30 | 27 | 30 | 136 | 1 |
|  | **185** |  |  |  |  |  |  | 0.315 | 0.552 | 0.677 | 0.64 | 0.413 | 0.132 |  | 0.234 | 0.383 | 0.167 | 0.093 | 0.05 | 0.268 |  |
|  | **187** |  |  |  |  | 0.083 |  |  |  |  |  |  |  |  |  |  |  |  |  |  | 1 |
|  | **189** |  |  | 0.759 | 0.184 | 0.021 | 0.02 | 0.015 |  |  |  |  |  |  |  |  |  |  |  |  |  |
|  | **193** | 1 | 1 | 0.241 | 0.395 | 0.875 | 0.98 | 0.508 | 0.164 |  |  |  | 0.403 |  | 0.156 | 0.3 | 0.083 | 0.074 |  | 0.051 |  |
|  | **195** |  |  |  | 0.421 | 0.021 |  | 0.162 | 0.284 | 0.323 | 0.34 | 0.587 | 0.465 | 1 | 0.607 | 0.317 | 0.733 | 0.778 | 0.95 | 0.658 |  |
|  | **197** |  |  |  |  |  |  |  |  |  |  |  |  |  |  |  |  | 0.037 |  |  |  |
|  | **203** |  |  |  |  |  |  |  |  |  | 0.02 |  |  |  | 0.004 |  | 0.017 | 0.019 |  | 0.022 |  |
| **LFMP106** | **N** | 15 | 14 | 30 | 17 | 23 | 23 | 65 | 58 | 31 | 25 | 23 | 129 | 30 | 122 | 30 | 30 | 27 | 30 | 136 | 1 |
|  | **143** |  |  |  |  |  |  | 0.008 |  |  |  |  |  |  |  |  |  |  |  |  |  |
|  | **149** |  |  |  |  |  |  | 0.008 |  |  |  |  |  |  |  |  |  | 0.074 | 0.117 | 0.026 |  |
|  | **153** |  |  |  |  |  |  |  |  |  |  |  |  |  | 0.016 | 0.017 |  |  |  | 0.029 |  |
|  | **155** |  |  |  |  |  |  |  |  |  |  |  |  |  | 0.016 |  |  |  |  | 0.007 |  |
|  | **157** |  |  |  |  |  |  | 0.077 | 0.103 | 0.21 | 0.08 | 0.565 | 0.686 | 0.467 | 0.463 | 0.383 | 0.383 | 0.222 | 0.2 | 0.268 |  |
|  | **159** |  |  |  |  |  |  | 0.062 | 0.009 |  |  |  |  |  | 0.004 |  |  |  |  |  |  |
|  | **161** |  |  |  |  |  |  | 0.108 | 0.147 |  |  |  |  |  | 0.033 | 0.017 |  |  | 0.017 | 0.015 |  |
|  | **163** |  |  |  |  |  |  |  |  | 0.129 | 0.12 | 0.435 |  |  | 0.107 | 0.183 | 0.15 | 0.111 |  | 0.254 |  |
|  | **165** |  |  |  |  |  |  | 0.231 | 0.431 | 0.177 | 0.36 |  | 0.314 |  | 0.221 | 0.25 | 0.217 | 0.389 | 0.483 | 0.217 |  |
|  | **167** | 0.033 |  | 0.35 |  |  | 0.043 | 0.169 | 0.31 | 0.016 |  |  |  | 0.533 | 0.082 | 0.117 | 0.05 | 0.019 | 0.183 | 0.154 |  |
|  | **169** |  |  | 0.067 | 0.118 | 0.043 | 0.022 |  |  | 0.323 | 0.32 |  |  |  | 0.033 | 0.017 | 0.2 | 0.185 |  | 0.029 |  |
|  | **171** | 0.167 |  |  |  |  |  |  |  | 0.145 | 0.12 |  |  |  | 0.025 | 0.017 |  |  |  |  |  |
|  | **173** | 0.033 |  |  |  |  | 0.022 | 0.338 |  |  |  |  |  |  |  |  |  |  |  |  |  |
|  | **175** | 0.1 |  |  |  |  |  |  |  |  |  |  |  |  |  |  |  |  |  |  |  |
|  | **177** |  |  | 0.1 |  |  | 0.174 |  |  |  |  |  |  |  |  |  |  |  |  |  |  |
|  | **179** |  |  |  |  |  | 0.13 |  |  |  |  |  |  |  |  |  |  |  |  |  |  |
|  | **181** | 0.033 | 0.143 |  | 0.029 | 0.217 | 0.196 |  |  |  |  |  |  |  |  |  |  |  |  |  |  |
|  | **183** | 0.467 | 0.607 | 0.183 | 0.059 | 0.543 | 0.022 |  |  |  |  |  |  |  |  |  |  |  |  |  |  |
|  | **185** | 0.067 | 0.25 |  | 0.382 |  | 0.174 |  |  |  |  |  |  |  |  |  |  |  |  |  | 0.5 |
|  | **187** | 0.1 |  | 0.3 | 0.294 | 0.13 | 0.022 |  |  |  |  |  |  |  |  |  |  |  |  |  | 0.5 |
|  | **189** |  |  |  | 0.029 |  |  |  |  |  |  |  |  |  |  |  |  |  |  |  |  |
|  | **191** |  |  |  |  | 0.065 | 0.152 |  |  |  |  |  |  |  |  |  |  |  |  |  |  |
|  | **193** |  |  |  |  |  | 0.043 |  |  |  |  |  |  |  |  |  |  |  |  |  |  |
|  | **195** |  |  |  | 0.088 |  |  |  |  |  |  |  |  |  |  |  |  |  |  |  |  |
| **Mpe1.A07** | **N** | 16 | 20 | 30 | 19 | 24 | 25 | 65 | 58 | 31 | 25 | 23 | 128 | 30 | 122 | 30 | 30 | 27 | 30 | 136 | 1 |
|  | **257** |  |  |  |  | 0.021 |  |  |  |  |  |  |  |  |  |  |  |  |  |  |  |
|  | **261** | 0.125 | 1 | 0.1 | 0.079 | 0.083 | 0.16 | 0.662 | 0.509 | 0.758 | 0.6 | 1 | 0.223 | 0.067 | 0.283 | 0.35 | 0.233 | 0.315 | 0.417 | 0.489 |  |
|  | **263** | 0.875 |  | 0.9 | 0.921 | 0.896 | 0.84 | 0.338 | 0.491 | 0.242 | 0.4 |  | 0.777 | 0.833 | 0.668 | 0.617 | 0.767 | 0.685 | 0.583 | 0.511 | 1 |
|  | **265** |  |  |  |  |  |  |  |  |  |  |  |  | 0.1 | 0.049 | 0.033 |  |  |  |  |  |
| **Mpe1.B10** | **N** | 16 | 20 | 30 | 19 | 24 | 25 | 65 | 58 | 31 | 25 | 23 | 129 | 30 | 122 | 30 | 30 | 27 | 30 | 136 | 1 |
|  | **169** |  |  |  |  |  |  |  |  |  | 0.02 |  |  |  |  |  |  |  |  |  |  |
|  | **173** |  |  |  |  |  |  |  |  |  |  |  | 0.376 |  | 0.012 | 0.1 | 0.167 | 0.056 |  |  |  |
|  | **177** |  |  | 0.017 |  |  |  |  |  |  |  |  |  |  |  |  |  |  |  |  |  |
|  | **179** |  |  |  |  |  |  | 0.469 | 0.776 | 0.871 | 0.9 | 0.957 | 0.624 | 0.95 | 0.84 | 0.867 | 0.767 | 0.944 | 0.917 | 0.949 |  |
|  | **185** | 1 | 1 | 0.983 | 1 | 1 | 1 | 0.531 | 0.224 | 0.129 | 0.08 | 0.043 |  | 0.05 | 0.148 | 0.033 | 0.067 |  | 0.083 | 0.051 | 1 |
| **Mpe1.F01** | **N** | 16 | 20 | 30 | 19 | 24 | 25 | 65 | 58 | 31 | 25 | 23 | 129 | 30 | 122 | 30 | 30 | 27 | 30 | 136 | 1 |
|  | **287** |  |  |  |  |  |  | 0.277 | 0.466 | 0.597 | 0.84 | 1 |  | 0.5 | 0.725 | 0.75 | 0.367 | 0.907 | 0.867 | 0.721 |  |
|  | **289** |  |  |  |  |  |  | 0.277 | 0.534 | 0.403 | 0.16 |  | 1 | 0.5 | 0.275 | 0.25 | 0.467 | 0.093 | 0.133 | 0.279 |  |
|  | **291** |  |  |  |  |  |  |  |  |  |  |  |  |  |  |  | 0.167 |  |  |  |  |
|  | **293** |  |  |  |  |  |  |  |  |  |  |  |  |  |  |  |  |  |  |  | 1 |
|  | **309** |  |  |  |  |  |  | 0.415 |  |  |  |  |  |  |  |  |  |  |  |  |  |
|  | **311** |  |  |  |  | 0.021 |  |  |  |  |  |  |  |  |  |  |  |  |  |  |  |
|  | **313** |  |  |  | 0.158 | 0.042 | 0.22 |  |  |  |  |  |  |  |  |  |  |  |  |  |  |
|  | **315** |  |  |  | 0.053 |  |  |  |  |  |  |  |  |  |  |  |  |  |  |  |  |
|  | **317** |  |  |  | 0.105 |  | 0.02 |  |  |  |  |  |  |  |  |  |  |  |  |  |  |
|  | **321** | 0.781 | 0.275 | 1 | 0.395 | 0.104 | 0.06 | 0.015 |  |  |  |  |  |  |  |  |  |  |  |  |  |
|  | **323** | 0.219 | 0.725 |  |  | 0.042 |  |  |  |  |  |  |  |  |  |  |  |  |  |  |  |
|  | **325** |  |  |  | 0.026 | 0.208 | 0.34 |  |  |  |  |  |  |  |  |  |  |  |  |  |  |
|  | **327** |  |  |  | 0.026 | 0.271 | 0.1 |  |  |  |  |  |  |  |  |  |  |  |  |  |  |
|  | **329** |  |  |  | 0.237 | 0.146 | 0.16 |  |  |  |  |  |  |  |  |  |  |  |  |  |  |
|  | **331** |  |  |  |  | 0.125 | 0.08 | 0.015 |  |  |  |  |  |  |  |  |  |  |  |  |  |
|  | **333** |  |  |  |  | 0.042 |  |  |  |  |  |  |  |  |  |  |  |  |  |  |  |
|  | **335** |  |  |  |  |  | 0.02 |  |  |  |  |  |  |  |  |  |  |  |  |  |  |
| **Mpe1.H04** | **N** | 16 | 20 | 30 | 19 | 24 | 25 | 64 | 58 | 31 | 25 | 23 | 129 | 30 | 122 | 30 | 30 | 26 | 30 | 136 | 1 |
|  | **269** |  |  |  |  |  |  | 0.148 | 0.388 | 0.032 | 0.08 |  |  |  | 0.082 | 0.133 | 0.35 | 0.308 |  | 0.063 |  |
|  | **271** |  |  |  |  |  |  | 0.477 | 0.612 | 0.968 | 0.92 | 1 | 1 | 1 | 0.893 | 0.833 | 0.65 | 0.692 | 1 | 0.864 |  |
|  | **273** |  |  |  |  |  |  |  |  |  |  |  |  |  | 0.025 | 0.033 |  |  |  | 0.074 |  |
|  | **275** |  |  |  |  |  |  |  |  |  |  |  |  |  |  |  |  |  |  |  | 1 |
|  | **281** |  | 1 |  |  |  |  |  |  |  |  |  |  |  |  |  |  |  |  |  |  |
|  | **283** |  |  |  | 0.079 | 0.063 | 0.08 |  |  |  |  |  |  |  |  |  |  |  |  |  |  |
|  | **287** |  |  | 0.283 | 0.342 | 0.75 | 0.86 | 0.055 |  |  |  |  |  |  |  |  |  |  |  |  |  |
|  | **289** | 0.969 |  | 0.717 | 0.579 | 0.188 | 0.06 | 0.297 |  |  |  |  |  |  |  |  |  |  |  |  |  |
|  | **291** | 0.031 |  |  |  |  |  | 0.023 |  |  |  |  |  |  |  |  |  |  |  |  |  |
| **Mpe2.B02** | **N** | 16 | 20 | 30 | 19 | 24 | 25 | 65 | 58 | 30 | 25 | 23 | 129 | 30 | 122 | 30 | 30 | 26 | 30 | 136 | 1 |
|  | **154** | 1 |  | 0.017 | 0.395 | 0.146 | 0.16 | 0.692 | 0.638 | 0.833 | 0.78 | 0.935 | 0.043 | 1 | 0.697 | 0.683 | 0.667 | 0.788 | 0.667 | 0.574 | 1 |
|  | **156** |  | 1 | 0.983 | 0.605 | 0.854 | 0.84 | 0.308 | 0.362 | 0.167 | 0.22 | 0.065 | 0.957 |  | 0.303 | 0.317 | 0.333 | 0.192 | 0.333 | 0.426 |  |
|  | **160** |  |  |  |  |  |  |  |  |  |  |  |  |  |  |  |  | 0.019 |  |  |  |
| **Mpe2.D11** | **N** | 16 | 20 | 30 | 19 | 24 | 25 | 65 | 58 | 31 | 25 | 23 | 129 | 30 | 122 | 30 | 30 | 27 | 30 | 136 | 1 |
|  | **159** |  | 0.3 | 1 | 0.763 | 0.479 | 0.7 |  |  |  |  |  |  |  |  |  |  |  |  |  |  |
|  | **165** | 0.938 | 0.7 |  | 0.184 | 0.333 | 0.22 | 0.277 |  |  |  |  |  |  |  |  |  |  |  |  |  |
|  | **167** | 0.063 |  |  | 0.053 | 0.167 | 0.08 |  |  |  |  |  |  |  | 0.004 |  |  |  |  |  |  |
|  | **171** |  |  |  |  |  |  |  |  |  |  |  |  |  | 0.266 | 0.167 | 0.467 | 0.426 |  | 0.114 |  |
|  | **173** |  |  |  |  |  |  |  |  |  |  |  | 0.601 |  |  |  |  |  |  | 0.044 |  |
|  | **175** |  |  |  |  |  |  |  |  |  |  |  |  |  | 0.078 | 0.017 | 0.05 |  | 0.317 | 0.103 |  |
|  | **177** |  |  |  |  | 0.021 |  | 0.108 | 0.241 |  |  |  |  |  | 0.029 |  | 0.033 |  |  |  |  |
|  | **179** |  |  |  |  |  |  | 0.154 | 0.112 | 0.145 | 0.18 |  |  |  | 0.102 | 0.117 | 0.15 | 0.074 | 0.017 | 0.154 |  |
|  | **181** |  |  |  |  |  |  |  |  | 0.258 | 0.14 | 0.391 |  |  | 0.074 | 0.133 | 0.067 | 0.093 |  | 0.092 |  |
|  | **183** |  |  |  |  |  |  | 0.377 | 0.552 | 0.355 | 0.42 | 0.543 | 0.399 | 0.633 | 0.27 | 0.45 | 0.167 | 0.241 | 0.233 | 0.342 |  |
|  | **185** |  |  |  |  |  |  |  | 0.009 | 0.081 | 0.14 |  |  |  | 0.057 | 0.05 | 0.05 | 0.148 | 0.433 | 0.074 |  |
|  | **187** |  |  |  |  |  |  |  | 0.009 | 0.113 | 0.04 | 0.065 |  | 0.283 | 0.07 | 0.017 | 0.017 | 0.019 |  | 0.074 | 1 |
|  | **189** |  |  |  |  |  |  |  |  |  |  |  |  | 0.083 |  |  |  |  |  |  |  |
|  | **191** |  |  |  |  |  |  |  |  |  |  |  |  |  | 0.012 |  |  |  |  |  |  |
|  | **193** |  |  |  |  |  |  |  |  |  |  |  |  |  |  |  |  |  |  | 0.004 |  |
|  | **197** |  |  |  |  |  |  | 0.085 | 0.078 |  |  |  |  |  | 0.037 | 0.05 |  |  |  |  |  |
|  | **201** |  |  |  |  |  |  |  |  | 0.048 | 0.08 |  |  |  |  |  |  |  |  |  |  |
| **Mpe2.F07** | **N** | 16 | 20 | 30 | 19 | 24 | 25 | 64 | 58 | 31 | 25 | 23 | 129 | 30 | 121 | 29 | 30 | 26 | 30 | 136 | 1 |
|  | **158** | 0.063 |  |  |  |  |  |  |  | 0.177 | 0.24 |  |  |  |  |  |  |  |  |  | 1 |
|  | **160** | 0.938 | 1 | 1 | 1 | 1 | 1 | 0.875 | 0.871 | 0.806 | 0.72 | 1 | 1 | 1 | 0.855 | 0.759 | 0.733 | 0.942 | 1 | 0.941 |  |
|  | **162** |  |  |  |  |  |  | 0.125 | 0.129 | 0.016 | 0.04 |  |  |  | 0.145 | 0.241 | 0.267 | 0.058 |  | 0.059 |  |
| **Mpe3.B11** | **N** | 16 | 20 | 30 | 18 | 24 | 25 | 65 | 56 | 31 | 25 | 23 | 129 | 30 | 122 | 30 | 30 | 27 | 30 | 136 | 1 |
|  | **347** |  |  |  |  |  |  |  |  | 0.129 | 0.04 |  |  |  |  |  |  | 0.019 |  | 0.044 |  |
|  | **349** |  |  | 0.017 | 0.472 | 0.042 | 0.58 |  |  |  |  |  |  |  |  |  |  |  |  |  |  |
|  | **351** |  |  |  | 0.028 | 0.021 | 0.12 | 0.062 |  |  |  |  |  |  | 0.008 |  |  | 0.037 | 0.2 | 0.007 |  |
|  | **353** |  |  |  |  |  |  | 0.408 | 0.536 | 0.097 | 0.14 |  |  |  | 0.701 | 0.783 | 0.733 | 0.481 | 0.45 | 0.592 |  |
|  | **355** | 0.406 |  |  |  |  |  | 0.262 | 0.464 | 0.484 | 0.62 | 0.957 | 0.236 | 0.783 | 0.221 | 0.183 | 0.2 | 0.463 | 0.15 | 0.342 |  |
|  | **357** | 0.313 |  |  |  | 0.021 |  |  |  | 0.081 | 0.06 | 0.043 |  |  | 0.061 | 0.033 | 0.067 |  | 0.2 | 0.015 |  |
|  | **359** | 0.25 |  |  | 0.194 | 0.313 | 0.26 | 0.223 |  |  |  |  |  |  |  |  |  |  |  |  |  |
|  | **361** |  |  |  |  |  |  | 0.031 |  |  |  |  |  |  |  |  |  |  |  |  |  |
|  | **363** |  |  | 0.65 | 0.278 | 0.438 | 0.02 |  |  | 0.194 | 0.04 |  |  |  |  |  |  |  |  |  |  |
|  | **365** |  |  | 0.017 |  |  |  | 0.008 |  |  |  |  |  |  |  |  |  |  |  |  |  |
|  | **367** |  | 1 |  |  | 0.042 |  |  |  |  |  |  |  |  |  |  |  |  |  |  |  |
|  | **369** | 0.031 |  | 0.317 | 0.028 |  |  |  |  | 0.016 | 0.1 |  |  |  | 0.008 |  |  |  |  |  |  |
|  | **371** |  |  |  |  | 0.083 |  |  |  |  |  |  |  |  |  |  |  |  |  |  |  |
|  | **373** |  |  |  |  |  |  | 0.008 |  |  |  |  |  |  |  |  |  |  |  |  | 1 |
|  | **375** |  |  |  |  |  | 0.02 |  |  |  |  |  | 0.764 | 0.217 |  |  |  |  |  |  |  |
|  | **377** |  |  |  |  | 0.042 |  |  |  |  |  |  |  |  |  |  |  |  |  |  |  |
| **Mpe3.G04** | **N** | 16 | 18 | 30 | 18 | 24 | 25 | 63 | 54 | 31 | 25 | 23 | 129 | 30 | 122 | 30 | 30 | 27 | 30 | 136 | 1 |
|  | **319** |  |  |  |  |  |  |  |  |  |  |  |  |  |  |  |  |  |  |  | 1 |
|  | **325** |  |  |  |  |  |  |  |  |  |  |  |  |  |  |  |  |  |  | 0.022 |  |
|  | **327** | 0.188 | 0.889 |  | 0.194 | 0.438 | 0.44 | 0.024 |  |  |  |  |  |  | 0.008 |  |  |  | 0.05 | 0.007 |  |
|  | **329** |  |  |  |  |  |  | 0.397 | 0.769 | 0.71 | 0.58 | 1 | 0.058 | 0.95 | 0.566 | 0.7 | 0.733 | 0.185 | 0.233 | 0.522 |  |
|  | **331** |  |  |  |  |  |  | 0.103 | 0.231 | 0.145 | 0.24 |  | 0.942 | 0.05 | 0.381 | 0.233 | 0.2 | 0.426 | 0.217 | 0.357 |  |
|  | **333** | 0.75 |  | 1 | 0.806 | 0.542 | 0.56 | 0.016 |  | 0.113 | 0.16 |  |  |  | 0.012 | 0.033 | 0.033 | 0.148 | 0.167 | 0.044 |  |
|  | **335** | 0.063 | 0.111 |  |  | 0.021 |  | 0.008 |  |  |  |  |  |  |  |  |  | 0.111 | 0.017 | 0.007 |  |
|  | **337** |  |  |  |  |  |  |  |  |  |  |  |  |  |  |  |  | 0.037 |  | 0.011 |  |
|  | **345** |  |  |  |  |  |  |  |  |  |  |  |  |  | 0.004 |  |  |  |  | 0.029 |  |
|  | **347** |  |  |  |  |  |  | 0.016 |  |  |  |  |  |  |  |  |  |  |  |  |  |
|  | **349** |  |  |  |  |  |  | 0.437 |  |  |  |  |  |  |  |  |  |  |  |  |  |
|  | **353** |  |  |  |  |  |  |  |  | 0.032 | 0.02 |  |  |  |  |  |  |  | 0.05 |  |  |
|  | **355** |  |  |  |  |  |  |  |  |  |  |  |  |  |  |  | 0.033 |  |  |  |  |
|  | **357** |  |  |  |  |  |  |  |  |  |  |  |  |  | 0.029 | 0.033 |  | 0.056 | 0.267 |  |  |
|  | **359** |  |  |  |  |  |  |  |  |  |  |  |  |  |  |  |  | 0.037 |  |  |  |
| **Mpe3.G12** | **N** | 16 | 20 | 30 | 19 | 24 | 25 | 65 | 58 | 31 | 25 | 23 | 129 | 30 | 122 | 30 | 30 | 27 | 30 | 136 | 1 |
|  | **159** |  |  |  |  |  |  |  |  |  |  |  |  |  | 0.004 |  | 0.083 |  |  | 0.063 |  |
|  | **161** |  |  |  |  |  |  |  |  |  |  |  |  |  | 0.008 |  |  |  | 0.017 | 0.004 |  |
|  | **165** |  |  |  |  |  |  |  |  |  |  |  |  |  |  |  |  |  |  | 0.007 |  |
|  | **173** |  |  |  |  |  |  |  |  |  |  |  | 0.004 |  | 0.262 | 0.367 | 0.517 | 0.463 | 0.183 | 0.228 |  |
|  | **177** |  |  |  |  |  |  |  | 0.017 | 0.194 | 0.2 |  |  |  | 0.008 |  |  |  | 0.017 |  |  |
|  | **179** |  |  |  |  |  |  | 0.562 | 0.948 | 0.145 | 0.28 | 0.478 | 0.996 | 1 | 0.393 | 0.167 | 0.183 | 0.37 | 0.483 | 0.401 |  |
|  | **181** |  |  |  |  |  |  | 0.015 | 0.034 |  |  | 0.522 |  |  | 0.119 | 0.067 |  |  | 0.033 | 0.055 |  |
|  | **183** |  |  |  |  |  |  |  |  | 0.597 | 0.44 |  |  |  | 0.197 | 0.4 | 0.217 | 0.167 | 0.15 | 0.217 |  |
|  | **185** |  |  |  |  | 0.042 |  |  |  | 0.016 | 0.04 |  |  |  |  |  |  |  | 0.117 |  |  |
|  | **187** |  |  |  |  |  |  |  |  | 0.048 | 0.04 |  |  |  | 0.008 |  |  |  |  | 0.026 |  |
|  | **195** | 0.219 |  |  |  |  | 0.02 |  |  |  |  |  |  |  |  |  |  |  |  |  |  |
|  | **203** | 0.594 |  |  |  |  |  |  |  |  |  |  |  |  |  |  |  |  |  |  |  |
|  | **205** | 0.156 | 0.875 |  |  | 0.021 |  |  |  |  |  |  |  |  |  |  |  |  |  |  |  |
|  | **207** |  |  |  |  | 0.042 |  |  |  |  |  |  |  |  |  |  |  |  |  |  |  |
|  | **209** |  | 0.125 |  |  | 0.083 | 0.06 | 0.015 |  |  |  |  |  |  |  |  |  |  |  |  |  |
|  | **213** |  |  |  | 0.158 | 0.125 | 0.12 |  |  |  |  |  |  |  |  |  |  |  |  |  |  |
|  | **215** |  |  | 0.017 | 0.053 | 0.271 | 0.06 | 0.331 |  |  |  |  |  |  |  |  |  |  |  |  |  |
|  | **217** | 0.031 |  | 0.05 | 0.105 | 0.083 | 0.5 |  |  |  |  |  |  |  |  |  |  |  |  |  | 1 |
|  | **219** |  |  |  | 0.132 | 0.042 | 0.06 | 0.054 |  |  |  |  |  |  |  |  |  |  |  |  |  |
|  | **223** |  |  | 0.05 |  | 0.021 | 0.02 | 0.023 |  |  |  |  |  |  |  |  |  |  |  |  |  |
|  | **225** |  |  |  | 0.263 | 0.021 |  |  |  |  |  |  |  |  |  |  |  |  |  |  |  |
|  | **227** |  |  | 0.5 | 0.184 |  |  |  |  |  |  |  |  |  |  |  |  |  |  |  |  |
|  | **229** |  |  | 0.233 |  | 0.063 | 0.14 |  |  |  |  |  |  |  |  |  |  |  |  |  |  |
|  | **231** |  |  | 0.1 |  | 0.021 | 0.02 |  |  |  |  |  |  |  |  |  |  |  |  |  |  |
|  | **233** |  |  | 0.05 |  |  |  |  |  |  |  |  |  |  |  |  |  |  |  |  |  |
|  | **235** |  |  |  | 0.026 | 0.042 |  |  |  |  |  |  |  |  |  |  |  |  |  |  |  |
|  | **237** |  |  |  |  | 0.021 |  |  |  |  |  |  |  |  |  |  |  |  |  |  |  |
|  | **239** |  |  |  | 0.079 | 0.104 |  |  |  |  |  |  |  |  |  |  |  |  |  |  |  |

Appendix 10. Pairwise population values of microsatellite *F*_ST_ (Table S10A), microsatellite *R*_ST_ (Table S10B) and mtDNA Φ_ST_ (Table S10C) and detailed results of SPAGeDi tests.

High population differentiation, historical within the HNB and contemporary in the MBD, was evidenced by high pairwise population Φ_ST_– (mtDNA; Table S10C) and *F*_ST_– (microsatellites; Table S10A) values. Mitochondrial Φ_ST_–values ranged from 0.58 to 1 in the HNB, from 0.03 to 1 in the MDB and from 0.64 to 1 between HNB and MDB populations (including translocated from MDB Cataract Dam and Yarra but excluding admixed Cataract River). Of 120 *F*-statistics tests, 101 were significant at P<0.001 and 19 were not; of these 16 involved populations from southern MDB rivers (tributaries of Murray River) and/or two translocated populations (Cataract Dam and Yarra), supporting historical gene flow; one (Cataract River and Little River) still showed high differentiation (Φ_ST_ =0.21 and P= 0.004), and two others (Abercrombie-Wheeny, Φ_ST_ = 0.97, P= 0.002 and Lachlan-Abercrombie, Φ_ST_ =0.04, P>0.05) involved small sample sizes (<8).

Microsatellite *F*_ST_–values ranged from 0.14 to 0.75 in the HNB, from 0.01 to 0.57 in the MDB and from 0.44 to 0.82 between HNB and MDB populations (Table S10A). All but one of the 171 *F*_ST_- values were significant at P<0.001, indicating strong contemporary population structure; the exception was Lachlan-Abercrombie comparison (*F*_ST_=0.01, P>0.05) which supported previously inferred contemporary connectivity (Faulks et al., 2011).

When difference in microsatellite allele sizes was considered in addition to allele frequencies, then levels of population differentiation (as measured by *R*_ST_–values, compared to those measured by *F*_ST_–values) remained similar for MDB (range 0 to 0.54), somewhat increased for HNB (range 0.1 to 0.83) and significantly increased between HNB and MDB populations (range 0.92 to 0.99) (Table S10A). Analysis in SPAGeDi (Table S10B) confirmed that evolution of allele sizes has contributed to divergence between coastal and MDB populations, and also to divergence among some HNB populations. Observed *R*_ST_–values were significantly (P<0.05) higher than permuted *pR*_ST_–values for all HNB vs MDB comparisons (except some involving admixed Cataract River population) and for seven within-HNB comparisons (Wollemi vs Wheeny, Glenbrook, Erskine and Little; and Wheeny vs Glenbrook, Erskine and Little). Although overall *R*_ST_/*pR*_ST_ tests across loci were not significant for within-MDB pairs of populations, some tests for individual loci were significant. For example, two of the more-variable loci, Mpe2.D11 and LFMP106, showed significant allele-size differentiation for nine and five (respectively) comparisons, mainly involving Abercrombie or Lachlan vs southern MDB populations (data not shown), indicating that at some loci allele size differences have evolved between northern and southern MDB rivers during population divergence.

Table S10A. Pairwise population values of microsatellite *F*_ST._ All values significant at P<0.001 are in plain font, not significant in italic. Cells are shaded by their relative values; Names of populations with HNB lineages are shaded blue, MDB lineages- orange; Cataract River (not shaded) has both HNB and MDB lineages.

| *F*_ST_ msats | Wollemi Creek | Wheeny Creek | Glenbrook Creek | Erskine Creek | Kowmung River | Little River | Cataract River | Cataract Dam | Abercrombie River | Lachlan River | Adjungbilly Creek | Cotter River | Murrumbidgee River | Dartmouth Lake | Hollands Creek | Sevens Creek | Hughes Creek | King Parrot Creek |
| --- | --- | --- | --- | --- | --- | --- | --- | --- | --- | --- | --- | --- | --- | --- | --- | --- | --- | --- |
| Wollemi Creek |  |  |  |  |  |  |  |  |  |  |  |  |  |  |  |  |  |  |
| Wheeny Creek | 0.69 |  |  |  |  |  |  |  |  |  |  |  |  |  |  |  |  |  |
| Glenbrook Creek | 0.60 | 0.75 |  |  |  |  |  |  |  |  |  |  |  |  |  |  |  |  |
| Erskine Creek | 0.35 | 0.57 | 0.23 |  |  |  |  |  |  |  |  |  |  |  |  |  |  |  |
| Kowmung River | 0.34 | 0.50 | 0.36 | 0.15 |  |  |  |  |  |  |  |  |  |  |  |  |  |  |
| Little River | 0.43 | 0.56 | 0.39 | 0.14 | 0.15 |  |  |  |  |  |  |  |  |  |  |  |  |  |
| Cataract River | 0.39 | 0.43 | 0.46 | 0.32 | 0.32 | 0.35 |  |  |  |  |  |  |  |  |  |  |  |  |
| Cataract Dam | 0.55 | 0.58 | 0.59 | 0.48 | 0.48 | 0.50 | 0.13 |  |  |  |  |  |  |  |  |  |  |  |
| Abercrombie River | 0.55 | 0.61 | 0.61 | 0.47 | 0.49 | 0.51 | 0.19 | 0.16 |  |  |  |  |  |  |  |  |  |  |
| Lachlan River | 0.56 | 0.63 | 0.62 | 0.47 | 0.49 | 0.51 | 0.18 | 0.14 | *0.01* |  |  |  |  |  |  |  |  |  |
| Adjungbilly Creek | 0.74 | 0.80 | 0.77 | 0.65 | 0.65 | 0.67 | 0.29 | 0.24 | 0.23 | 0.23 |  |  |  |  |  |  |  |  |
| Cotter River | 0.71 | 0.72 | 0.71 | 0.64 | 0.63 | 0.65 | 0.40 | 0.39 | 0.47 | 0.47 | 0.57 |  |  |  |  |  |  |  |
| Murrumbidgee River | 0.76 | 0.82 | 0.78 | 0.66 | 0.67 | 0.69 | 0.32 | 0.26 | 0.33 | 0.32 | 0.43 | 0.51 |  |  |  |  |  |  |
| Dartmouth Lake | 0.52 | 0.55 | 0.55 | 0.45 | 0.46 | 0.48 | 0.17 | 0.11 | 0.15 | 0.12 | 0.24 | 0.34 | 0.20 |  |  |  |  |  |
| Hollands Creek | 0.56 | 0.62 | 0.61 | 0.47 | 0.48 | 0.50 | 0.17 | 0.13 | 0.14 | 0.12 | 0.29 | 0.43 | 0.29 | 0.03 |  |  |  |  |
| Sevens Creek | 0.55 | 0.62 | 0.60 | 0.46 | 0.47 | 0.49 | 0.19 | 0.14 | 0.17 | 0.16 | 0.32 | 0.45 | 0.28 | 0.06 | 0.07 |  |  |  |
| Hughes Creek | 0.58 | 0.66 | 0.64 | 0.49 | 0.50 | 0.52 | 0.20 | 0.18 | 0.18 | 0.13 | 0.29 | 0.44 | 0.29 | 0.07 | 0.11 | 0.10 |  |  |
| King Parrot Creek | 0.60 | 0.65 | 0.64 | 0.50 | 0.51 | 0.54 | 0.20 | 0.17 | 0.19 | 0.15 | 0.32 | 0.46 | 0.28 | 0.09 | 0.15 | 0.13 | 0.11 |  |
| Yarra River | 0.51 | 0.53 | 0.53 | 0.44 | 0.45 | 0.47 | 0.16 | 0.09 | 0.10 | 0.08 | 0.19 | 0.35 | 0.20 | 0.03 | 0.05 | 0.06 | 0.07 | 0.07 |

Table S10B. Pairwise population values of microsatellite *R*_ST._ All values significant at P<0.001 are in plain font, not significant in italic. Cells are shaded by their relative values; *R*_ST_-values that are significantly (P<0.05) larger than permuted p*R*_ST_- values (e.g. larger than would be expected if mutations did not contribute to divergence; SPAGeDi) are shown in red font. Names of populations with HNB lineages are shaded blue, MDB lineages- orange; Cataract River (not shaded) has both HNB and MDB lineages.

| *R*_ST_ msats | Wollemi Creek | Wheeny Creek | Glenbrook Creek | Erskine Creek | Kowmung River | Little River | Cataract River | Cataract Dam | Abercrombie River | Lachlan River | Adjungbilly Creek | Cotter River | Murrumbidgee River | Dartmouth Lake | Hollands Creek | Sevens Creek | Hughes Creek | King Parrot Creek |
| --- | --- | --- | --- | --- | --- | --- | --- | --- | --- | --- | --- | --- | --- | --- | --- | --- | --- | --- |
| Wollemi Creek |  |  |  |  |  |  |  |  |  |  |  |  |  |  |  |  |  |  |
| Wheeny Creek | 0.72 |  |  |  |  |  |  |  |  |  |  |  |  |  |  |  |  |  |
| Glenbrook Creek | 0.83 | 0.83 |  |  |  |  |  |  |  |  |  |  |  |  |  |  |  |  |
| Erskine Creek | 0.63 | 0.68 | 0.31 |  |  |  |  |  |  |  |  |  |  |  |  |  |  |  |
| Kowmung River | 0.42 | 0.39 | 0.39 | 0.17 |  |  |  |  |  |  |  |  |  |  |  |  |  |  |
| Little River | 0.64 | 0.70 | 0.42 | 0.10 | 0.27 |  |  |  |  |  |  |  |  |  |  |  |  |  |
| Cataract River | 0.51 | 0.53 | 0.54 | 0.50 | 0.55 | 0.48 |  |  |  |  |  |  |  |  |  |  |  |  |
| Cataract Dam | 0.96 | 0.96 | 0.96 | 0.95 | 0.95 | 0.95 | 0.31 |  |  |  |  |  |  |  |  |  |  |  |
| Abercrombie River | 0.95 | 0.96 | 0.95 | 0.93 | 0.93 | 0.93 | 0.27 | *0.04* |  |  |  |  |  |  |  |  |  |  |
| Lachlan River | 0.95 | 0.96 | 0.95 | 0.93 | 0.93 | 0.93 | 0.24 | *0.04* | *0.00* |  |  |  |  |  |  |  |  |  |
| Adjungbilly Creek | 0.98 | 0.99 | 0.97 | 0.96 | 0.95 | 0.96 | 0.24 | 0.10 | 0.13 | 0.13 |  |  |  |  |  |  |  |  |
| Cotter River | 0.97 | 0.97 | 0.96 | 0.96 | 0.96 | 0.96 | 0.45 | 0.51 | 0.49 | 0.48 | 0.51 |  |  |  |  |  |  |  |
| Murrumbidgee River | 0.97 | 0.98 | 0.97 | 0.95 | 0.95 | 0.95 | 0.22 | 0.34 | 0.35 | 0.29 | 0.39 | 0.50 |  |  |  |  |  |  |
| Dartmouth Lake | 0.94 | 0.94 | 0.94 | 0.93 | 0.94 | 0.93 | 0.39 | 0.06 | 0.11 | 0.12 | 0.06 | 0.40 | 0.29 |  |  |  |  |  |
| Hollands Creek | 0.95 | 0.96 | 0.95 | 0.93 | 0.93 | 0.93 | 0.24 | 0.06 | 0.12 | 0.11 | 0.07 | 0.49 | 0.27 | 0.05 |  |  |  |  |
| Sevens Creek | 0.94 | 0.95 | 0.95 | 0.93 | 0.93 | 0.93 | 0.29 | 0.14 | 0.18 | 0.18 | 0.18 | 0.47 | 0.39 | 0.04 | 0.11 |  |  |  |
| Hughes Creek | 0.95 | 0.96 | 0.95 | 0.93 | 0.93 | 0.93 | 0.25 | 0.13 | 0.16 | 0.15 | 0.17 | 0.47 | 0.33 | 0.04 | 0.07 | *0.04* |  |  |
| King Parrot Creek | 0.95 | 0.95 | 0.95 | 0.93 | 0.93 | 0.93 | 0.30 | 0.21 | 0.19 | 0.18 | 0.29 | 0.54 | 0.40 | 0.17 | 0.21 | 0.20 | 0.11 |  |
| Yarra River | 0.93 | 0.94 | 0.93 | 0.93 | 0.93 | 0.92 | 0.42 | 0.05 | 0.09 | 0.10 | 0.08 | 0.41 | 0.30 | 0.02 | 0.07 | *0.04* | 0.05 | 0.13 |

Table S10C. Pairwise population values of mtDNA Φ_ST._ All values significant at P<0.001 are in plain font, not significant in italic. Cells are shaded by their relative values; Names of populations with HNB lineages are shaded blue, MDB lineages- orange; Cataract River (not shaded) has both HNB and MDB lineages.

| Φ_ST_ mtDNA | Wheeny Creek | Glenbrook Creek | Little River | Cataract River | Cataract Dam | Abercrombie River | Lachlan River | Adjungbilly Creek | Cotter River | Murrumbidgee River | Dartmouth Lake | Hollands Creek | Sevens Creek | Hughes Creek | King Parrot Creek |
| --- | --- | --- | --- | --- | --- | --- | --- | --- | --- | --- | --- | --- | --- | --- | --- |
| Wheeny Creek |  |  |  |  |  |  |  |  |  |  |  |  |  |  |  |
| Glenbrook Creek | 1.00 |  |  |  |  |  |  |  |  |  |  |  |  |  |  |
| Little River | 0.67 | 0.58 |  |  |  |  |  |  |  |  |  |  |  |  |  |
| Cataract River | 0.43 | 0.45 | *0.21* |  |  |  |  |  |  |  |  |  |  |  |  |
| Cataract Dam | 0.85 | 0.85 | 0.72 | 0.41 |  |  |  |  |  |  |  |  |  |  |  |
| Abercrombie River | 1.00 | 1.00 | 0.81 | 0.52 | 0.73 |  |  |  |  |  |  |  |  |  |  |
| Lachlan River | *0.97* | 0.98 | 0.78 | 0.51 | 0.71 | *0.04* |  |  |  |  |  |  |  |  |  |
| Adjungbilly Creek | 0.97 | 0.97 | 0.87 | 0.56 | 0.56 | 0.96 | 0.94 |  |  |  |  |  |  |  |  |
| Cotter River | 1.00 | 1.00 | 0.85 | 0.49 | *0.36* | 1.00 | 0.98 | 0.76 |  |  |  |  |  |  |  |
| Murrumbidgee River | 1.00 | 1.00 | 0.87 | 0.51 | 0.55 | 1.00 | 0.97 | 0.94 | 1.00 |  |  |  |  |  |  |
| Dartmouth Lake | 0.91 | 0.89 | 0.82 | 0.54 | 0.28 | 0.86 | 0.85 | 0.49 | *0.07* | 0.78 |  |  |  |  |  |
| Hollands Creek | 0.83 | 0.83 | 0.69 | 0.40 | *0.03* | 0.72 | 0.69 | 0.51 | 0.28 | 0.56 | *0.15* |  |  |  |  |
| Sevens Creek | 0.92 | 0.92 | 0.78 | 0.43 | *0.14* | 0.87 | 0.85 | 0.66 | 0.51 | 0.76 | 0.26 | *0.07* |  |  |  |
| Hughes Creek | 0.76 | 0.80 | 0.66 | 0.38 | *0.21* | 0.56 | 0.53 | 0.67 | 0.58 | 0.40 | 0.55 | 0.25 | 0.33 |  |  |
| King Parrot Creek | 0.73 | 0.77 | 0.65 | 0.38 | *0.10* | 0.55 | 0.52 | 0.56 | 0.43 | 0.42 | 0.44 | *0.16* | *0.25* | *0.15* |  |
| Yarra River | 0.71 | 0.71 | 0.64 | 0.42 | *0.07* | 0.54 | 0.53 | 0.42 | 0.29 | 0.27 | 0.28 | *0.10* | *0.13* | *0.10* | *0.11* |

Appendix 11. Hierarchical analyses of genetic structure in Structure.

Runs were summarised using web-server Clumpak (Kopelman et al., 2015), which runs StructureHarvester (Earl and vonHoldt, 2012), performs averaging cluster probabilities using greedy algorithm with random input orders in Clumpp (Jakobsson and Rosenberg, 2007), and visualizes the output in Distruct (Rosenberg, 2004).

For Structure analysis of all 19 populations (870 individuals), mean likelihood increased up to K=14 (Evanno method suggested K=2 as mostly likely number of clusters; Fig. S11Ai). For K=2, K=3 and K=4 analyses, all replicate runs suggested the same pattern of subsequent population subdivision: K=2 split all individuals into HNB and MDB, with Cataract River showing widespread admixture (Fig. 1 of the manuscript, Fig. S11B), K=3 differentiated all individual from Cotter River from the remaining MDB individuals, and K=4 differentiated Cataract River and Cataract Dam (admixed with MDB cluster) from all other HNB populations. For K=5 to K=12 analyses, replicate runs varied in their assignment of geographic clusters, but for majority of replicates addition of clusters differentiated meaningful geographic groups of individuals. Of twenty K=12 replicate runs, seven consistently showed grouping presented in Figure 1 (also Fig. S11C top graph): Wollemi+ Wheeny+ Glenbrook+ Erskine+ Kowmung+ Little, Cataract River (admixed and sharing membership with Wheeny and Cataract Dam), Cataract Dam, Abercrombie+ Lachlan, Cotter, Adjungbilly, Murrumbidgee, Dartmouth+ Hollands, Sevens, Hughes, King Parrot, and Yarra. Two K=12 replicates with highest mean likelihoods differed: they split northern HNB (Wheeny and Wollemi) and southern HNB (Glenbrook+ Erskine+ Kowmung+ Little) populations, but assigned Adjungbilly+ Murrumbidgee to the same cluster (Fig. S11C, second graph). Eleven K=12 replicates (Fig. S11C, bottom two graphs) suggested that Adjungbilly had shared membership in two clusters, Abercrombie+ Lachlan and Murrumbidgee, and also split either Dartmouth (top) or Yarra (second) into two clusters. Analyses assuming K from 13 to 16 showed individuals within populations split between clusters.

For analysis of all 12 populations of MDB origin (671 individuals), mean likelihood increased up to K=11 (Evanno method suggested K=2; Fig. S11Aii). All replicates of K=2 analysis consistently split Cotter from the rest of the MDB (Fig. S11Di), and all replicates of K=3 analysis further assigned northern (Lachlan+ Abercrombie+ Cataract Dam+ Adjungbilly+ Murrumbidgee) and southern (all Murray River tributaries + Yarra) populations to separate clusters (with individuals from Abercrombie+Lachlan having ~1/4 of membership in southern MDB cluster. Replicates from K=4 and K=5 analyses did not show consistent structure, but all those of K=6 analysis did: they retained Cotter as a cluster, grouped Abercrombie+ Lachlan+ Adjungbilly, assigned Cataract Dam and Murrumbidgee to separate clusters (with Murrumbidgee individuals sharing ~1/4 membership in the Cataract Dam cluster), and split southern MDB populations into two admixed clusters, Dartmouth+ Holland+ Sevens and Hughes+ King Parrot+ Yarra. The maximum number of clusters that reflected geographically coherent structure (supported by 12/20 replicates) was K=10: this analysis split Abercrombie+ Lachlan from Cataract Dam, Adjungbilly, Cotter, Murrumbidgee, Dartmouth+ Hollands, Sevens, Hughes, King Parrot and Yarra (Fig. S11Di).

For analysis of southern MDB populations (375 individuals), Ln(K) increased for K=1 to K=7 (Evanno method suggested K=2; Fig. S11Aiii). Analysis assuming K=2 showed that majority of individuals from Dartmouth and Hollands had high membership in one of the clusters, King Parrot- in another, and those from Sevens, Hughes and Yarra had various levels of admixture (Fig. S11Dii). All 20 replicates from K=5 analysis consistently represented Dartmouth+Hollands, Sevens, Hughes, King Parrot and Yarra as separate clusters, with a reasonable number of admixed individuals in each (Fig. S11Dii). K=6 and K=7 analyses split population memberships between different clusters, but not in a way consistent with Nguyen et al. (2012)’s clusters, suggesting that previous structure with two genetic clusters within Dartmouth and Yarra was an artefact of Structure analyses.

Analysis of six HNB populations (all except Cataract River and Cataract Dam; 134 individuals) showed almost linear increase in likelihood with increase in K from 1 to 6 (Evanno method suggested K=6; Fig. S11Aiv). General lack of gene flow between northern and southern HNB was suggested by K=2 analysis, which showed one of the two genetic cluster prevalent in northern HNB (Wheeny and Wollemi, with individual memberships in this cluster being >89% and >71%, respectively) and absent in southern HNB (Little, Kowmung, Erskine; individual membership <15%); Glenbrook individuals shared no more than 25% membership in northern cluster (Fig. S11Diii). In K=6 analysis, individuals from all six populations belonged to their local genetic cluster, except a single individual in Little River had >90% membership in Kowmung River cluster, suggesting a long-distance dispersal event (Fig. S11Diii).

**References**

Cadwallader, PL. 1981. Past and present distributions and translocations of Macquarie perch Macquaria australasica (Pisces: Percichthyidae), with particular reference to Victoria. Proceedings of the Royal Society of Victoria 93:23-30.

Earl, D.A., vonHoldt, B.M., 2012. STRUCTURE HARVESTER: a website and program for visualizing STRUCTURE output and implementing the Evanno method. Conservation Genetics Resources 4, 359-361.

Faulks, L.K., D.M. Gilligan, and L.B. Beheregaray. 2011. The role of anthropogenic vs. natural in-stream structures in determining connectivity and genetic diversity in an endangered freshwater fish, Macquarie perch (Macquaria australasica). Evolutionary Applications 4 (4):589–601.

Jakobsson, M., Rosenberg, N.A., 2007. CLUMPP: a cluster matching and permutation program for dealing with label switching and multimodality in analysis of population structure. Bioinformatics (Oxf) 23, 1801-1806.

Kopelman, N.M., Mayzel, J., Jakobsson, M., Rosenberg, N.A., Mayrose, I., 2015. Clumpak: a program for identifying clustering modes and packaging population structure inferences across K. Molecular Ecology Resources.

Lintermans, Mark. 2007. Fishes of the Murray-Darling Basin: an introductory guide: Murray-Darling Basin Commission Canberra.

Rosenberg, N.A., 2004. DISTRUCT: a program for the graphical display of population structure. Mol Ecol Notes 4, 137-138.

Figure S11A. Plots of the mean (± SD) log probability of the data for each K from 1 to K_max_ over 20 runs and ΔK statistic for four hierarchical analyses of Macquarie perch genetic structure:

1. All 19 populations, N=870, K_max_=16


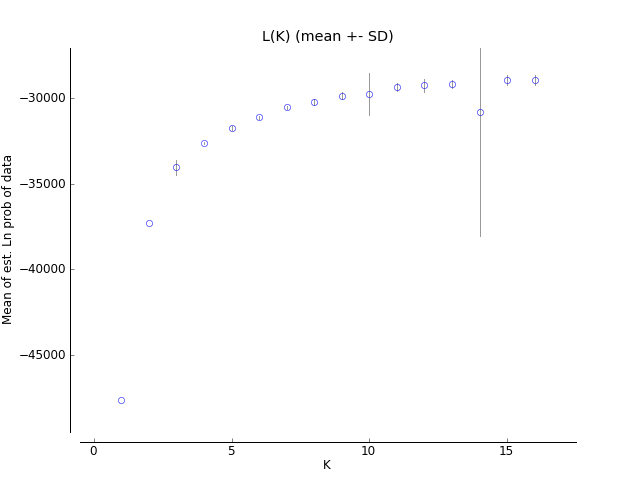

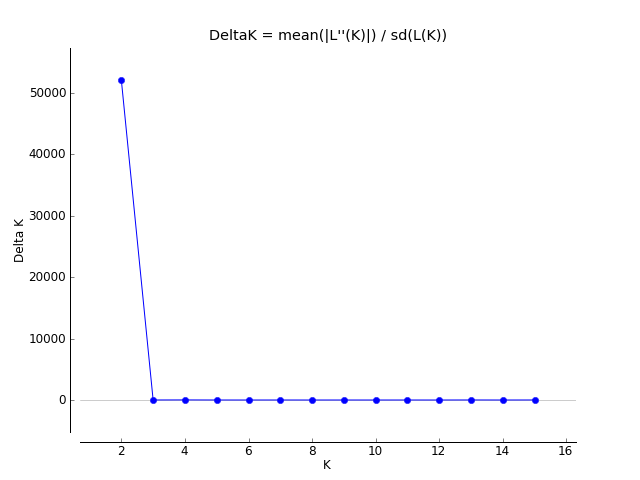


1. Ten MDB populations+ Cataract Dam+ Yarra, N=671, K_max_=16


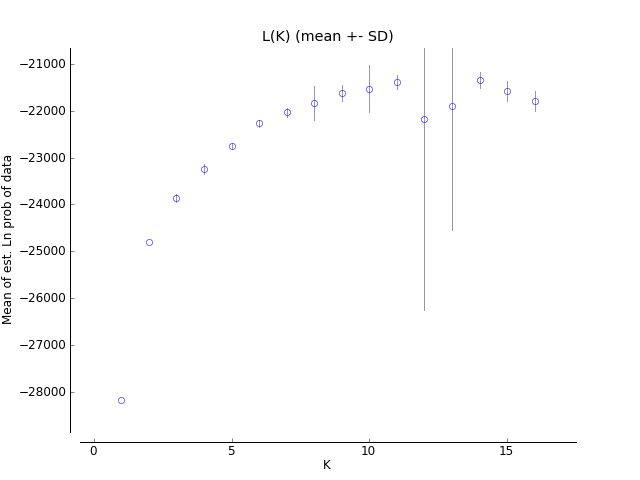

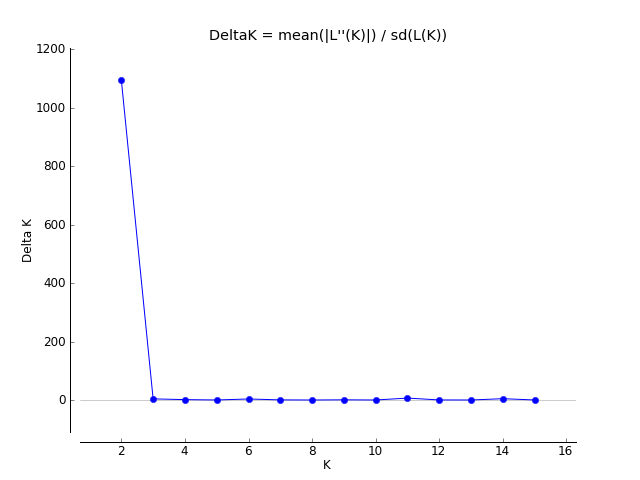


1. Six southern MDB populations, N=375, K_max_=7


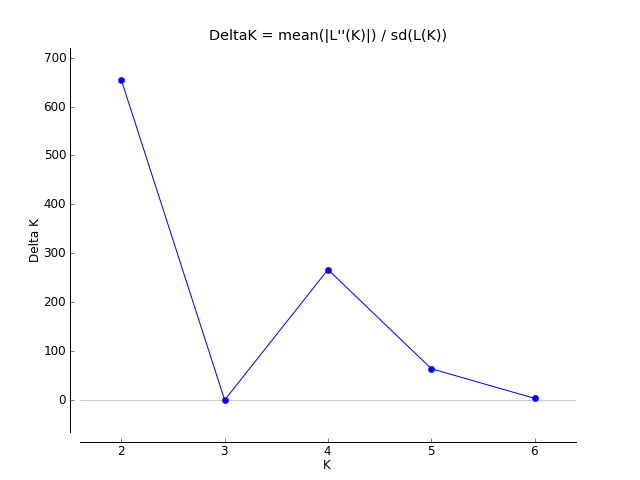

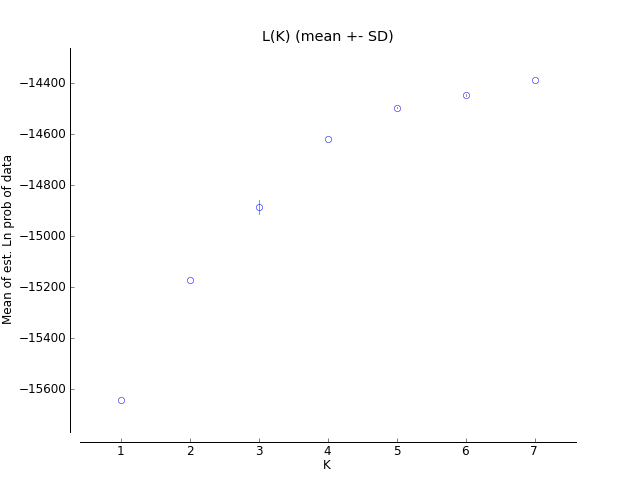


1. Six HNB populations, N=134, K_max_=7


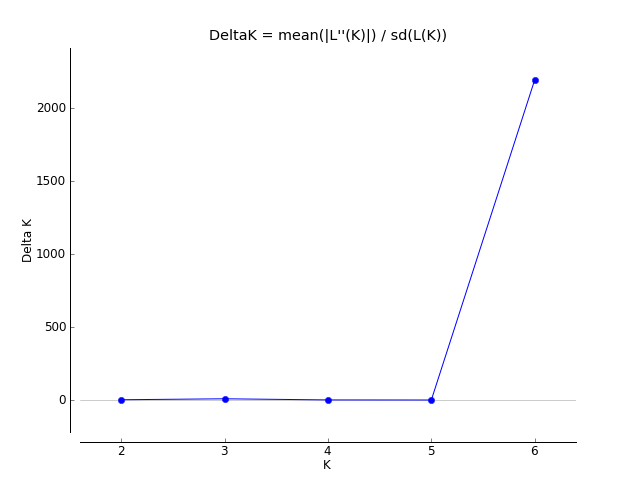

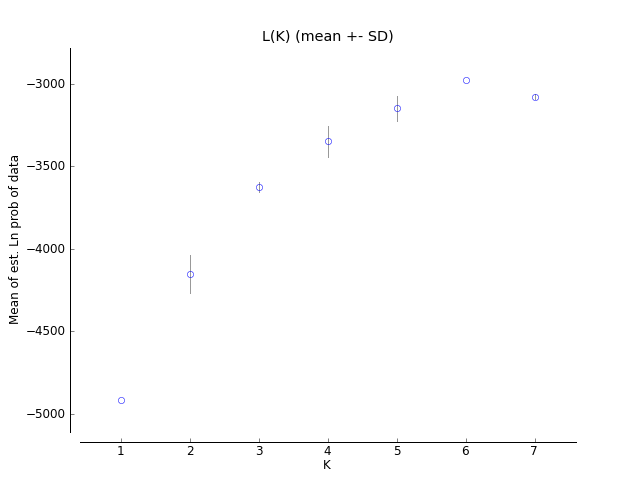


Figure S11B. Details of the Cataract River cluster memberships in the HNB (blue) and the MDB (orange) clusters (K=2 analysis of all 870 individuals, Fig. 1 of the main manuscript). Historical (1916) translocation of the MDB fish to Cataract Dam followed by fish dispersal over the dam wall into the Cataract River led to hybridization between the endemic HNB and MDB forms (Cadwallader 1981; Lintermans 2007; Faulks, Gilligan, and Beheregaray 2011). Individuals are arranged in the order of decreasing membership in the HNB cluster (Q-values ranging from 0.68 to 0.001). The last four individuals are potential first-generation migrants from Cataract Dam (translocated MDB lineage). Mitochondrial haplotype (Hap_ number) and its mitochondrial clade (MDB vs HNB) are mapped below each individual. A range of cluster membership values <0.5 suggests that hybridization occurs beyond first generation. Lack of correspondence between nuclear cluster membership (Q-values) and mitochondrial lineage suggests that hybridization is not sex-biased.


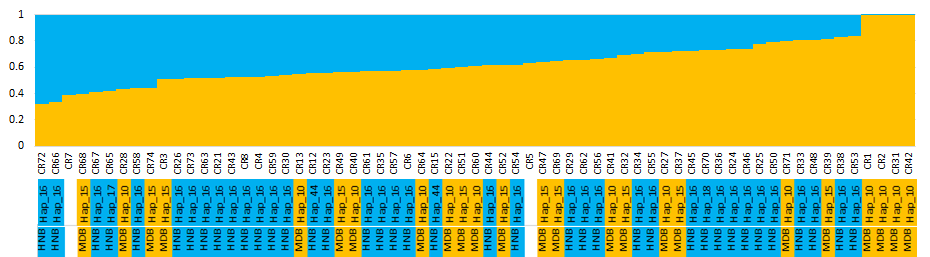


Figure S11C. Genetic clusters detected K12 Structure analyses; number of replicates (out of 20) supporting the structure and mean likelihood (LnProb) are shown on the left of each graph. The second graph has highest likelihood (in bold) but represents only 10% of replicates, the first graph has lower likelihood but is supported by the largest number of replicates (35%). Two last graphs have lowest likelihood and split membership of Adjungbilly and either Dartmouth or Yarra between two genetic clusters.


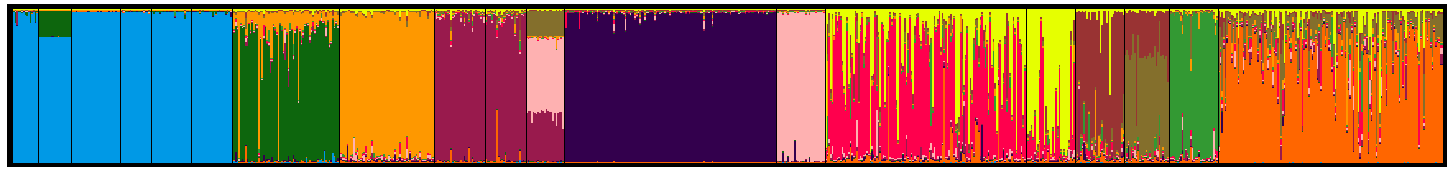


6/20

-29515


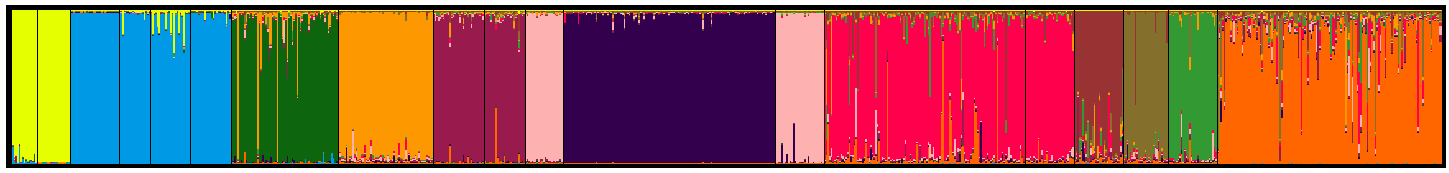


2/20

**-28562**

7/20

-29101


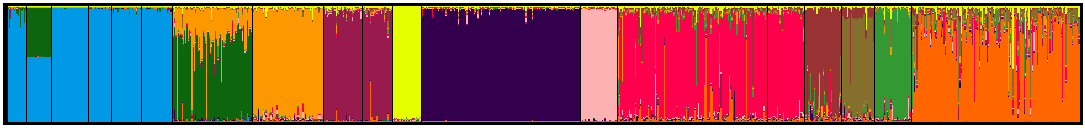

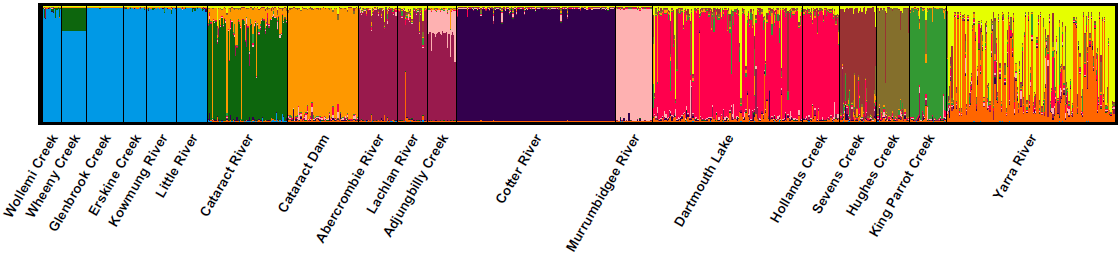


5/20

-29318

Figure S11D Memberships in genetic clusters of individuals from (i) twelve populations of MDB origin for K=2 and K=10, (ii) six southern MDB populations for K=2 and K=5, and (iii) six HNB populations (all except admixed Cataract River population) for K=2 and K=6.


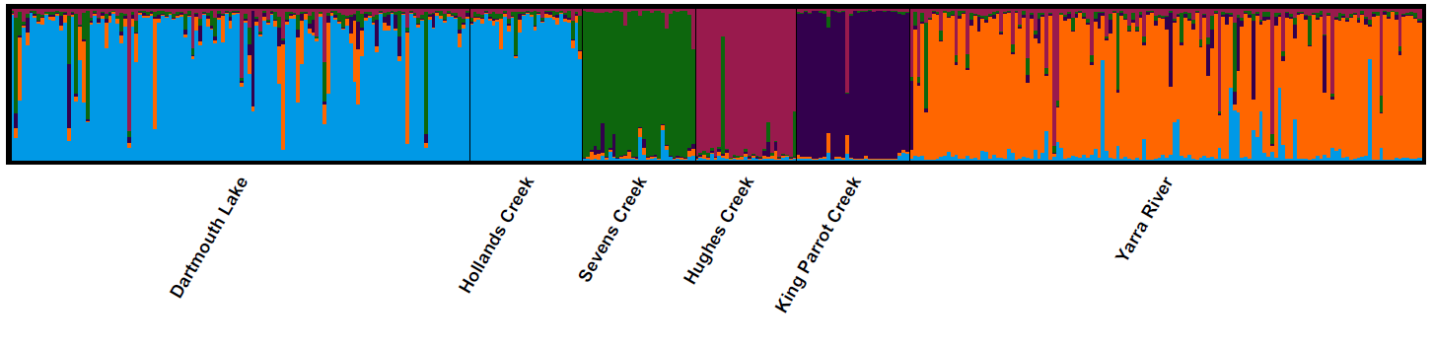

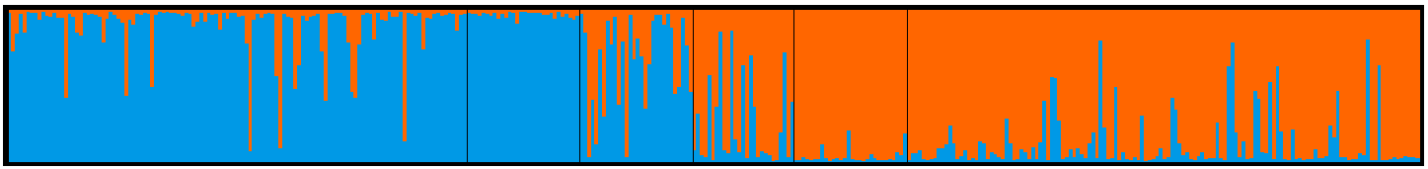

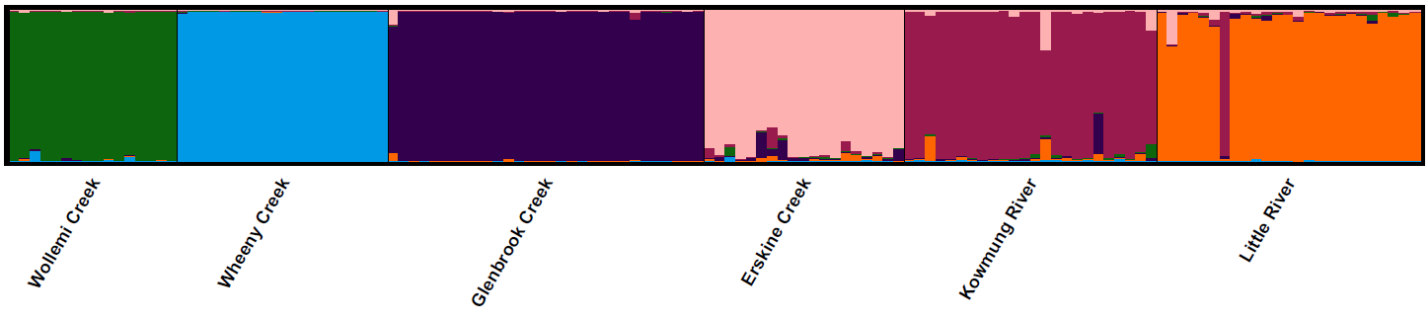

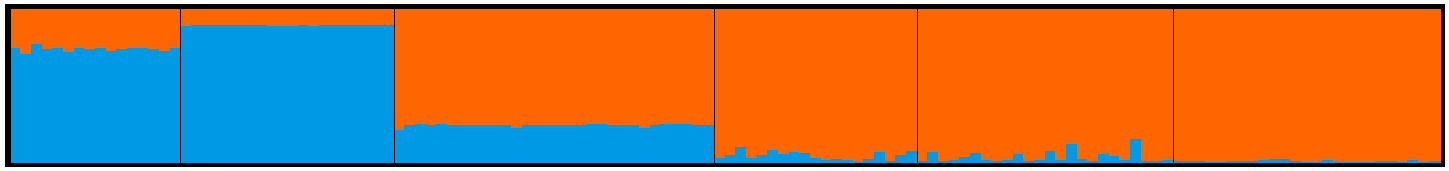

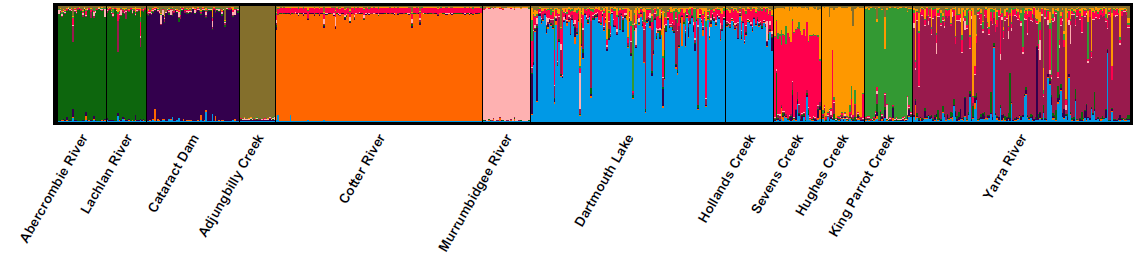

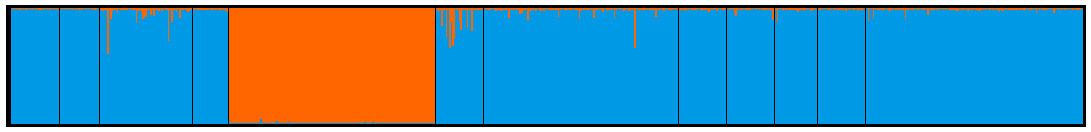


i. Populations of MDB origin

ii. Southern MDB populations

iii. HNB populations
