# Supplementary material for: Severe consequences of habitat fragmentation on genetic diversity of an endangered Australian freshwater fish: A call for assisted gene flow
Source: Evol Appl. 2017 May 11;10(6):531–50. doi: 10.1111/eva.12484 (PMC5469170; doi:10.1111/eva.12484)
Supplement: Supplementary file 3 [file EVA-10-531-s003.docx]

**Supporting Information**

Additional Supporting Information may be found in the online version of this article:

**Appendix 1.** Summary of previous Macquarie perch population genetic research relevant to planning genetic management

**Appendix 2.** Estimates of nuclear and mitochondrial genetic diversity and effective population sizes.

**Appendix 3.** Details of laboratory analyses and results of Hardy-Weinberg and linkage disequilibrium tests.

**Appendix 4.** Environmental variables used for modelling genetic diversity, pairwise correlations between them and variation at seven variables used for final analyses.

**Appendix 5.** Environmental model details, WinBUGS code and fitted relationships between HL and each of the seven environmental variables included in the model.

**Appendix 6.** Vortex simulations of population viability under two management scenarios (do nothing and 50 years of translocations) accounting for genetic factors.

**Appendix 7.** Distribution of the mitochondrial control region haplotypes.

**Appendix 8.** Maximum clade credibility tree from BEAST analysis of mitochondrial control region sequences.

**Appendix 9.** Analysis of the geographic distribution of microsatellite allele frequencies.

**Appendix 10.** Pairwise population values of microsatellite *F*_ST_, microsatellite *R*_ST_ and mtDNA Φ_ST_ and detailed results of SPAGeDi tests.

**Appendix 11.** Hierarchical analyses of genetic structure in Structure.
